# Developmental Validation of the Novel Five-Dye-Labeled Multiplex Autosomal STR Panel and Its Forensic Efficiency Evaluation

**DOI:** 10.3389/fgene.2022.897650

**Published:** 2022-05-31

**Authors:** Shimei Huang, Xiaoye Jin, Hongling Zhang, Haiying Jin, Zheng Ren, Qiyan Wang, Yubo Liu, Jingyan Ji, Meiqing Yang, Han Zhang, Xingkai Zheng, Danlu Song, Bingjie Zheng, Jiang Huang

**Affiliations:** ^1^ Department of Forensic Medicine, Guizhou Medical University, Guiyang, China; ^2^ Ningbo Health Gene Technologies Co.,Ltd, Ningbo, China

**Keywords:** STRs, CODIS, forensic research, developmental validation, complex paternity analysis

## Abstract

Short tandem repeats (STRs) are the most frequently used genetic markers in forensic genetics due to their high genetic diversities and abundant distributions in the human genome. Currently, the combined DNA index system is commonly incorporated into various commercial kits for forensic research. Some novel STRs that are different from the combined DNA index system were not only used to assess complex paternity cases but also could provide more genetic information and higher forensic efficiency in combination with those commonly used STRs. In this study, we validated forensic performance of a novel multiplex amplification STR panel to evaluate its sensitivity, species specificity, forensic application values, and so on. Obtained results revealed that the kit showed high sensitivity, and the complete allelic profile could be observed at 0.125 ng DNA sample. In addition, the kit possessed high species specificity, good tolerance to common inhibitors, and accurate genotyping ability. More importantly, STRs out of the kit displayed high discrimination power and probability of exclusion. To sum up, the novel kit presented in this study can be viewed as a promising tool for forensic human identification and complex paternity analysis.

## Introduction

Short tandem repeats (STRs), also known as microsatellites, are repeat sequences of 2–6 bp nucleotides and common genetic variants in the human genome (Edwards et al., 1992). STRs are also viewed as gold-standard genetic markers for forensic identity testing and parentage analysis owing to their high diversities and wide distributions in the human genome (Cheng et al., 2021; Vullo et al., 2021; Yang et al., 2021; Zhang et al., 2021). In 1998, Budowle et al. (1998) proposed a combined DNA index system (CODIS) that included 13 core STRs. Subsequently, a large number of DNA databases consisting of these 13 STRs were developed to aid in identifying suspects related to criminal cases. One potential problem is that adventitious matches of DNA typing may occur with the increase of DNA databases. In 2015, Hares (2015) selected seven additional STRs and added them to the original CODIS; they stated that the expanded CODIS could provide high discrimination power (PD) and reduce falsely matching rates of suspects. In the meantime, most of these STRs are also integrated into some commercial kits (Qu et al., 2021; Yin et al., 2021; Zhang et al., 2021). However, previous studies found that some STRs exhibited relatively low genetic diversities that went against forensic individual identification (Xie et al., 2015; Xiao et al., 2016; Tan et al., 2017). Recently, forensic researchers screened some novel STRs that exhibited high genetic polymorphisms in Chinese populations (Zhu et al., 2015; Li et al., 2017). On the one hand, these novel STRs could possess high cumulative PD. On the other hand, they could be used as additional loci for paternity testing when mutations of CODIS loci occur. More importantly, these novel STRs are also good for analyzing complex kinships like half-siblings.

In this study, 1 amelogenin gene and 26 autosomal STRs (D10S1248, D10S1435, D11S2368, D12S391, D13S325, D14S1434, D15S659, D16S539, D17S1301, D18S1364, D19S253, D1S1656, D20S482, D21S2055, D22GATA198B05, D22S1045, D2S441, D3S1744, D3S3045, D4S2366, D5S2800, D6S474, D6S477, D7S3048, D8S1132, and D9S1122) were developed into a multiplex panel named STRtyper-27 comp kit (HEALTH Gene Technologies, Zhejiang, China). Most of STRs in the novel kit are not overlapped with the expanded CODIS loci. Therefore, the kit is expected to provide more genetic information in combination with the extant upgraded CODIS loci. We also conducted validation studies of the kit to evaluate its overall performance based on the guideline of the Scientific Working Group on DNA Analysis Methods (https://1ecb9588-ea6f-4feb-971a-73265dbf079c.filesusr.com/ugd/4344b0_813b241e8944497e99b9c45b163b76bd.pdf). Furthermore, genetic distribution and forensic application value of the kit were assessed in the Guizhou Han population.

## Material and Methods

### Sample Information

We collected 312 bloodstain samples from unrelated healthy Guizhou Han individuals after obtaining their written informed consent. The 9948 and 9947A positive samples (1 ng/μL) were obtained from Promega Corporation (WI, United States ). DNA samples of common species including dog, pig, cow, sheep, chicken, mouse, rabbit, fish, and colibacillus were collected from the Animal Laboratory Center of Guizhou Medical University to assess species specificity of the STRtyper-27 comp kit. This research was performed in line with the guidelines of Guizhou Medical University and warranted by the Ethic Commission of Guizhou Medical University.

### DNA Amplification, Electrophoresis, and STR Typing

DNA sample of 1 ng was used to conduct the multiplex PCR of 27 loci according to the following specification. First, we prepared 10 μL PCR cocktail comprising 5 μL STRtyper-27 comp Master Mix, 2.5 μL STRtyper-27 comp Primer Mix, 2.5 μL ddH_2_O, and 1 ng DNA sample. Second, PCR was conducted on the GeneAmp PCR System 9700 (Applied Biosystems, Foster City, CA, United States ) under reaction conditions of initial denaturation at 95°C for 5 min; 28 cycles of 94°C for 10s, 61°C for 60s, and 70°C for 30 s; and 60°C for 15 min. Third, we mixed 1 μL amplified product/STRtyper-27 comp Allelic Ladder Mix with 8.75 μL deionized HiDi Formamide and 0.25 μL ILS-500 (HEALTH Gene Technologies) and then denatured the mixture at 95°C for 3 min, followed by chilling at 4°C for 3 min. Finally, the mixture was electrophoresed and separated by the 3500xL Genetic Analyzer (Thermo Fisher Scientific). STR typing of each locus was determined by the GeneMapper® ID-X Software v1.5 (Thermo Fisher Scientific) in comparison with the allelic ladder.

### PCR Condition Studies

Different annealing temperatures (56, 57, 58, 59, 60, 61, 62, 63, 64, 65, and 66°C), extension temperatures (65, 66, 67, 68, 69, 70, 71, 72, 73, 74, and 75°C), final extension time (5, 10, 15, 20, 25, and 30min), cycle numbers (25, 26, 27, 28, 29, and 30), and reaction volumes (5, 10, 15, 20, and 25 μL) were set to assess the performance of the kit in various PCR conditions. In addition, we also adjusted the concentrations of master mix (0.7×, 0.8×, 0.9×, 1.0×, 1.1×, 1.2×, and 1.3×) and primer mix (0.7×, 0.8×, 0.9×, 1.0×, 1.1×, 1.2×, and 1.3×) to explore the robustness of the kit for reagent fluctuations. The aforementioned experiments were performed by the 9948 DNA sample by adjusting the testing condition.

### Species Specificity and Sensitivity

Cross-reaction of the kit with non-human samples were evaluated by amplifying DNA samples of dog, pig, cow, sheep, chicken, mouse, rabbit, fish, and colibacillus.

The 9948 DNA sample was serially diluted to explore the detection lower limit of the kit: 1ng, 500, 250, 125, and 62.5 pg/μL. In addition, we also assessed the detection upper limit of the kit: 1, 2, 5, and 10 ng/μL.

### Size Precision and Mixture Studies

To evaluate size precision of the kit, we injected and detected the STRtyper-27 comp Allelic Ladder Mix by the 3500xL Genetic Analyzer 24 times.

Different ratios of 9948 and 9947A mixtures (1:1, 1:2, 1:4, 1:8, 1:19, 19:1, 8:1, 4:1, and 2:1) were constructed to assess the power of the kit to detect the mixture.

### Stability Study

In total, seven inhibitors including heme (1.0, 1.2, 1.4, 1.6, and 1.8 mM), humic acid (0.4, 0.43, 0.45, and 0.47 mg), EDTA (1, 5, 10, 15, 20, and 25 mM), melanin (0.5, 0.6, 0.7, 0.8, and 0.9 mg), Ca^2+^ (16, 18, 20, 22, and 24 mM), and tannin (1, 3, 5, and 7 mg) were collected to evaluate the tolerance of the kit to these inhibitors.

### Degraded and Case-Type Sample Studies

We used the ultraviolet (wave length: 254nm; power: 28W) to treat the positive DNA sample 9948 (1 ng/μL) to simulate the degraded sample at different time periods (0, 15, 30, 45, and 60 min). Then, these mocked samples were detected by the developed kit in triplicate. Here, the 50-relative fluorescence unit was used as the detecting threshold to determine the allele peak.

Common samples found in the forensic scene including cigarette, bloodstain, seminal stain, fingerprint swab, and blood swab were collected and detected by the developed kit. First, we extracted DNA samples from these biomaterials by the ML ultrafine magnetic bead extraction kit (Changchun Bokun Biotech Corporation, Jilin, China). Next, these DNA samples were detected by the developed kit and the STRtyper-32G kit (HEALTH Gene Technologies).

### Statistical Analysis

Allelic frequencies and forensic-related parameters including expected heterogeneity (He), observed heterogeneity (Ho), polymorphism information content (PIC), match probability (PM), PD, power of exclusion (PE), and typical paternity index (TPI) of 26 STR loci in the Guizhou Han population were estimated by the STRAF online tool v1.0.5 (Gouy and Zieger, 2017). Furthermore, Hardy–Weinberg equilibrium (HWE) and linkage disequilibrium (LD) analysis of these STRs in the Guizhou Han population were also assessed by the STRAF online tool v1.0.5.

## Results and Discussion

### Loci Information

**FIGURE 1:**
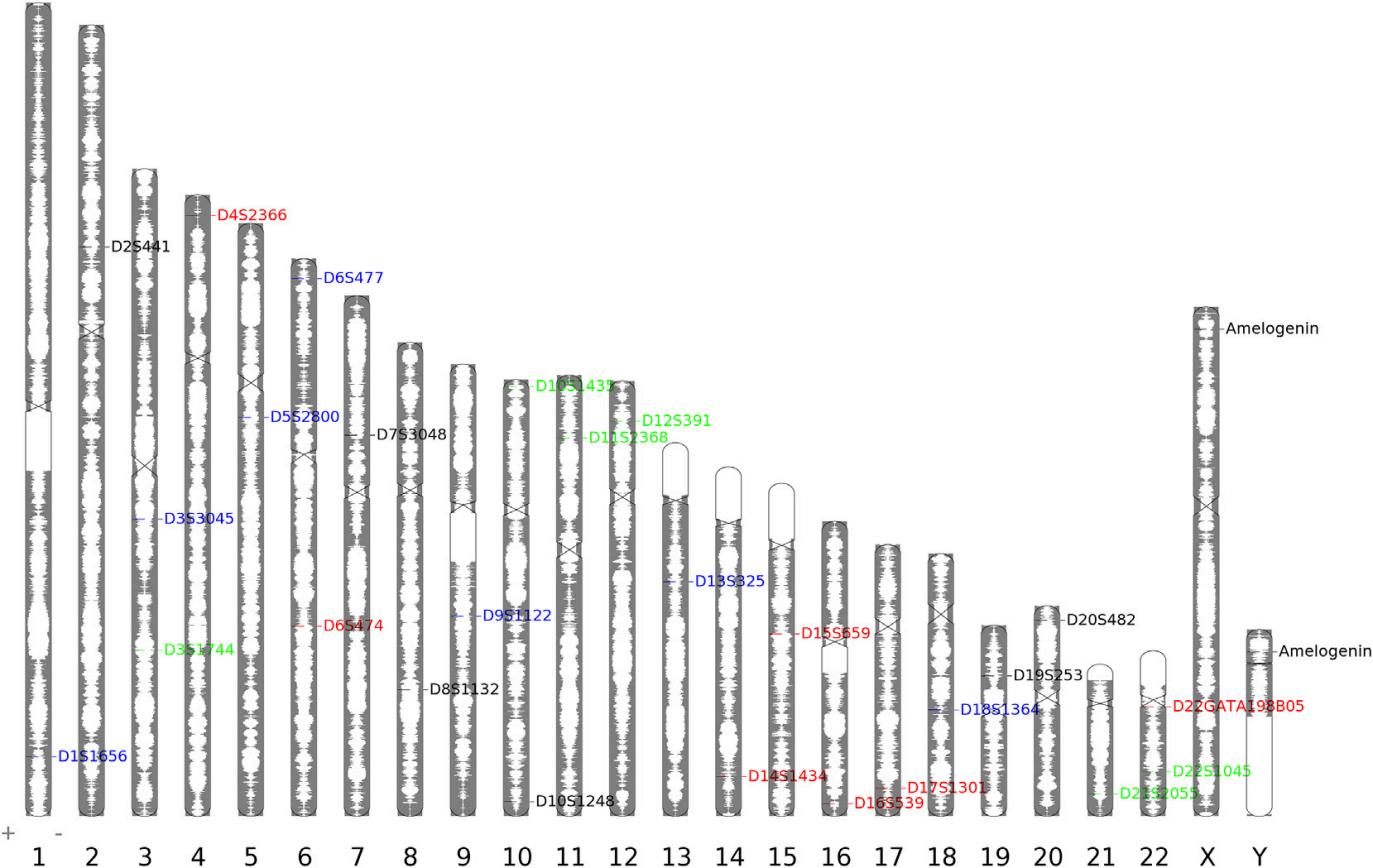
Loci information of 26 autosomal STRs and the amelogenin gene.

**FIGURE 2:**
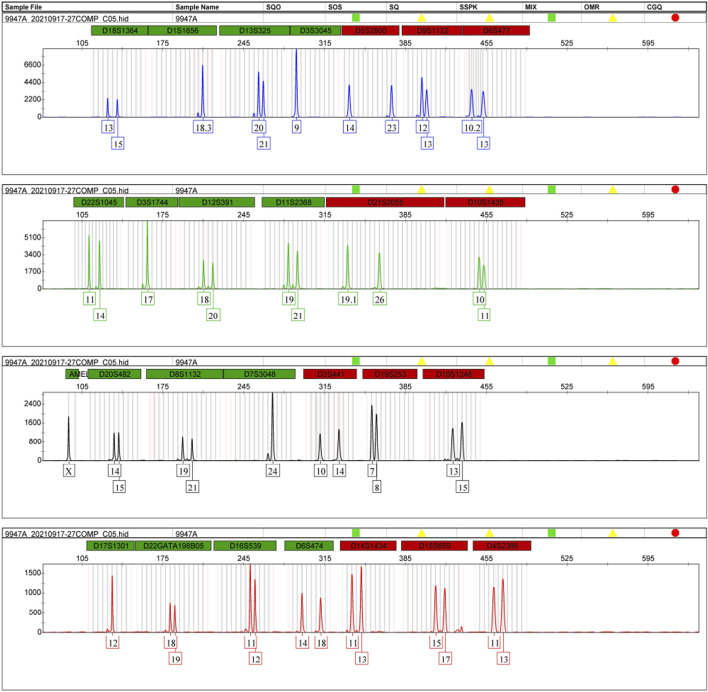
Allelic profile of the positive sample 9947A by the STRtyper-27 comp kit.

As shown in Figure 1, 27 loci of the STRtyper-27 comp kit were located on all chromosomes. These 27 loci were classified into four groups labeled by four dyes, respectively: D1S1656, D3S3045, D5S2800, D6S477, D9S1122, D13S325, and D18S1364 (FAM); D3S1744, D10S1435, D11S2368, D12S391, D21S2055, and D22S1045 (HEX); D4S2366, D6S474, D14S1434, D15S659, D16S539, D17S1301, and D22GATA198B05 (ROX); Amelogenin, D2S441, D7S3048, D8S1132, D10S1248, D19S253, and D20S482 (TAMRA). The allelic profile of 9947A positive sample is also given in Figure 2. The results showed that amplicon lengths of these loci distributed from 90 to 500 bp. Compared to other commercial STR kits (Hares, 2015; Zhu et al., 2015; Li et al., 2017; Ludeman et al., 2018; Zhong et al., 2019), we found that the kit in this study showed the most number (15) of overlapped loci using the Microreader 23SP kit (Supplementary Table S1). Even so, there were more than 10 novel STRs available in the developed kit. More importantly, the majority of loci presented in the kit were different from the expanded CODIS set. In addition, we found that physical distances between these novel STRs and those STRs (the expanded CODIS) on the same chromosomes were larger than 10 Mb (Supplementary Table S2), implying that these STRs could be viewed as independent loci from each other for forensic research. Even so, LD analyses of these STRs should be performed in the future. Anyway, we proposed that the kit in this study could be utilized as a high-efficient supplementary system for complex paternity analysis in parallel with the extant CODIS kits.

### PCR-Based Studies

The annealing temperature is the key factor for PCR because it determines whether the primer binds to the DNA template. Therefore, annealing temperature variations may exert some effects on the performance of the multiplex detection assay. We assessed amplification efficiency of the developed kit at different annealing temperatures, as shown in Supplementary Figure S1. We found that the kit displayed comparable amplification performance at 56–64°C. However, some alleles begun to drop out at 65°C. In addition, allele peak height showed significant decrease at 65 and 66°C. Consequently, researchers are not suggested to set higher annealing temperature than our recommended temperature in practical application.

For the extension temperature, it is also crucial for PCR performance of the STR kit because it is related to the DNA template extension reaction. We evaluated the influences of different extension temperatures on the amplification performance of the developed kit, as presented in Supplementary Figure S2. The results revealed that all alleles could be correctly typed at different extension temperatures. Thus, extension temperature variations did not show any negative effects on amplification efficiency of the STR system.

The final elongation reaction can be used to avoid non-template–depended adenylation that may give rise to minus A or shoulder peaks. Different final elongation times at 60°C were set to evaluate amplification performance of the developed kit, as given in Supplementary Figure S3. The results demonstrated that all alleles showed normal electrophoretic peaks at different elongation times, indicating that the kit was tolerant to elongation time variations.

Different cycle numbers were tested to explore the optimal condition for the developed kit. As shown in Supplementary Figure S4, all alleles could be observed at different cycle numbers. In addition, allele peak height gradually increased with the augment of cycle numbers. Some non-specific amplification products were also observed at higher cycle numbers. Given that more balanced peak height was seen at 29 cycle numbers, we suggested that 29 is the optimal cycle number. Even so, researchers may explore the best condition for samples of interest in their studies.

Primer, Taq DNA polymerase, and PCR buffer are indispensable components in PCR. The fluctuations of PCR reagents may occur due to pipetting errors, which may have negative impacts on amplification efficiency. A series of concentrations of the primer mix and master mix comprising Taq DNA polymerase, PCR buffer, and other essential components were tested to evaluate the robustness of the developed kit. As shown in Supplementary Figure S5, a full allelic profile could be observed at different concentrations of the primer mix. Moreover, more balanced peak heights among different alleles were observed at 1.0× primer mix. For different concentrations of the master mix, we also found that all alleles could be detected (Supplementary Figure S6). However, some noise peaks were also observed at higher concentrations of the master mix. Thus, we recommended 1.0× master mix as the optimal concentration.

In forensic practice, researchers may reduce PCR reaction volume for trace samples. Thus, we also assessed the impact of different reaction volumes on the amplification performance of the developed kit. As given in Supplementary Figure S7, a full allelic profile could be obtained at different reaction volumes. In addition, allele peak height decreased with the increase of reaction volume. From the aforementioned results, we proposed that the developed kit is robust for different reaction volumes.

### Sensitivity Studies

**FIGURE 3:**
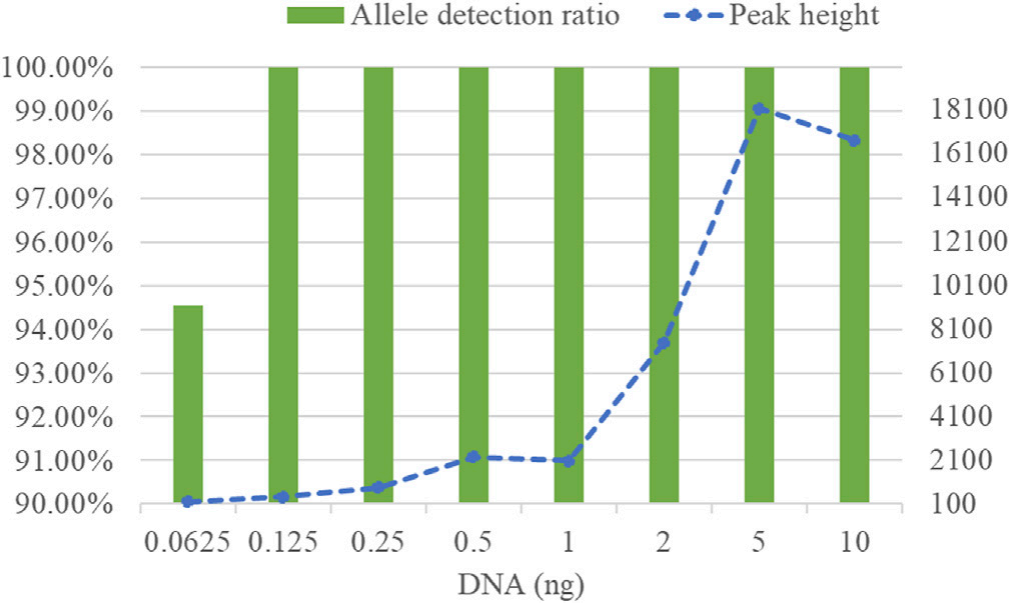
Allele detection ratios and allelic peak height of the STRtyper-27 comp kit at different amounts of DNA templates.

To determine detection limit of the developed kit, different quantities of DNA samples was amplified by the kit. Obtained results revealed that the complete allelic profile could be obtained from these diluted DNA samples except for 0.0625 ng DNA sample of which nearly 6% of alleles dropped out (Figure 3). In addition, as DNA quantity increased, allele peak height also gradually rose. Consequently, the developed kit is not recommended to detect those samples in which the amount of DNA was less than 0.0625 ng.

### Stability Studies

**FIGURE 4:**
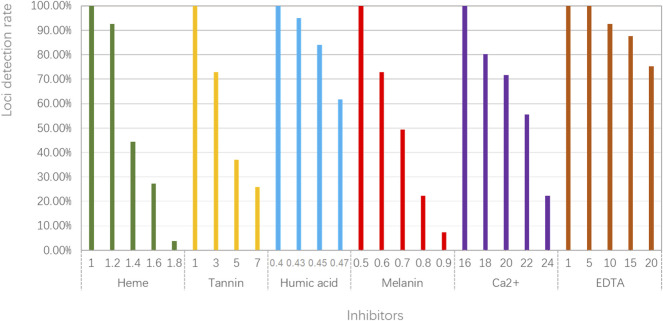
Stability studies of the STRtyper-27 comp kit for six common inhibitors.

To evaluate the tolerance of the developed kit to common inhibitors, we added different concentrations of inhibitors to the PCR reagents. Obtained results are given in Figure 4; Supplementary Figures 8–13. For heme, we found all loci could be detected at 1.0 mM. In addition, alleles of some loci began to drop out at larger concentrations of heme, especially for 1.4–1.8 mM. For tannin, nearly 30% of 27 loci missed at 3mg, and more loci dropped out at 5–7 mg. For humic acid, we found that the majority of 27 loci could be observed at different concentrations of humic acid. Similar results could be seen from different concentrations of EDTA. For melanin and Ca^2+^, the loci detection rate of the kit decreased with the increasing of melanin and Ca^2+^ concentrations. Overall, we stated that the kit performed relatively good tolerances for these inhibitors.

### Size Precision

**FIGURE 5:**
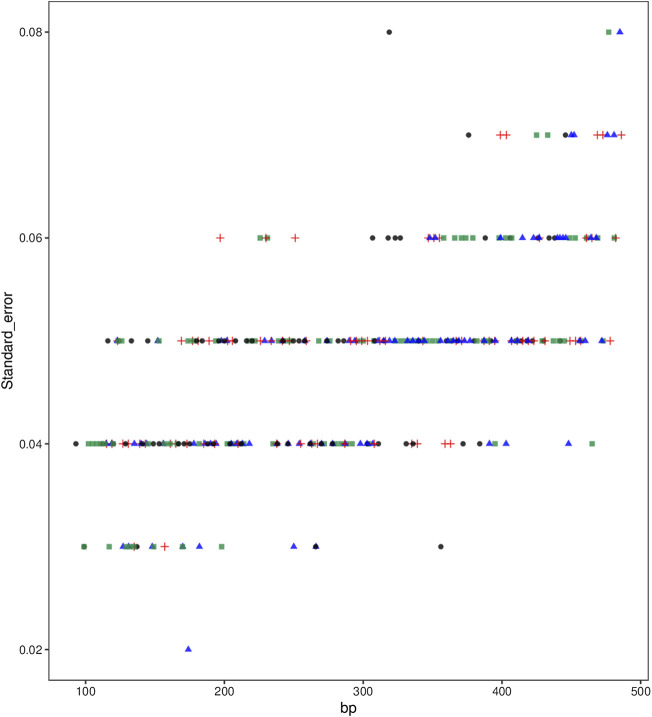
Size precision of the STRtyper-27 comp kit.

Allele size variations may occur between different runs even on the same equipment, which affects reliable and accurate typing. Accordingly, it is vital to evaluate size precision of the kit. As shown in Figure 5, standard deviations of all alleles ranged from 0.02 to 0.08, indicating relatively subtle size variations of the kit. Thus, we proposed that the kit could provide accurate and reliable allelic typing.

### Mixture Analysis

**FIGURE 6:**
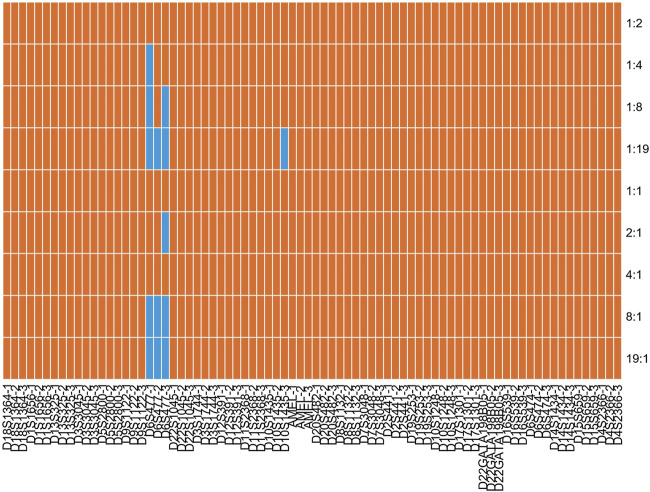
Allele detection results of the STRtyper-27 comp kit for different ratios of 9948 and 9947A samples. Orange and blue blocks indicated complete and partial alleles detected, respectively.

DNA mixtures are common biological samples in forensic research. Consequently, it is necessary to assess the efficiency of the developed kit to detect mixtures. Two positive samples (9948 and 9947A) were mixed at different ratios (1:1, 1:2, 1:4, 1:8, 1:19, 19:1, 8:1, 4:1, and 2:1) and detected by the developed kit in triplicate. The allelic profile of 9948 and 9947A samples are presented in Supplementary Table S3. We found that nearly all alleles could be observed at different mixed ratios (Figure 6 and Supplementary Figure S14). Even so, one allele of D6S477 locus dropped out when mixed ratios were 1:4, 1:8, 1:19, 2:1, 8:1, and 19:1. One allele of D10S1435 locus was also missing at 1:19 ratio. Moreover, an extra allele was observed for D6S477 locus at 19:1 ratio. Given that the extra allele was less than one repeat unit than targeted alleles, we postulated that it might be stutter peaks. Anyway, we proposed that the developed kit could be employed to dissolve mixtures of two individuals given that alleles of most loci could be detected at different ratios.

### Species Specificity

Non-human DNA may present in the forensic scene. It is critical to evaluate species specificity of the developed kit. As shown in Supplementary Figure S15, no allele peaks were seen at nine common species including dog, pig, cow, sheep, chicken, mouse, rabbit, fish, and colibacillus, suggesting that the kit was human-specific and could be used to detect human samples without the interfering from other non-human samples.

### Degraded and Case-Type Sample Studies

Allele detection results of 27 loci for mocked degraded samples are presented in Table 1; Supplementary Figure S16. A complete allelic profile of these 27 loci could be obtained from degraded samples at 15 min exposure time. When the exposure time increased to 30min, more than 80% alleles of these 27 loci could be detected. In addition, more than 60% alleles of these 27 loci were observed at 45 min exposure time. More importantly, approximately 30% alleles of these 27 loci were still obtained even though the exposure time increased to 60 min, indicating the kit displayed relatively good tolerance to these mocked degraded samples.

**TABLE 1:** Allele detection ratio and average peak height of 27 loci of the 9948 DNA sample exposed at different time periods of ultraviolet.

Time (min)	Allele detection ratio (%)	Average peak height
0	100.00	3,518
15	100.00	4,200
30	82.99	2,219
45	63.27	1762
60	28.57	171

For five collected samples (cigarette, bloodstain, seminal stain, fingerprint swab, and blood swab), we found that all alleles of 26 STRs could be observed. Furthermore, the same allelic profile of 14 overlapping STRs between the developed kit and the STRtyper-32G kit were also discerned from these samples. Thus, we proposed that the developed kit was suitable for detecting these common case samples.

### Population Studies

First, we conducted HWE and LD tests of 26 STRs in the Guizhou Han population, as given in Supplementary Tables 4–5. The results demonstrated that these loci did not deviate from HWE in the Guizhou Han population (*p* < 0.05). For LD of pairwise loci (Supplementary Table S5), we found that nine pairs deviated from linkage equilibrium after applying Bonferroni correction (*p* < 0.000154). There are some factors that may lead to LD of pairwise loci, like genetic linkage, mutation, genetic drift, and population inbreeding (Slatkin, 2008). However, these nine pairs that deviated from linkage equilibrium in the current study were located on different chromosomes. It was less likely that genetic linkage resulted in LD of these loci. Accordingly, it might be other reasons like mutation and genetic drift leading to LD of these nine pairwise loci, which needed to be further evaluated.

**FIGURE 7:**
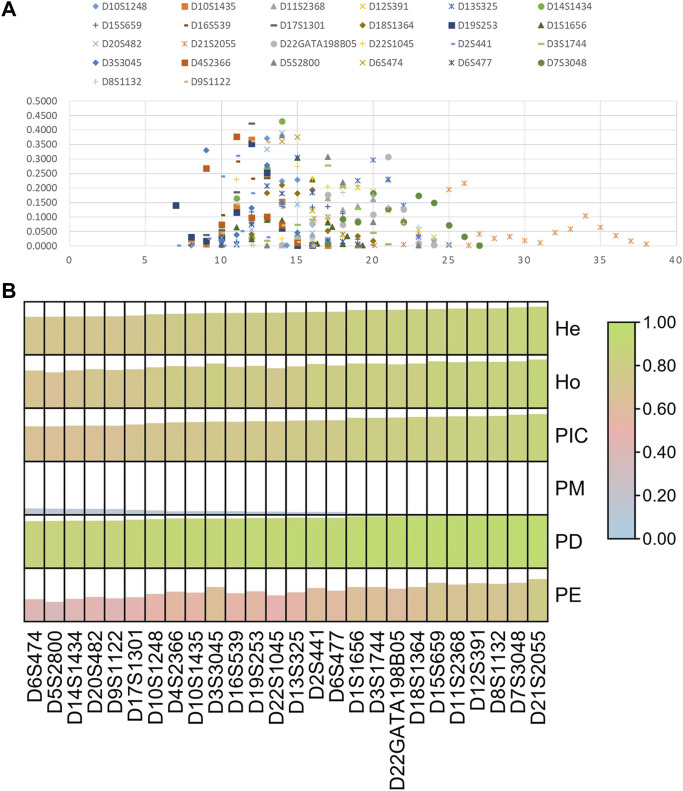
Allelic frequencies **(A)** and forensic parameters **(B)** of 26 STRs in the Guizhou Han population.

Next, we investigated allelic distributions and forensic parameters of 26 STRs in the Guizhou Han population, as shown in Figure 7; Supplementary Table S4. A total of 259 alleles were observed at these loci with the number of alleles per locus ranging from 6 (D6S474) to 21 (D21S2055). For forensic parameters, we found that these 26 loci displayed small PM values less than 0.15; their PE, PD, and TPI values ranged from 0.3740 to 0.7771, 0.8599 to 0.9727, and 1.4857 to 4.5882, respectively. For diversity indexes, the mean Ho, He, and PIC values were 0.7839, 0.7872, and 0.7549. In addition, PIC values of all loci were larger than 0.6, indicating that these loci possessed high genetic diversities in the Guizhou Han population. The cumulative PD and PE values of 26 STRs in the Guizhou Han population were 0.999 999 999 999 999 999 999 999 999 996961 and 0.999 999 999 90297, respectively. Compared to other commercial kits (Hares, 2015; Zhu et al., 2015; Li et al., 2017; Ludeman et al., 2018; Zhong et al., 2019), the developed kit could obtain better cumulative PD and PE values, implying that the kit could be viewed as a high-performance system for forensic identity testing and paternity analyses in the Guizhou Han population. Of note, most loci out of the kit were different from the expanded CODIS. Therefore, not only did these 26 STRs enhance discrimination efficiency for unrelated individuals by combining with the available commonly used STRs, but they could also be used to assess complex kinships.

## Conclusion

In this study, we validated the performance of the novel kit according to the specification of the Scientific Working Group on DNA Analysis Methods. The kit showed good species specificity, high sensitivity, and tolerance to six common inhibitors. In addition, the kit possessed good compatibility for the variations of PCR reagents and PCR conditions. More importantly, the kit displayed high forensic application values for forensic human identification and paternity testing. In conclusion, the developed kit could be viewed as a valuable tool for forensic research.

## Data Availability

The original contributions presented in the study are included in the article/Supplementary Material; further inquiries can be directed to the corresponding author.
