# Supplementary material for: Developmental Validation of the Novel Five-Dye-Labeled Multiplex Autosomal STR Panel and Its Forensic Efficiency Evaluation
Source: Front Genet. 2022 May 31;13:897650. doi: 10.3389/fgene.2022.897650 (PMC9194853; doi:10.3389/fgene.2022.897650)
Supplement: Supplementary file 1 [file DataSheet1.ZIP › SM/Figure legend.docx]

Supplementary Figure 1. Performance analysis of the STRtyper-27comp kit at different annealing temperatures.

Supplementary Figure 2. Performance analysis of the STRtyper-27comp kit at different extension temperatures.

Supplementary Figure 3. Performance analysis of the STRtyper-27comp kit at different extension times.

Supplementary Figure 4. Performance analysis of the STRtyper-27comp kit at different cycle numbers.

Supplementary Figure 5. Performance analysis of the STRtyper-27comp kit at different concentrations of Primer mix.

Supplementary Figure 6. Performance analysis of the STRtyper-27comp kit at different concentrations of Master mix.

Supplementary Figure 7. Performance analysis of the STRtyper-27comp kit at different reaction volumes.

Supplementary Figure 8. Performance analysis of the STRtyper-27comp kit at different concentrations of heme.

Supplementary Figure 9. Performance analysis of the STRtyper-27comp kit at different concentrations of tannin.

Supplementary Figure 10. Performance analysis of the STRtyper-27comp kit at different concentrations of humic acid.

Supplementary Figure 11. Performance analysis of the STRtyper-27comp kit at different concentrations of EDTA.

Supplementary Figure 12. Performance analysis of the STRtyper-27comp kit at different concentrations of melanin.

Supplementary Figure 13. Performance analysis of the STRtyper-27comp kit at different concentrations of Ca^2+^.

Supplementary Figure 14. Allele profile of 9948 and 9947A DNA samples at different mixed ratios.

Supplementary Figure 15. Species specificity of the STRtyper-27comp kit for common species.

Supplementary Figure 16. Allelic profile of 27 loci for the 9948 positive DNA sample exposed at different time of ultraviolet.
