# Supplementary material for: Developmental Validation of the Novel Five-Dye-Labeled Multiplex Autosomal STR Panel and Its Forensic Efficiency Evaluation
Source: Front Genet. 2022 May 31;13:897650. doi: 10.3389/fgene.2022.897650 (PMC9194853; doi:10.3389/fgene.2022.897650)

Project: 20210927-27COMP

GeneMapper® ID-X 1.5

Sample File	Sample Name	SQO	SOS	SQ	SSPK	MIX	OMR	CGQ
87-(1-1)-1 20210927-27COMP F08.hid	87-(1-1)-1							

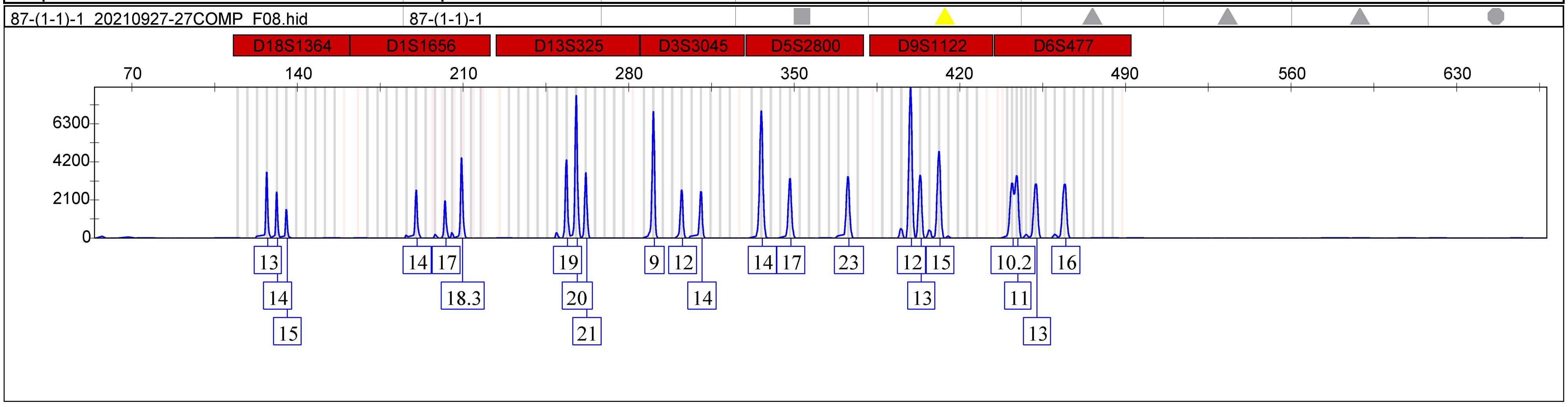

Sample File	Sample Name	SQO	SOS	SQ	SSPK	MIX	OMR	CGQ
87-(1-1)-1 20210927-27COMP F08.hid	87-(1-1)-1							

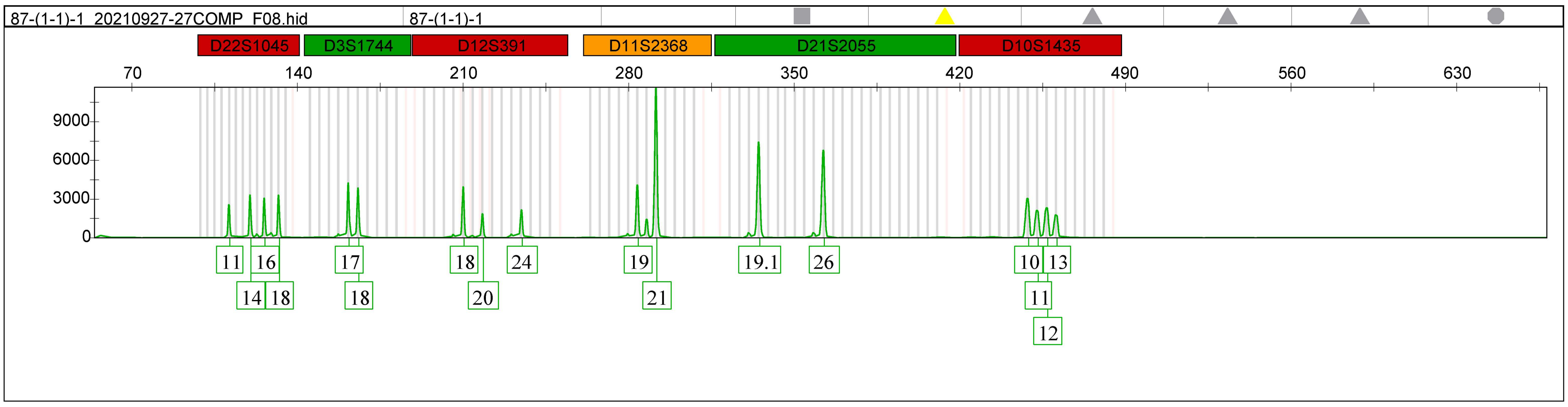

Sample File	Sample Name	SQO	SOS	SQ	SSPK	MIX	OMR	CGQ
87-(1-1)-1 20210927-27COMP F08.hid	87-(1-1)-1							

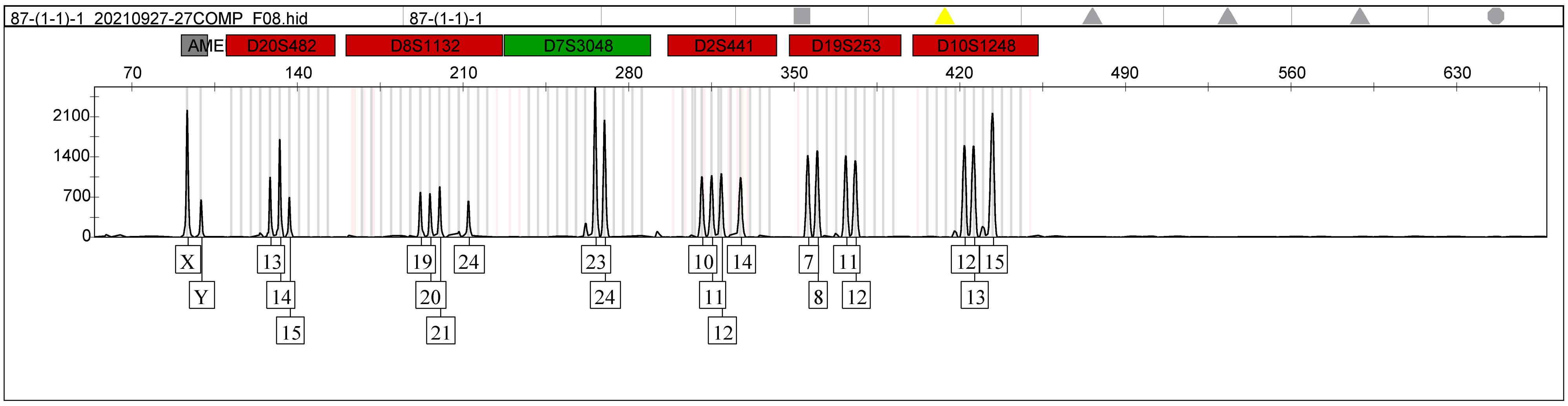

Sample File	Sample Name	SQO	SOS	SQ	SSPK	MIX	OMR	CGQ
87-(1-1)-1_20210927-27COMP_F08.hid	87-(1-1)-1							

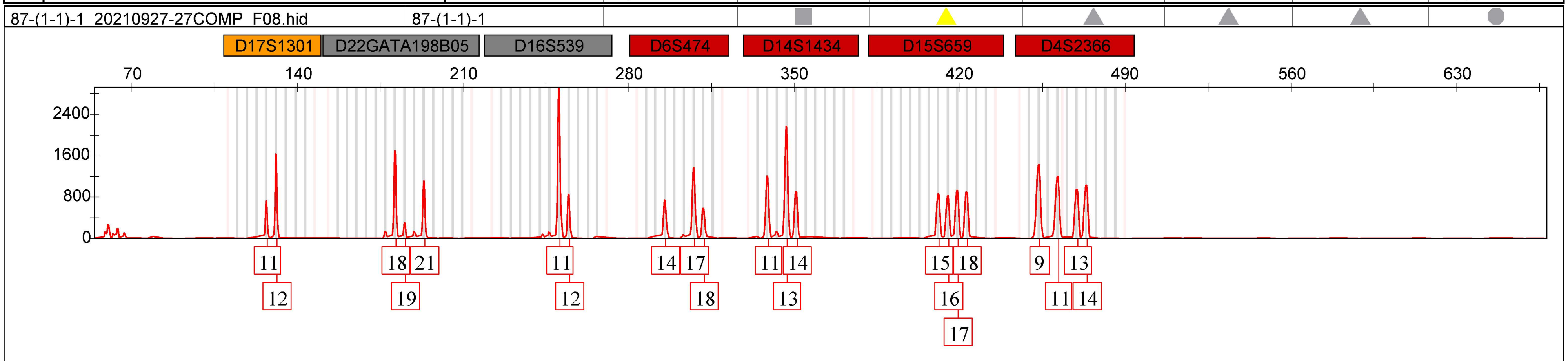

Sample File	Sample Name	SQO	SOS	SQ	SSPK	MIX	OMR	CGQ
87-(1-1)-2_20210927-27COMP_G08.hid	87-(1-1)-2							

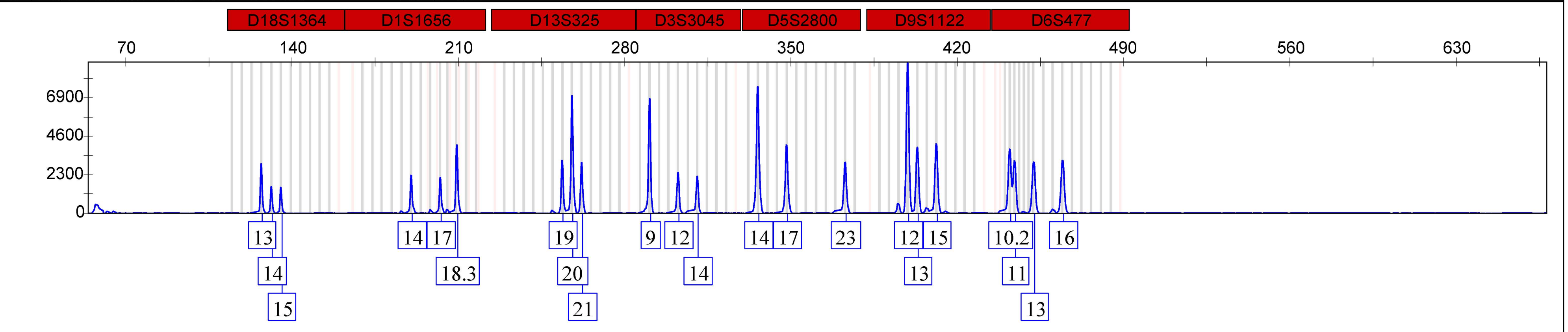

Sample File	Sample Name	SQO	SOS	SQ	SSPK	MIX	OMR	CGQ
87-(1-1)-2_20210927-27COMP_G08.hid	87-(1-1)-2							

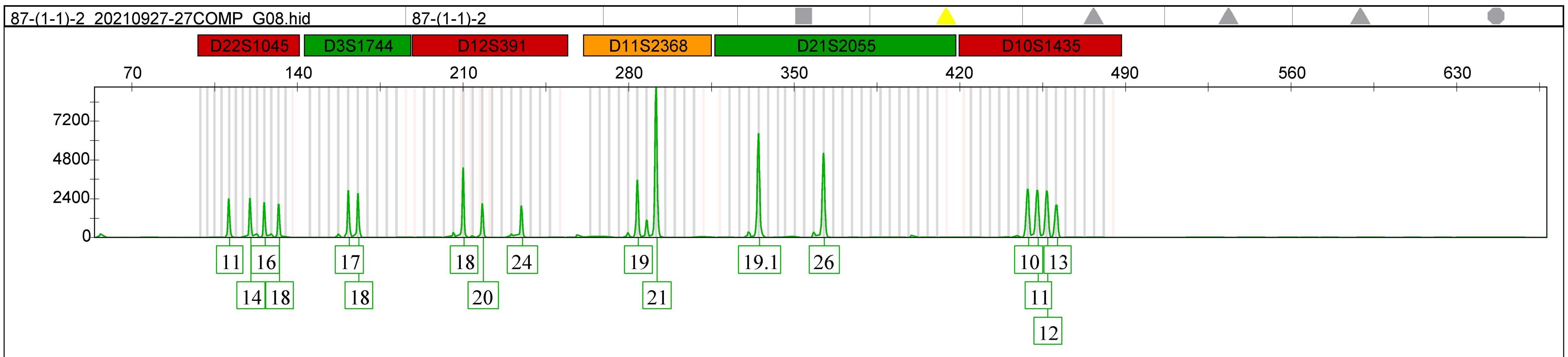

GeneMapper® ID-X 1.5

Sample File	Sample Name	SQO	SOS	SQ	SSPK	MIX	OMR	CGQ
87-(1-1)-2_20210927-27COMP_G08.hid	87-(1-1)-2							

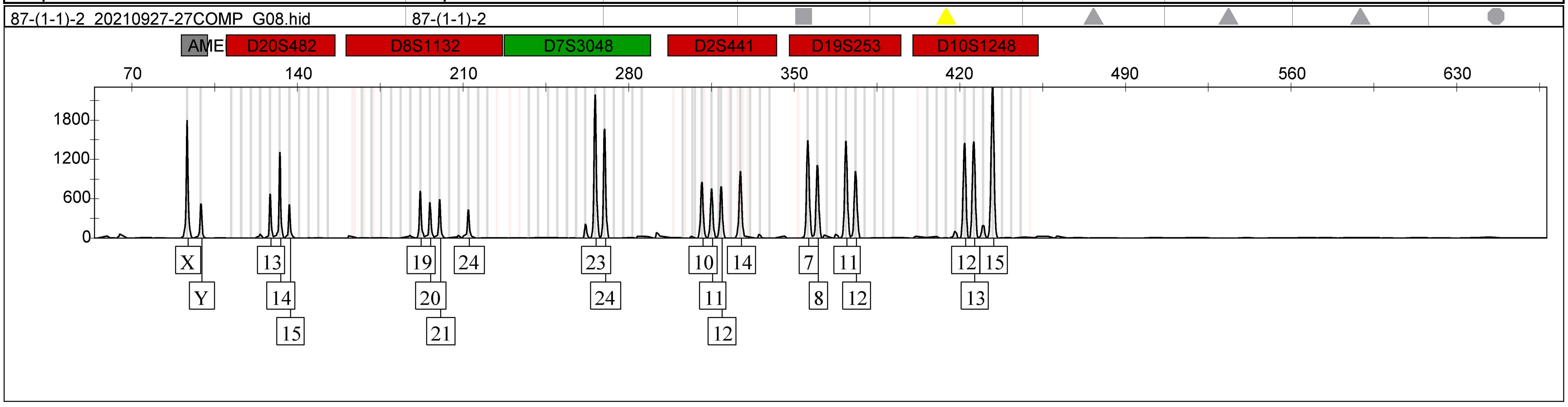

Sample File	Sample Name	SQO	SOS	SQ	SSPK	MIX	OMR	CGQ
87-(1-1)-2_20210927-27COMP_G08.hid	87-(1-1)-2							

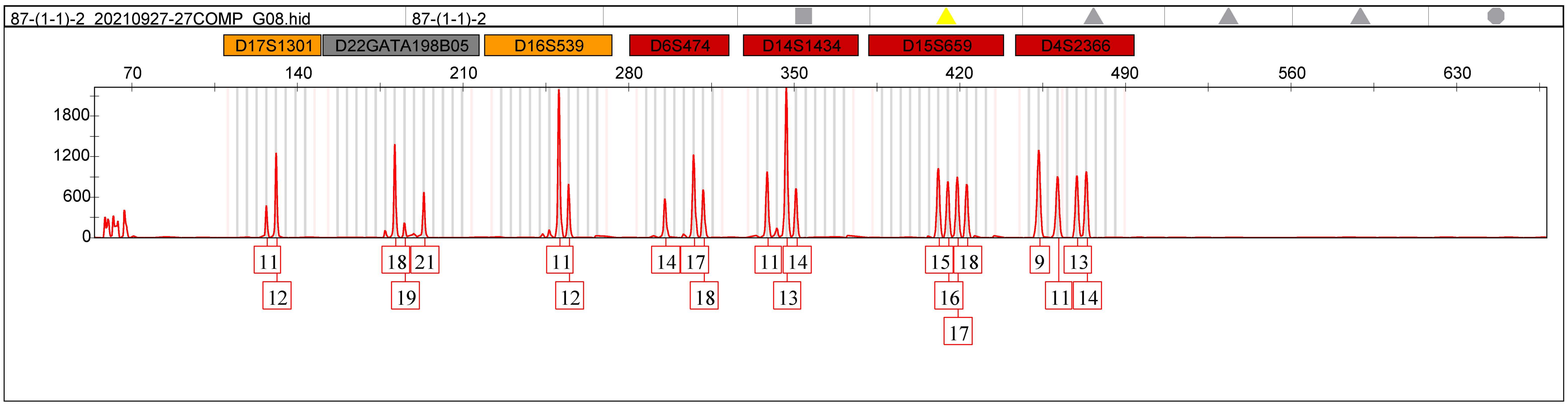

Sample File	Sample Name	SQO	SOS	SQ	SSPK	MIX	OMR	CGQ
87-(1-1)-3_20210927-27COMP_H08.hid	87-(1-1)-3							

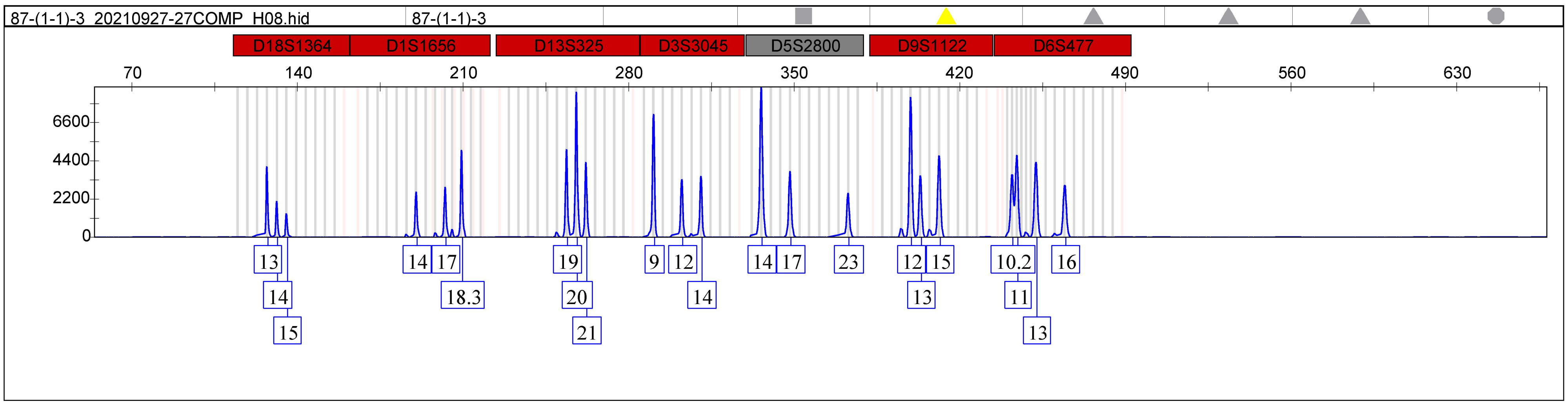

GeneMapper® ID-X 1.5

Sample File	Sample Name	SQO	SOS	SQ	SSPK	MIX	OMR	CGQ
87-(1-1)-3_20210927-27COMP_H08.hid	87-(1-1)-3							

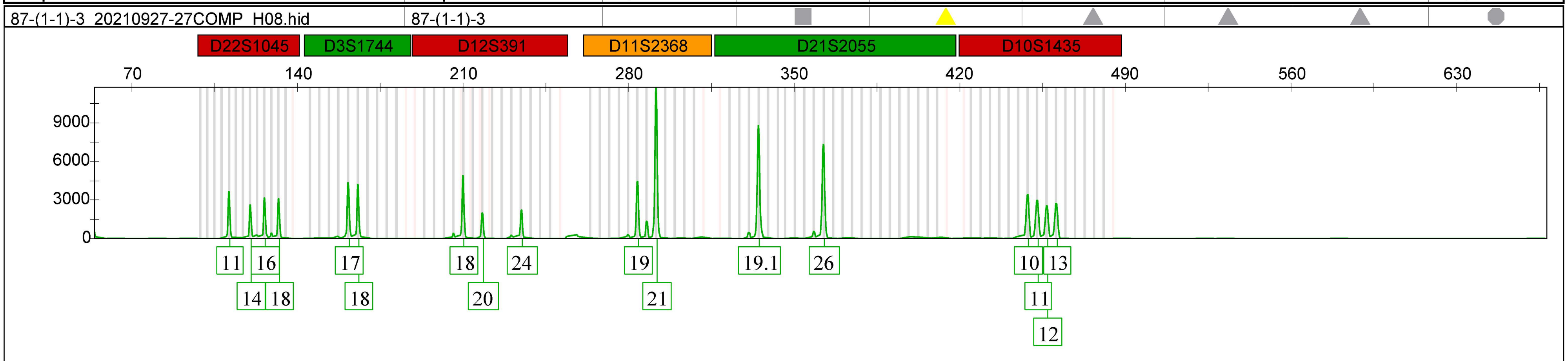

Sample File	Sample Name	SQO	SOS	SQ	SSPK	MIX	OMR	CGQ
87-(1-1)-3_20210927-27COMP_H08.hid	87-(1-1)-3							

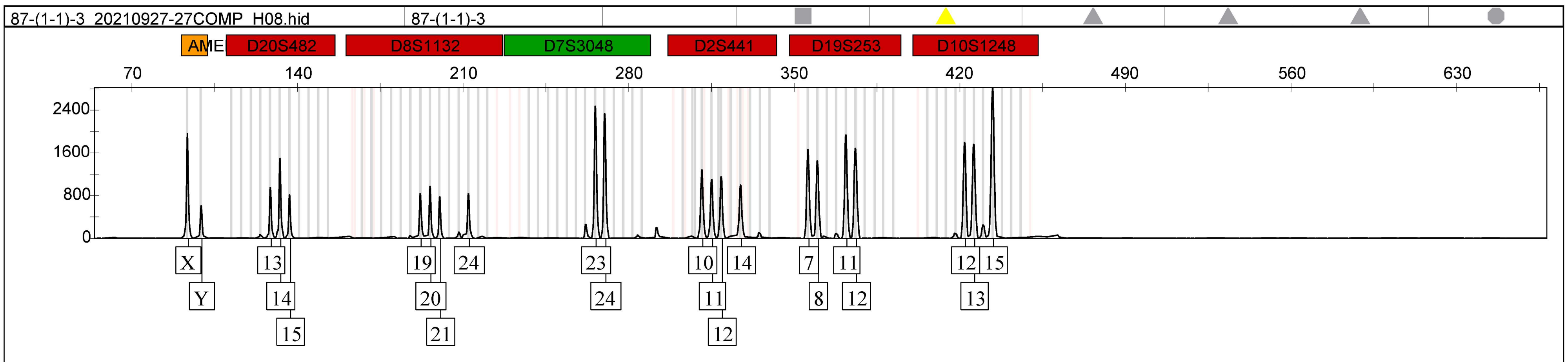

Sample File	Sample Name	SQO	SOS	SQ	SSPK	MIX	OMR	CGQ
87-(1-1)-3_20210927-27COMP_H08.hid	87-(1-1)-3							

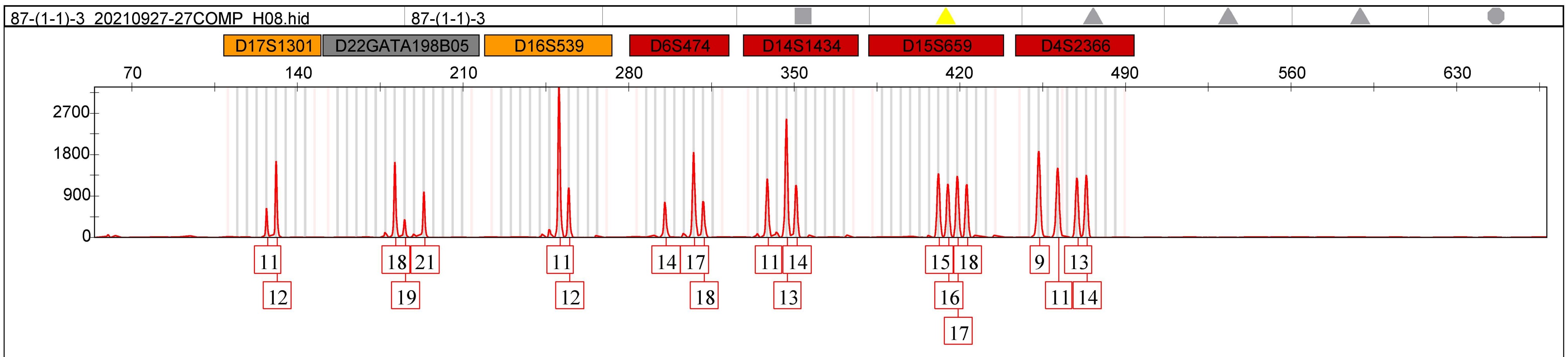

Sample File	Sample Name	SQO	SOS	SQ	SSPK	MIX	OMR	CGQ
87-(1-2)-1_20210927-27COMP_A09.hid	87-(1-2)-1							

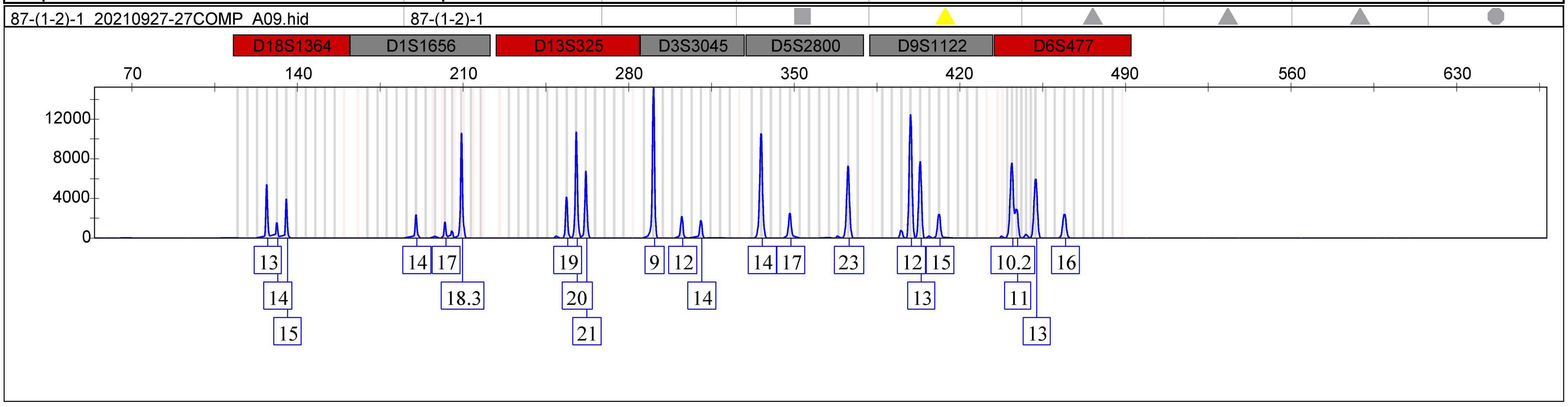

87-(1-2)-1_20210927-27COMP_A09.hid 87-(1-2)-1

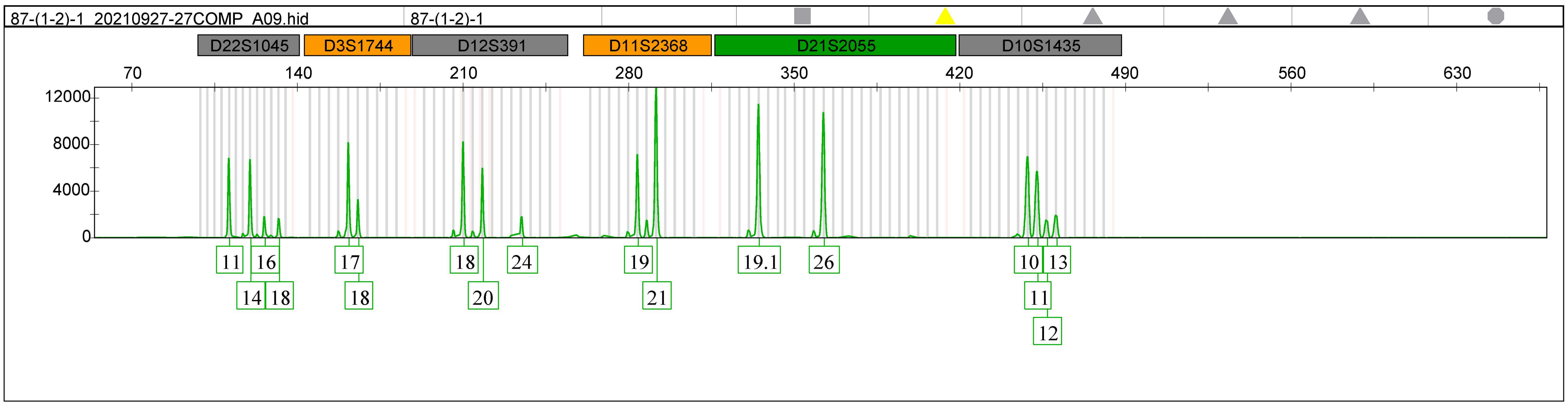

87-(1-2)-1_20210927-27COMP_A09.hid 87-(1-2)-1

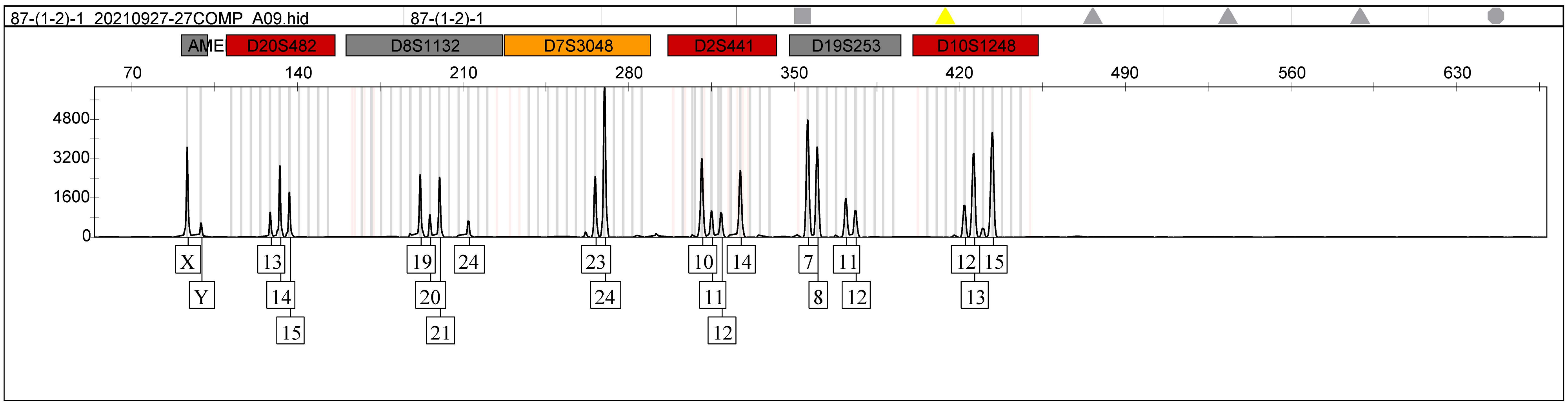

Project: 20210927-27COMP

GeneMapper® ID-X 1.5

Sample File	Sample Name	SQO	SOS	SQ	SSPK	MIX	OMR	CGQ
87-(1-2)-1_20210927-27COMP_A09.hid	87-(1-2)-1							

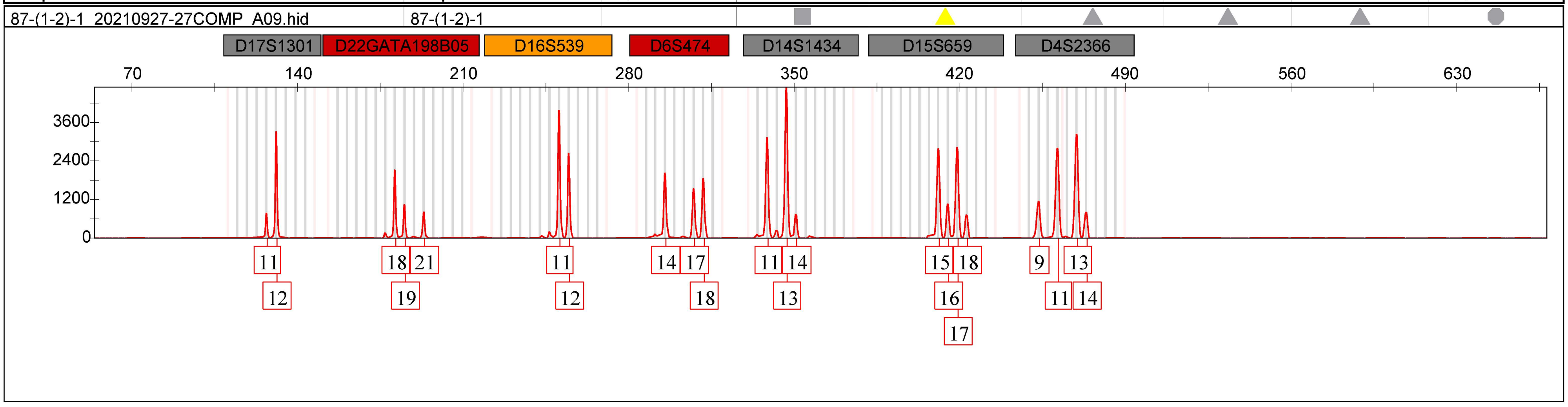

Sample File	Sample Name	SQO	SOS	SQ	SSPK	MIX	OMR	CGQ
87-(1-2)-2_20210927-27COMP_B09.hid	87-(1-2)-2							

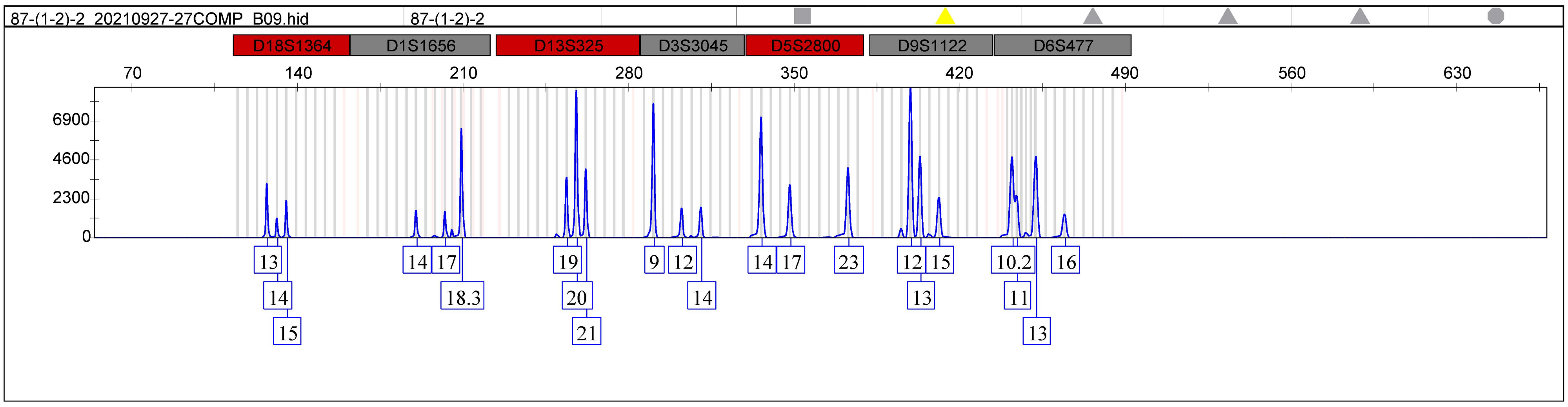

Sample File	Sample Name	SQO	SOS	SQ	SSPK	MIX	OMR	CGQ
87-(1-2)-2_20210927-27COMP_B09.hid	87-(1-2)-2							

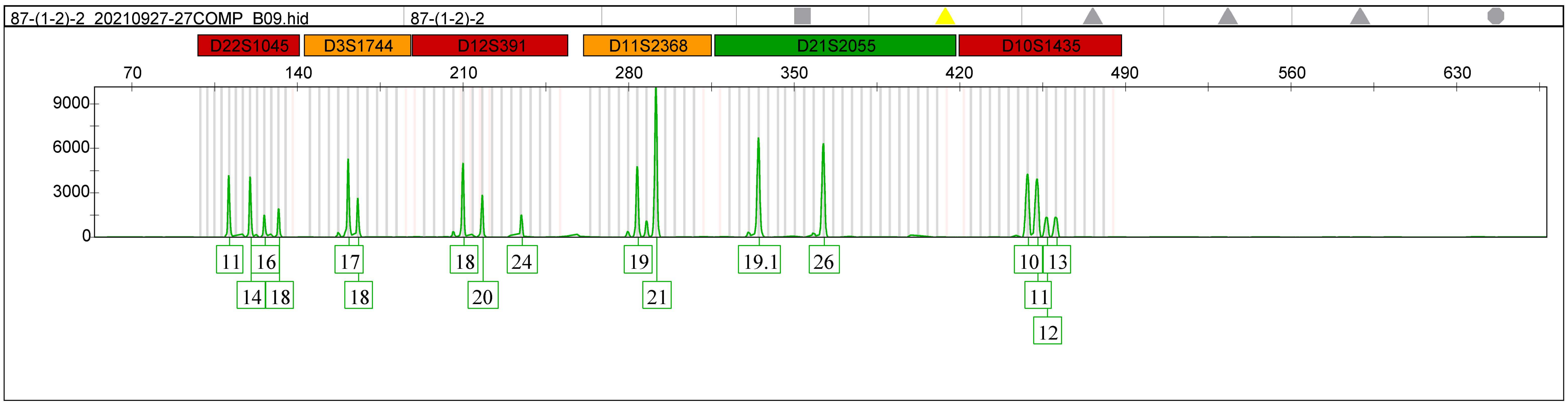

Project: 20210927-27COMP

GeneMapper® ID-X 1.5

Sample File	Sample Name	SQO	SOS	SQ	SSPK	MIX	OMR	CGQ
87-(1-2)-2_20210927-27COMP_B09.hid	87-(1-2)-2							

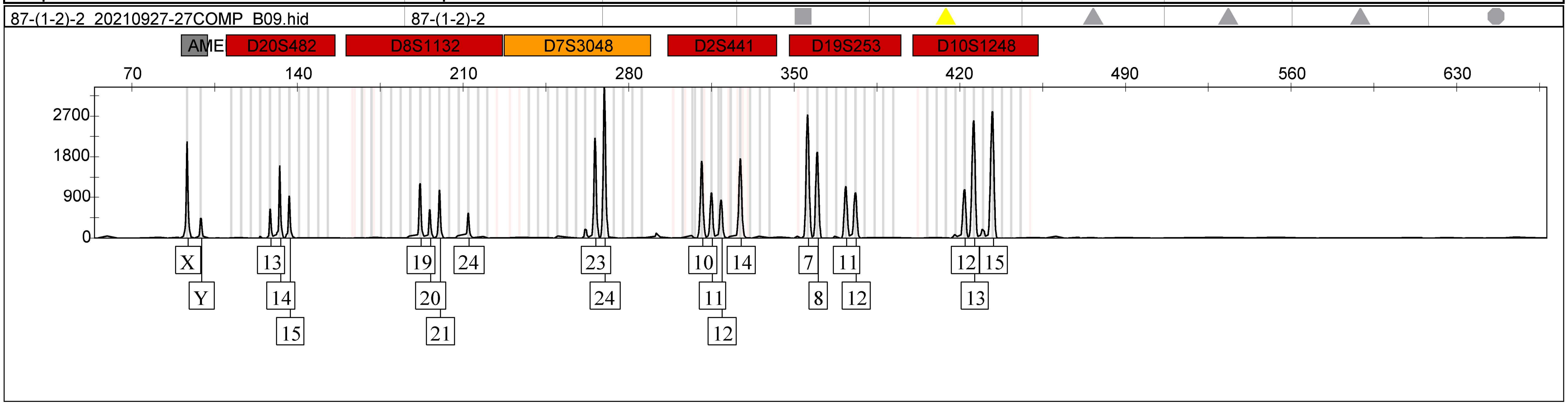

Sample File	Sample Name	SQO	SOS	SQ	SSPK	MIX	OMR	CGQ
87-(1-2)-2_20210927-27COMP_B09.hid	87-(1-2)-2							

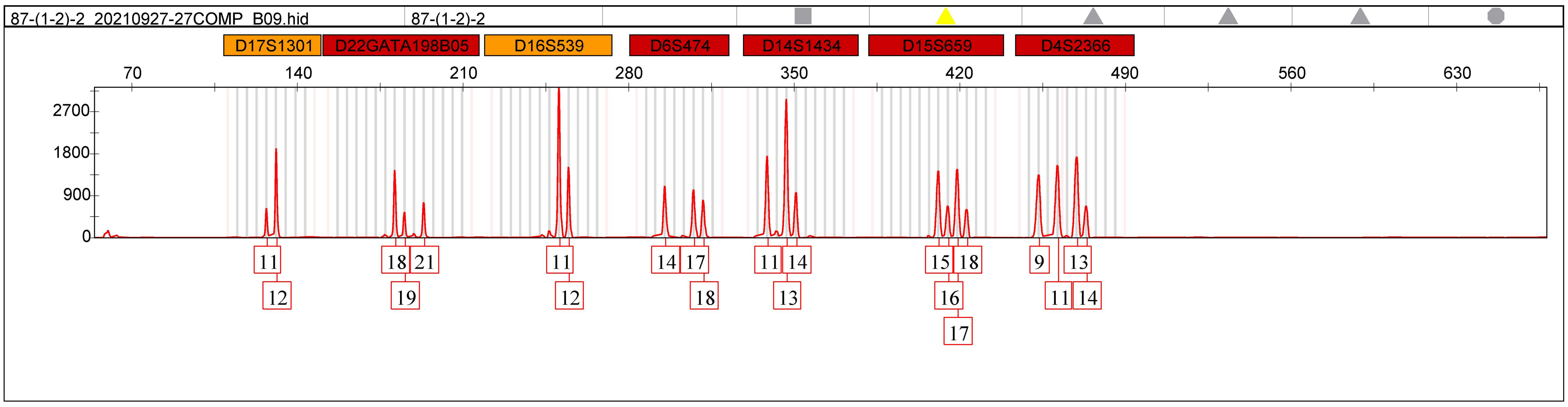

Sample File	Sample Name	SQO	SOS	SQ	SSPK	MIX	OMR	CGQ
87-(1-2)-3_20210927-27COMP_C09.hid	87-(1-2)-3							

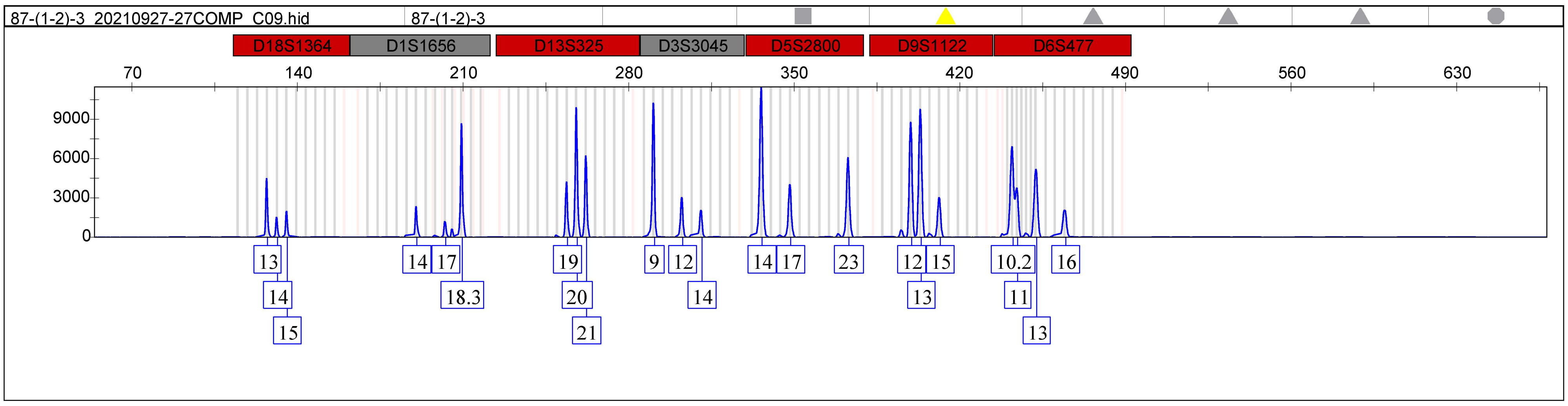

Project: 20210927-27COMP

GeneMapper® ID-X 1.5

Sample File	Sample Name	SQO	SOS	SQ	SSPK	MIX	OMR	CGQ
87-(1-2)-3_20210927-27COMP_C09.hid	87-(1-2)-3							

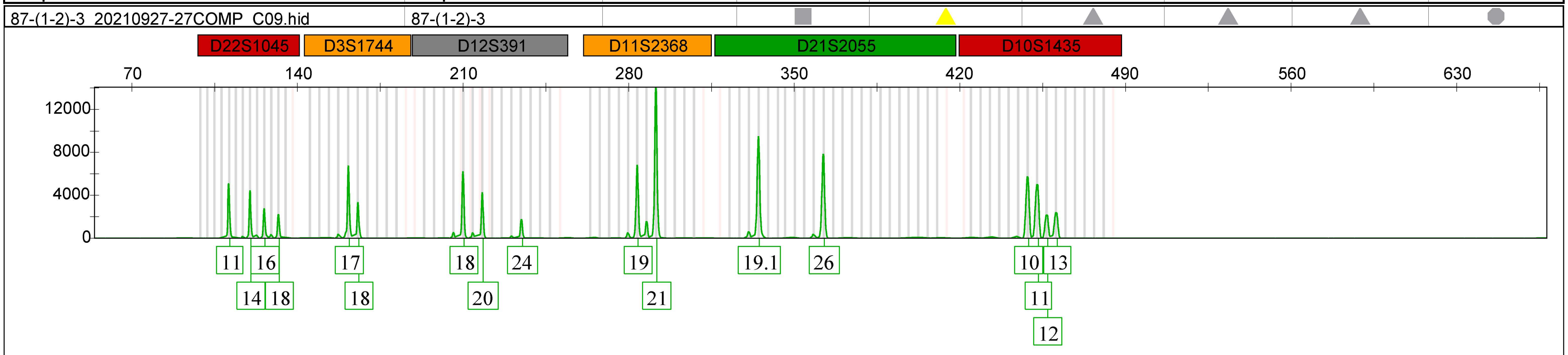

Sample File	Sample Name	SQO	SOS	SQ	SSPK	MIX	OMR	CGQ
87-(1-2)-3_20210927-27COMP_C09.hid	87-(1-2)-3							

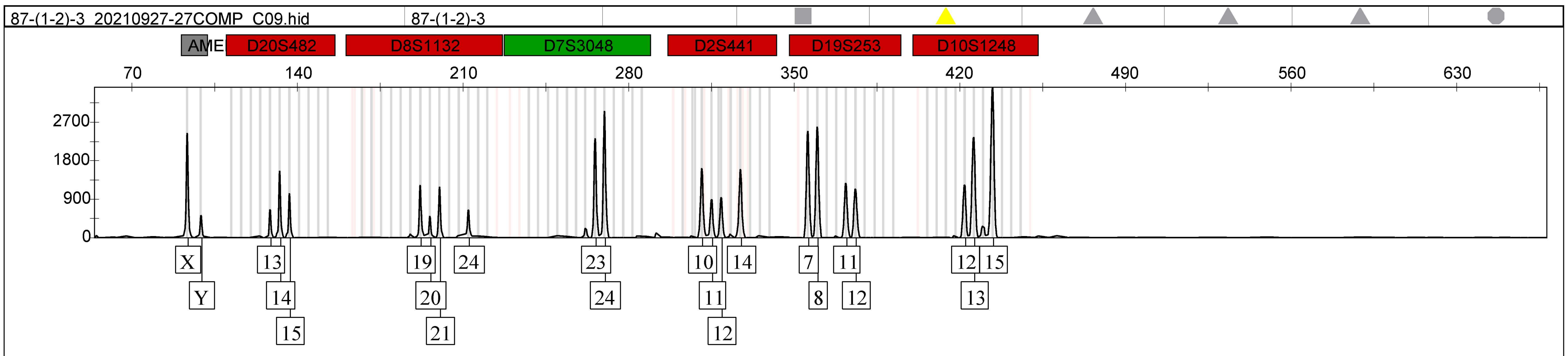

Sample File	Sample Name	SQO	SOS	SQ	SSPK	MIX	OMR	CGQ
87-(1-2)-3_20210927-27COMP_C09.hid	87-(1-2)-3							

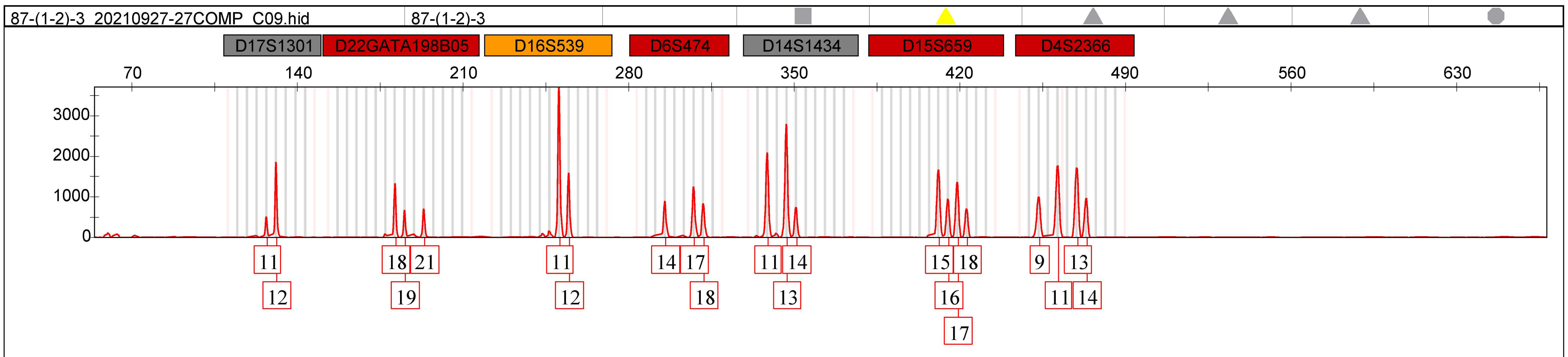

Project: 20210927-27COMP

GeneMapper® ID-X 1.5

Sample File	Sample Name	SQO	SOS	SQ	SSPK	MIX	OMR	CGQ
87-(1-4)-1_20210927-27COMP_D09.hid	87-(1-4)-1							

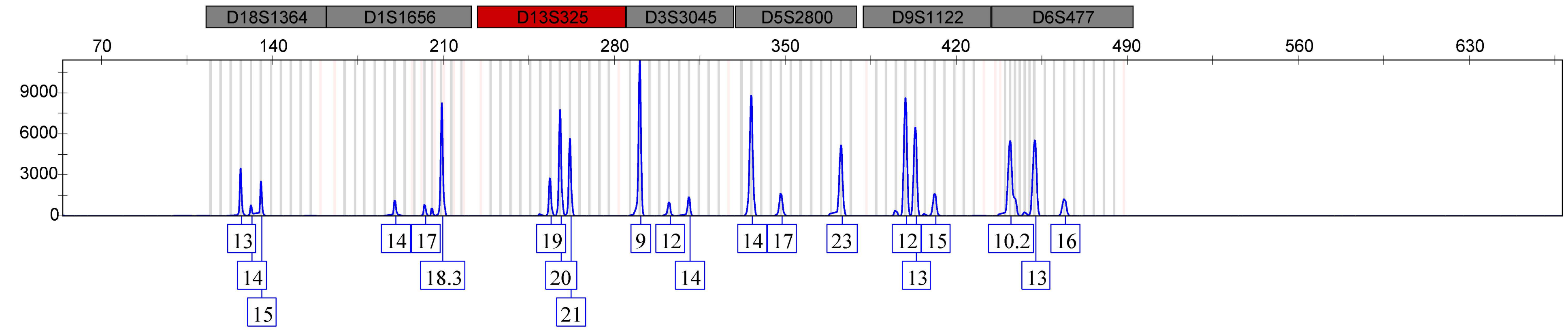

87-(1-4)-1_20210927-27COMP_D09.hid	87-(1-4)-1							

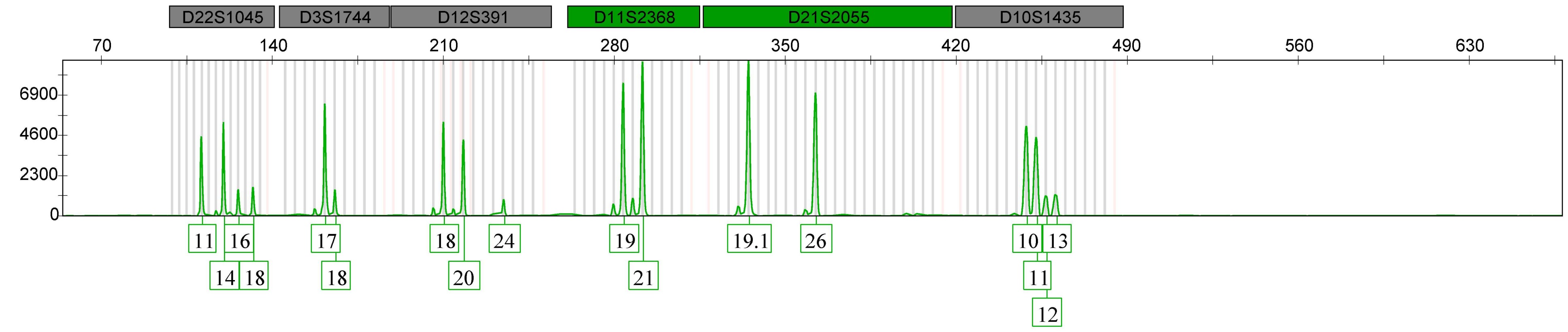

87-(1-4)-1_20210927-27COMP_D09.hid	87-(1-4)-1							

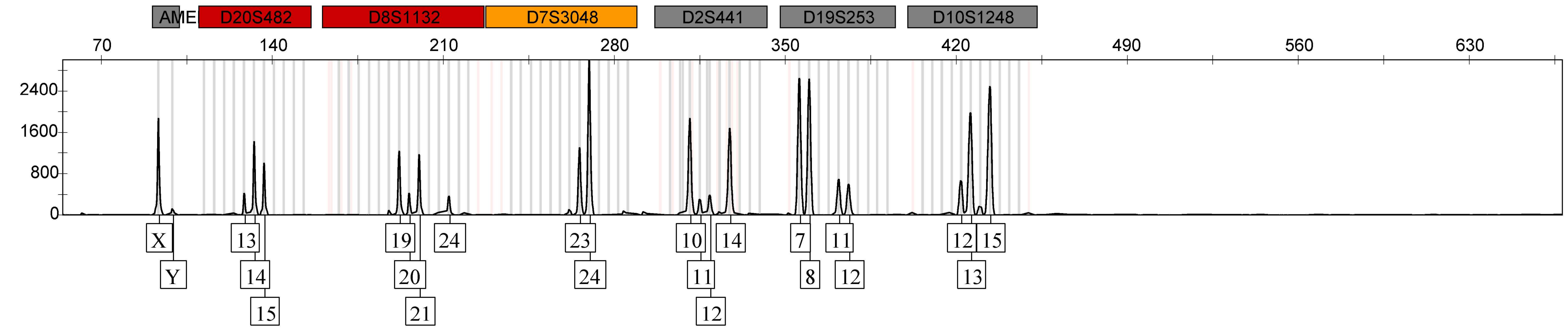

Project: 20210927-27COMP

GeneMapper® ID-X 1.5

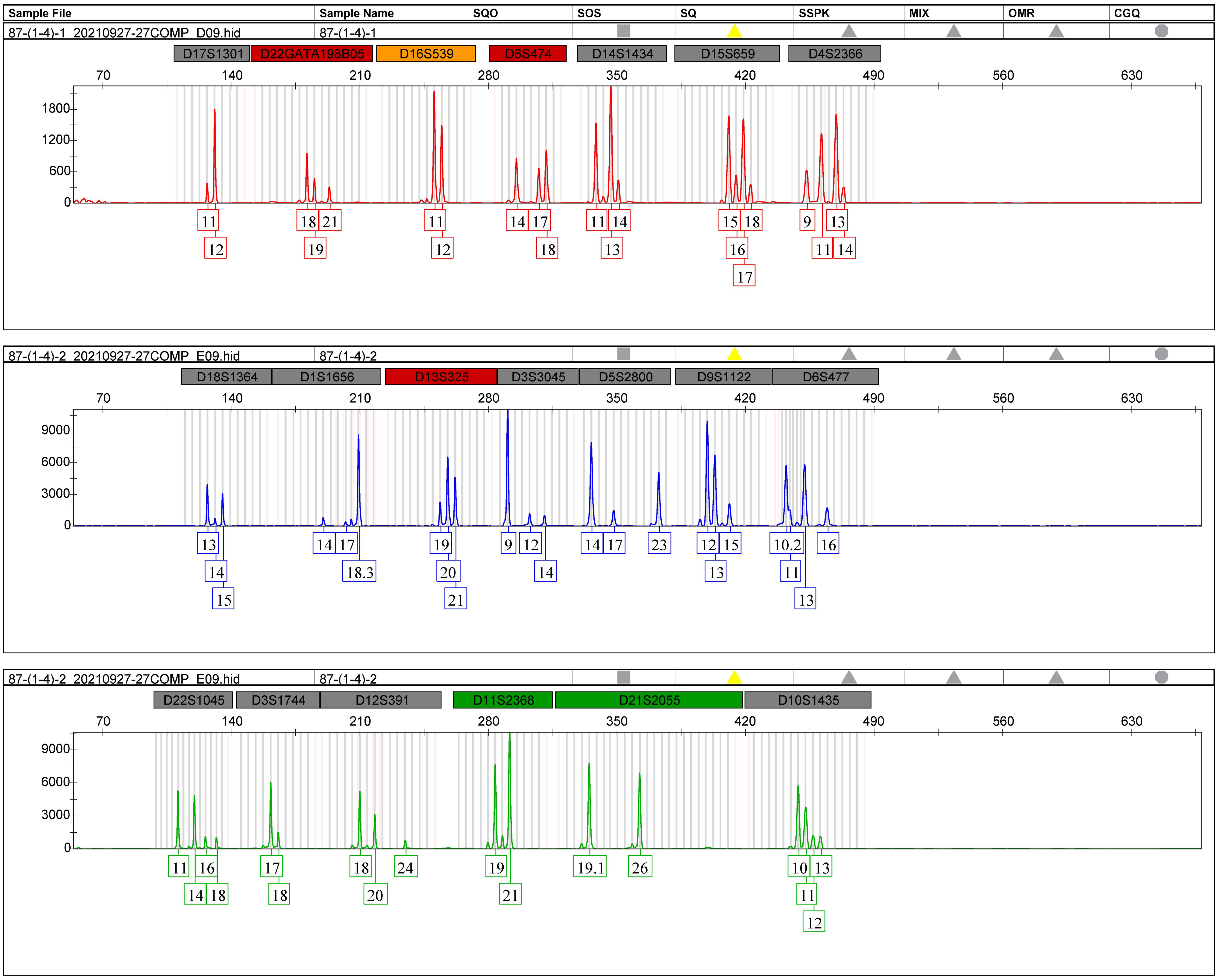

Project: 20210927-27COMP

GeneMapper® ID-X 1.5

Sample File	Sample Name	SQO	SOS	SQ	SSPK	MIX	OMR	CGQ
87-(1-4)-2_20210927-27COMP_E09.hid	87-(1-4)-2							

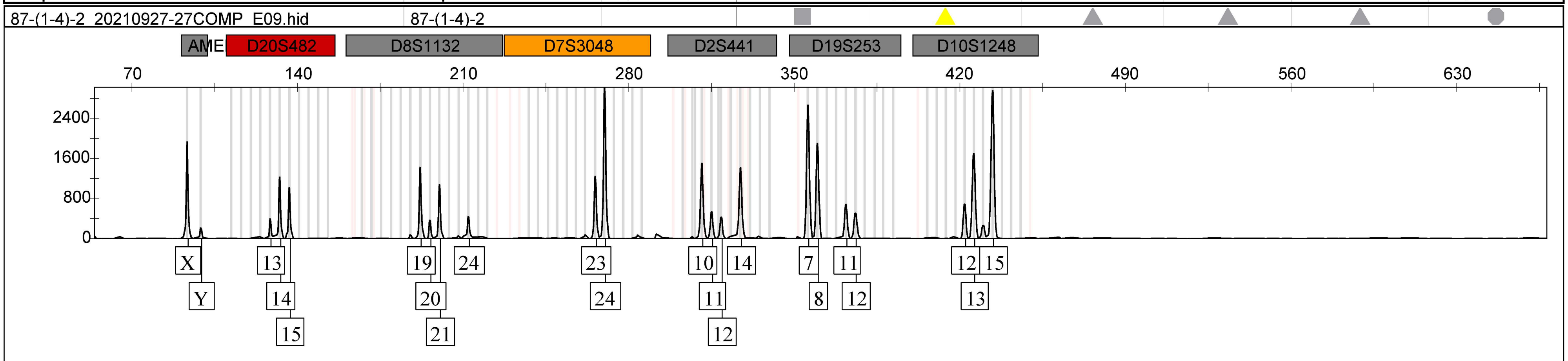

Sample File	Sample Name	SQO	SOS	SQ	SSPK	MIX	OMR	CGQ
87-(1-4)-2_20210927-27COMP_E09.hid	87-(1-4)-2							

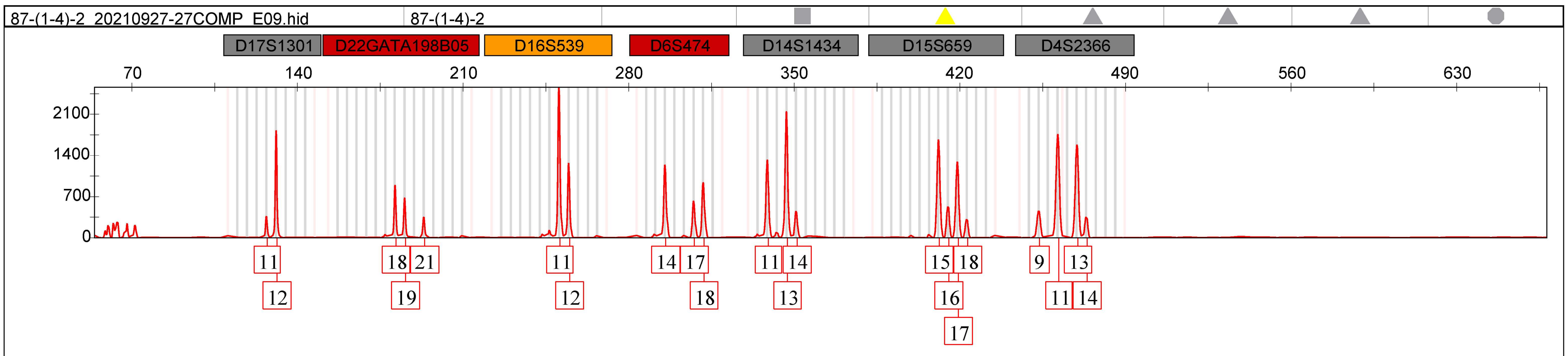

Sample File	Sample Name	SQO	SOS	SQ	SSPK	MIX	OMR	CGQ
87-(1-4)-3_20210927-27COMP_F09.hid	87-(1-4)-3							

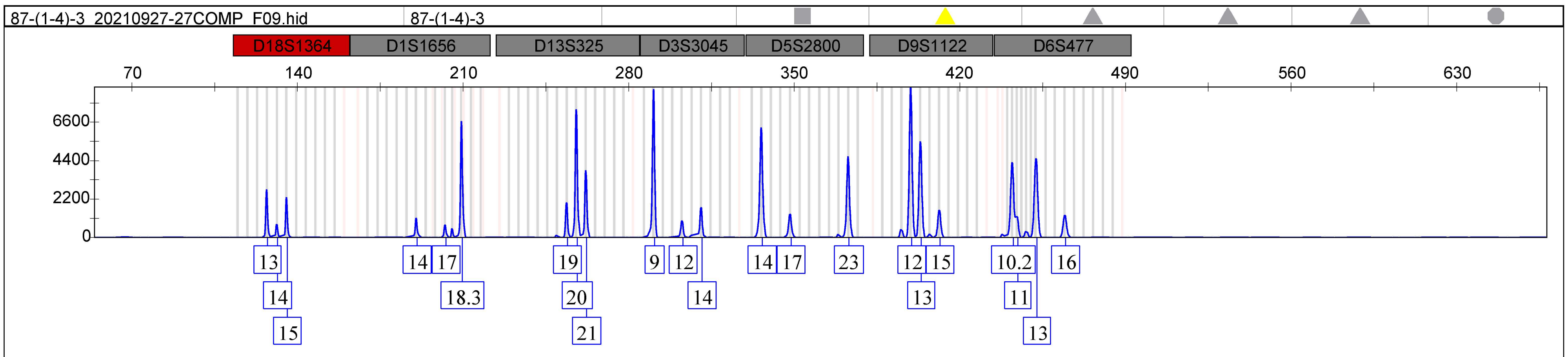

Project: 20210927-27COMP

GeneMapper® ID-X 1.5

Sample File	Sample Name	SQO	SOS	SQ	SSPK	MIX	OMR	CGQ
87-(1-4)-3_20210927-27COMP_F09.hid	87-(1-4)-3							

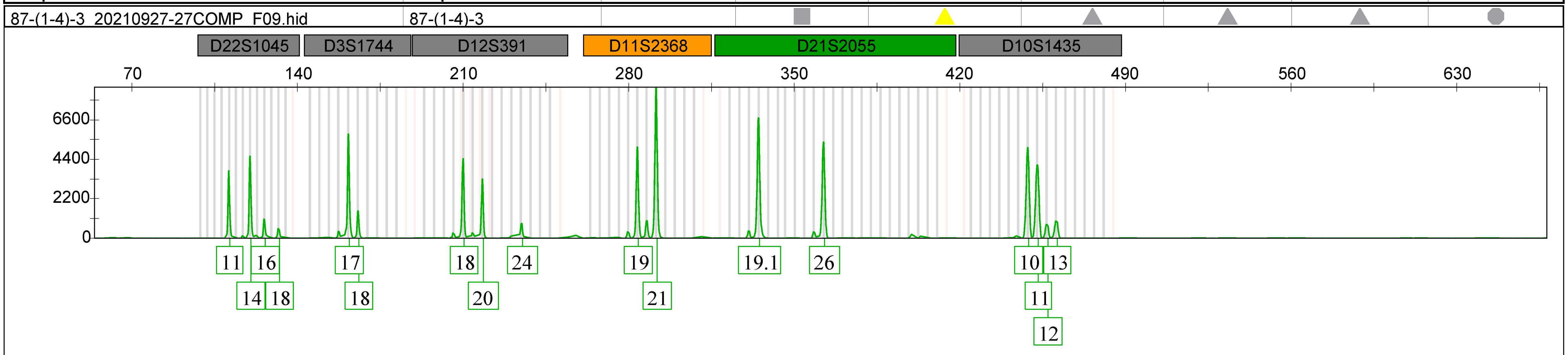

87-(1-4)-3_20210927-27COMP_F09.hid 87-(1-4)-3

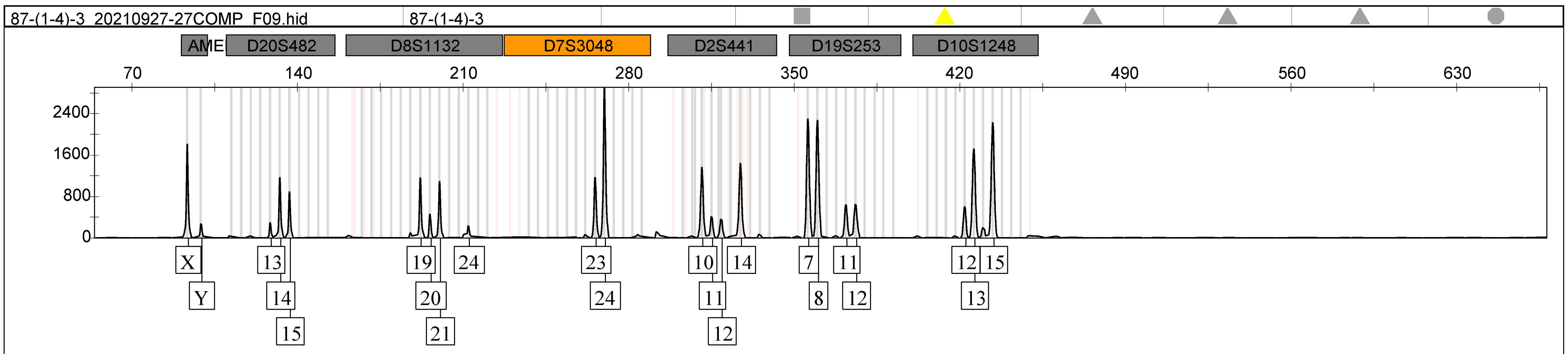

87-(1-4)-3_20210927-27COMP_F09.hid 87-(1-4)-3

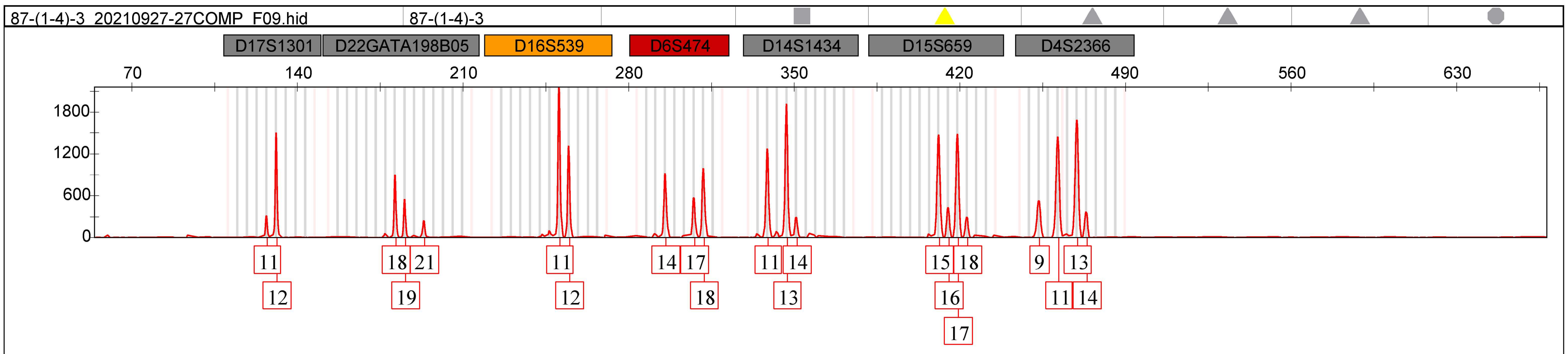

Sample File	Sample Name	SQO	SOS	SQ	SSPK	MIX	OMR	CGQ
87-(1-8)-1_20210927-27COMP_G09.hid	87-(1-8)-1							

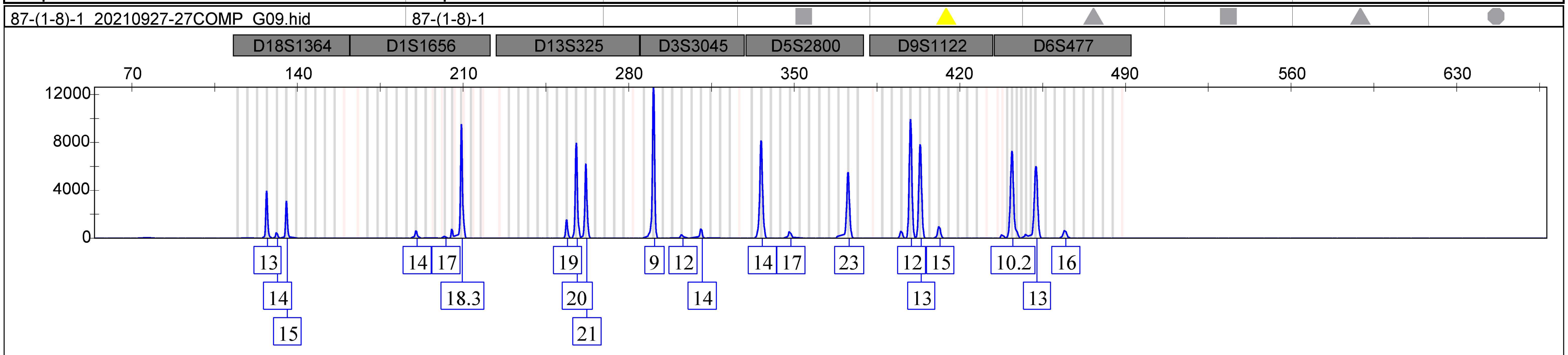

87-(1-8)-1_20210927-27COMP_G09.hid 87-(1-8)-1

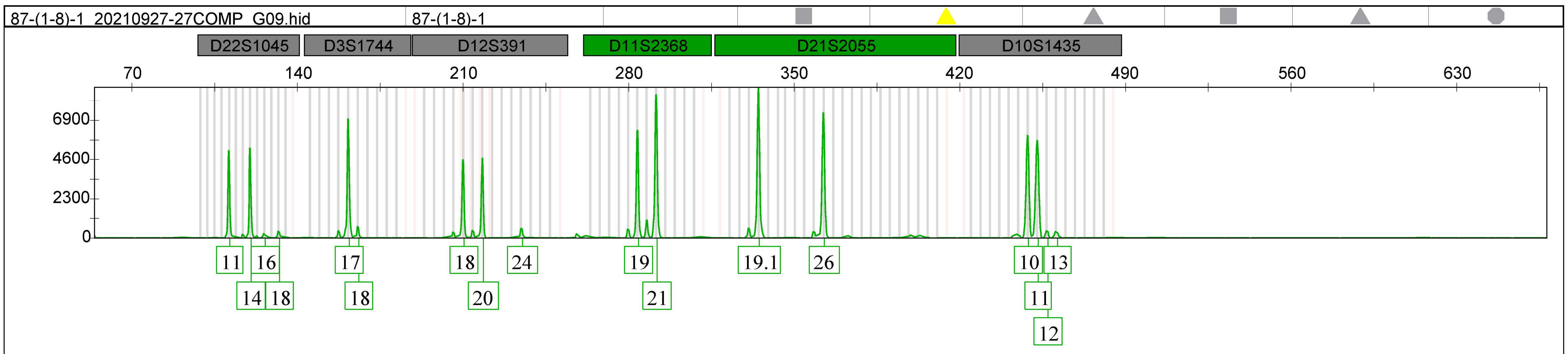

87-(1-8)-1_20210927-27COMP_G09.hid 87-(1-8)-1

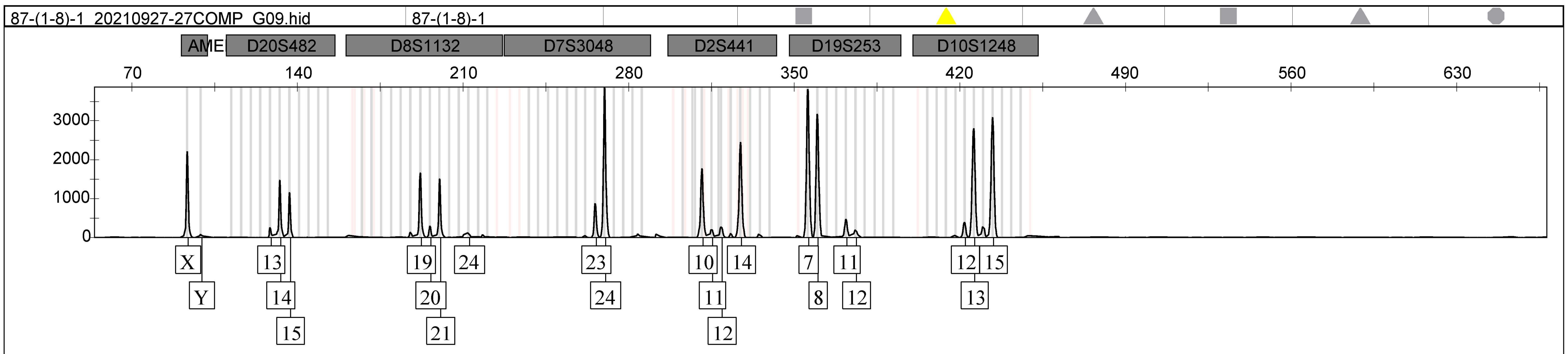

Project: 20210927-27COMP

GeneMapper® ID-X 1.5

Sample File	Sample Name	SQO	SOS	SQ	SSPK	MIX	OMR	CGQ
87-(1-8)-1_20210927-27COMP_G09.hid	87-(1-8)-1							

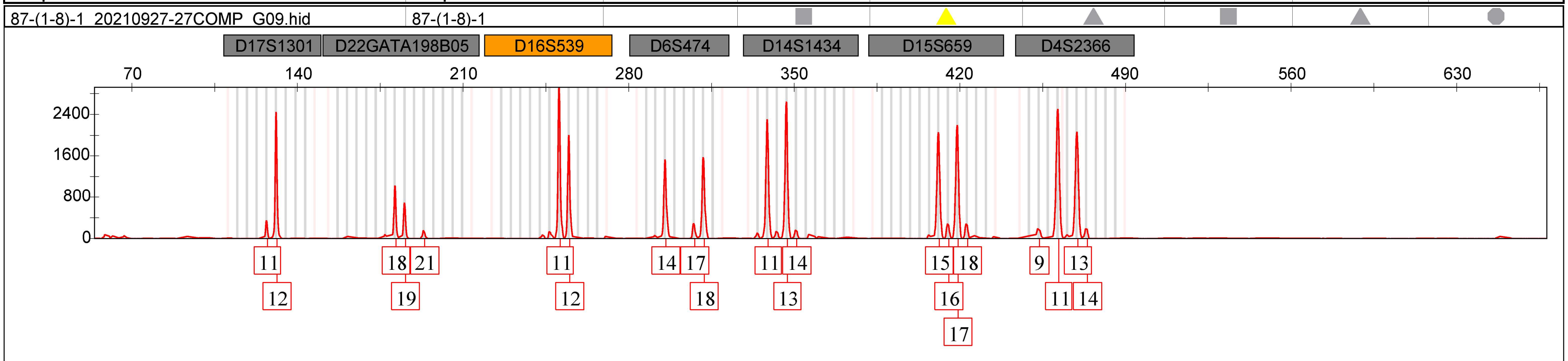

Sample File	Sample Name	SQO	SOS	SQ	SSPK	MIX	OMR	CGQ
87-(1-8)-2_20210927-27COMP_H09.hid	87-(1-8)-2							

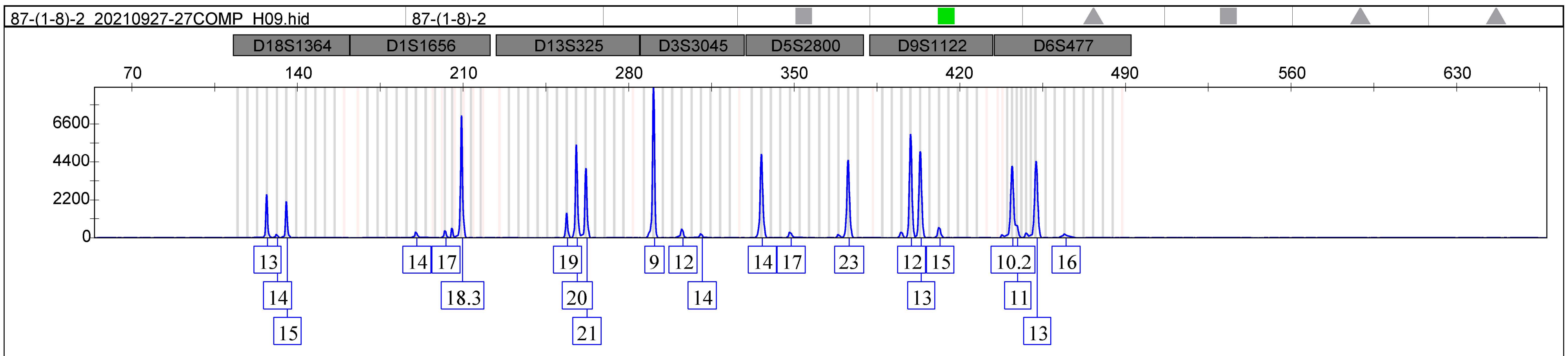

Sample File	Sample Name	SQO	SOS	SQ	SSPK	MIX	OMR	CGQ
87-(1-8)-2_20210927-27COMP_H09.hid	87-(1-8)-2							

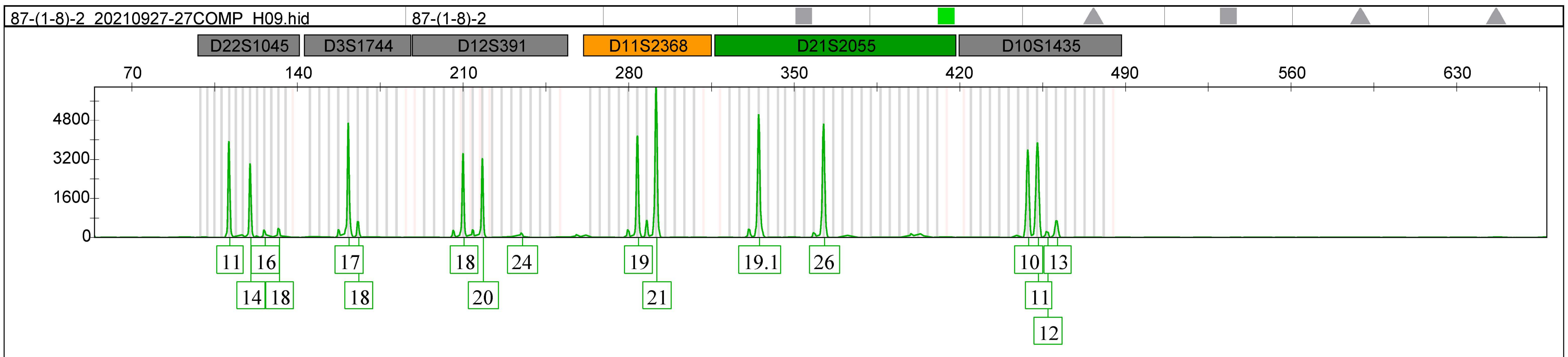

Sample File	Sample Name	SQO	SOS	SQ	SSPK	MIX	OMR	CGQ
87-(1-8)-2_20210927-27COMP_H09.hid	87-(1-8)-2							

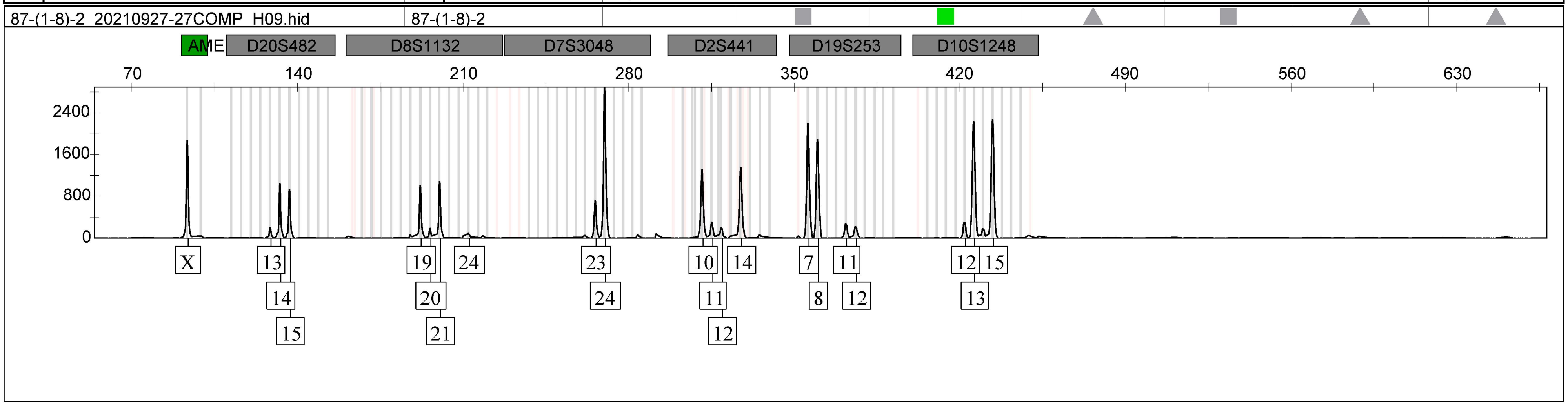

Sample File	Sample Name	SQO	SOS	SQ	SSPK	MIX	OMR	CGQ
87-(1-8)-2_20210927-27COMP_H09.hid	87-(1-8)-2							

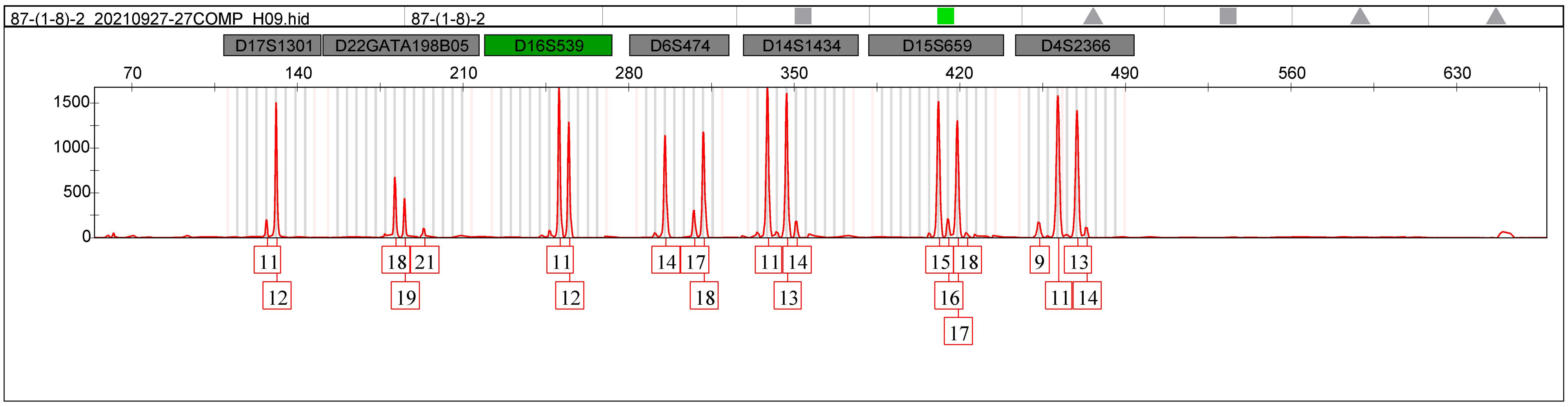

Sample File	Sample Name	SQO	SOS	SQ	SSPK	MIX	OMR	CGQ
87-(1-8)-3_20210927-27COMP_A10.hid	87-(1-8)-3							

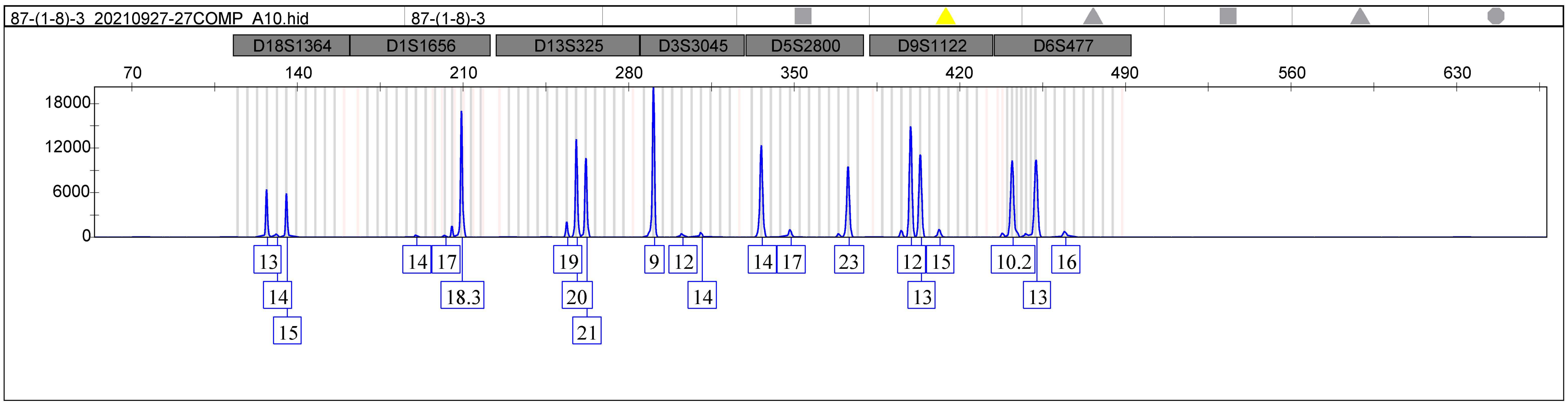

Project: 20210927-27COMP

GeneMapper® ID-X 1.5

Sample File	Sample Name	SQO	SOS	SQ	SSPK	MIX	OMR	CGQ
87-(1-8)-3_20210927-27COMP_A10.hid	87-(1-8)-3							

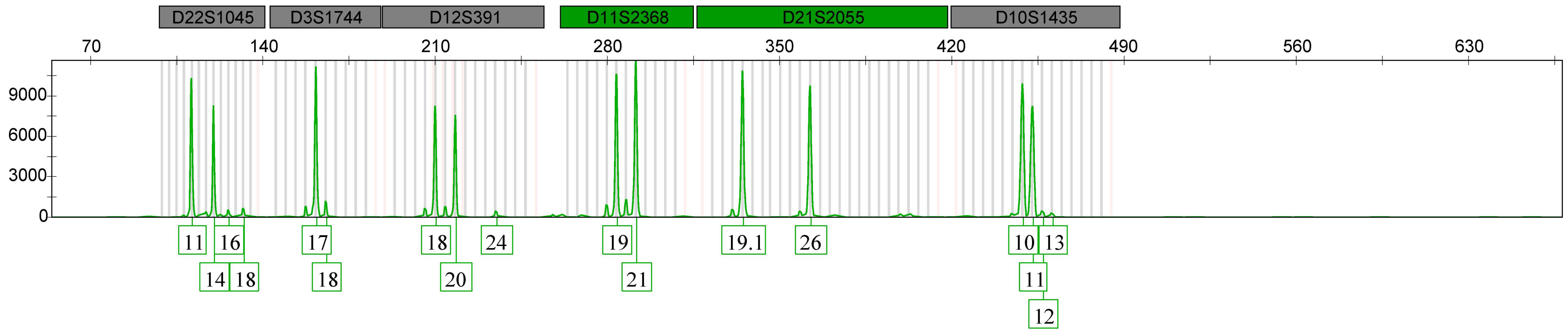

87-(1-8)-3_20210927-27COMP_A10.hid 87-(1-8)-3

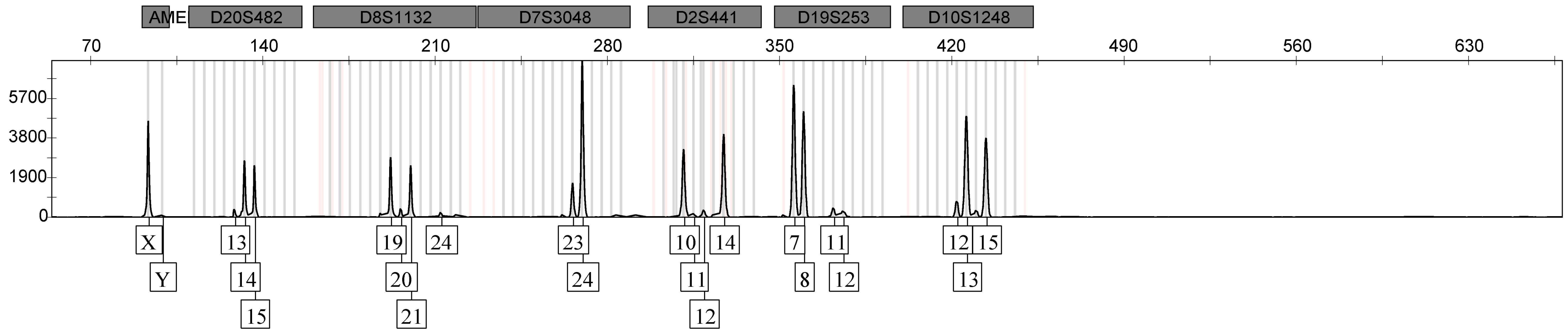

87-(1-8)-3_20210927-27COMP_A10.hid 87-(1-8)-3

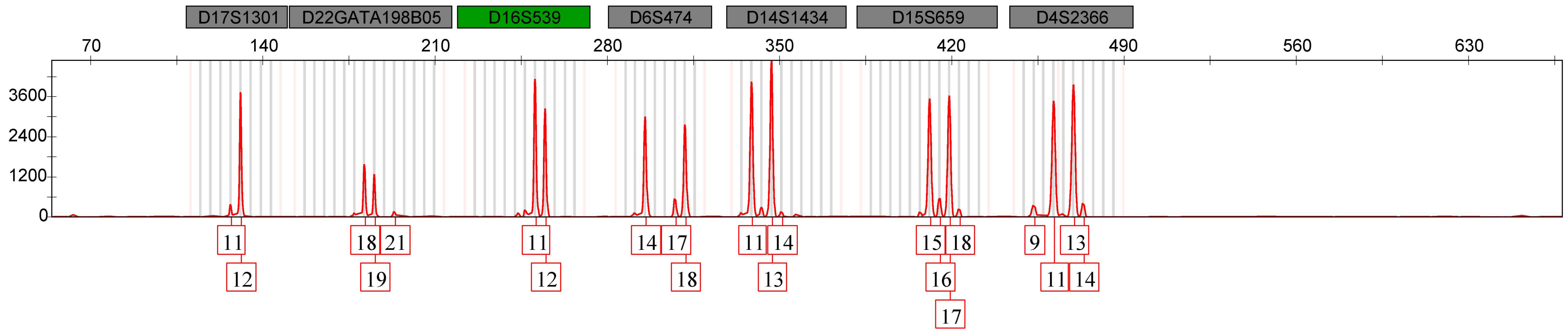

GeneMapper® ID-X 1.5

Sample File	Sample Name	SQO	SOS	SQ	SSPK	MIX	OMR	CGQ
87-(1-19)-1_20210927-27COMP_B10.hid	87-(1-19)-1							

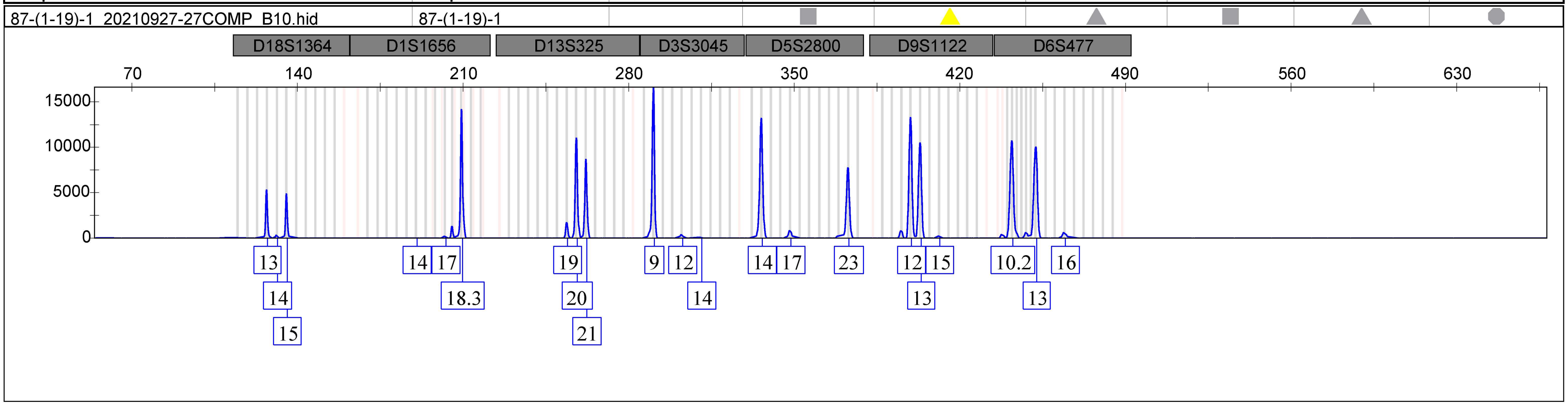

Sample File	Sample Name
87-(1-19)-1_20210927-27COMP_B10.hid	87-(1-19)-1

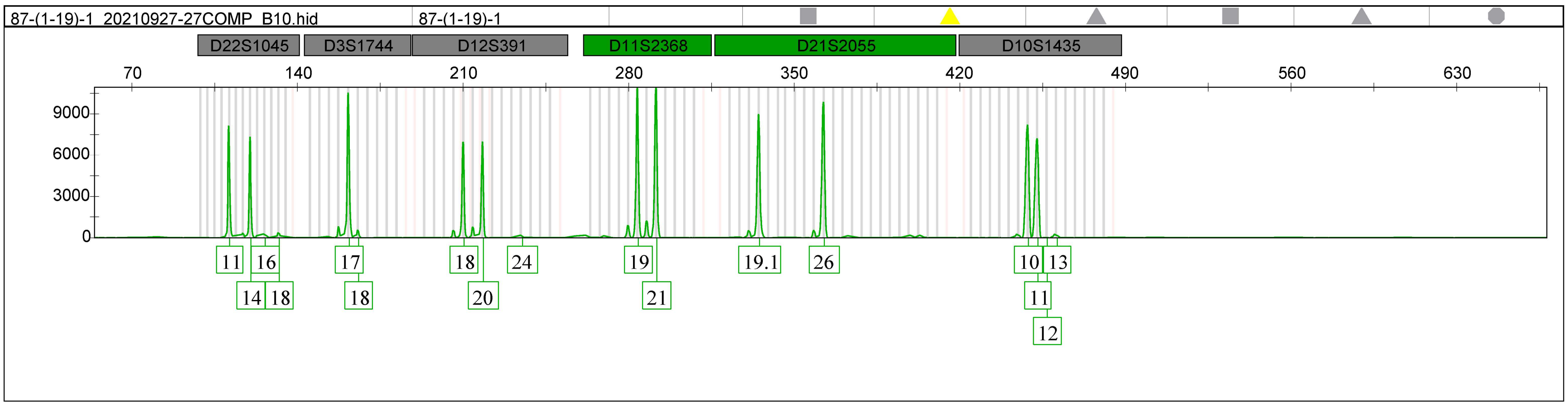

Sample File	Sample Name
87-(1-19)-1_20210927-27COMP_B10.hid	87-(1-19)-1

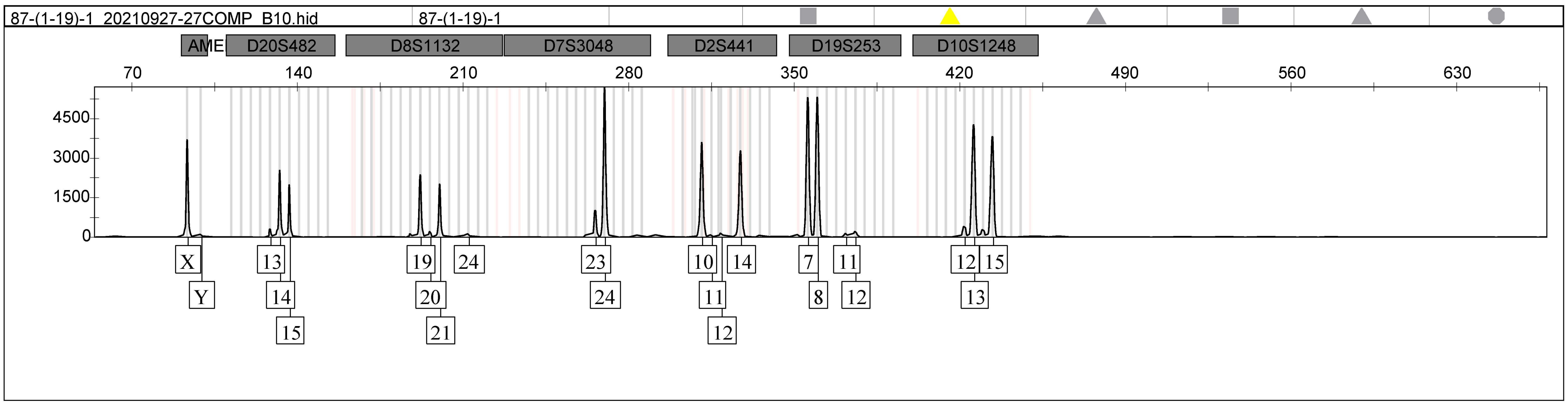

GeneMapper® ID-X 1.5

Sample File	Sample Name	SQO	SOS	SQ	SSPK	MIX	OMR	CGQ
87-(1-19)-1_20210927-27COMP_B10.hid	87-(1-19)-1							

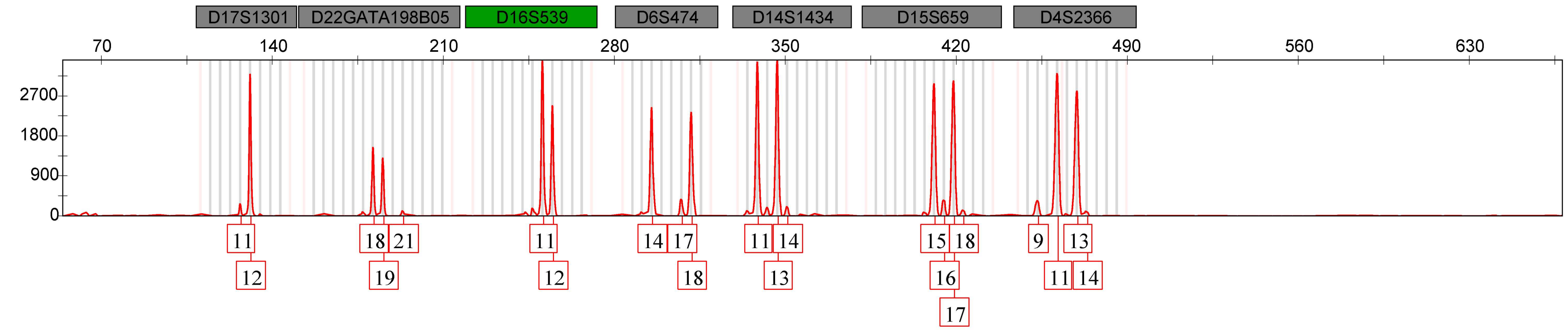

Sample File	Sample Name	SQO	SOS	SQ	SSPK	MIX	OMR	CGQ
87-(1-19)-2_20210927-27COMP_C10.hid	87-(1-19)-2							

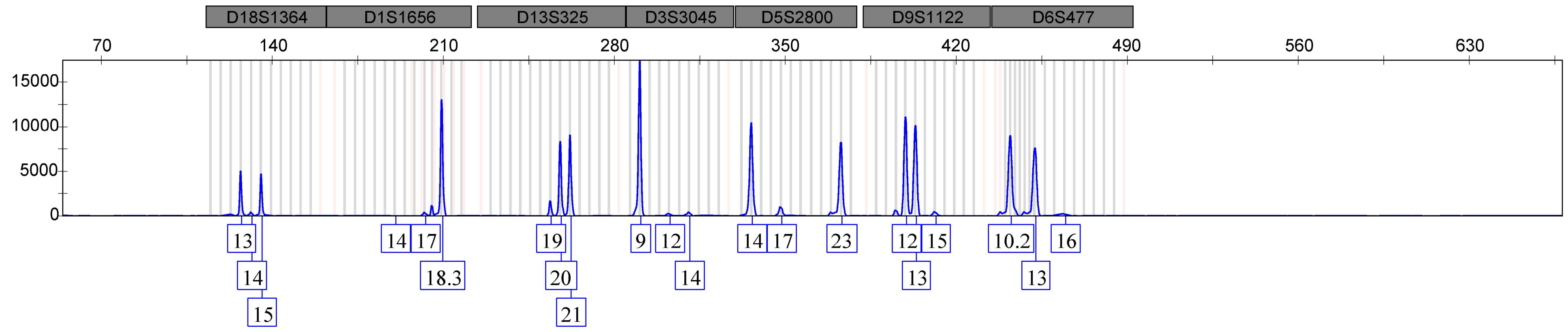

Sample File	Sample Name	SQO	SOS	SQ	SSPK	MIX	OMR	CGQ
87-(1-19)-2_20210927-27COMP_C10.hid	87-(1-19)-2							

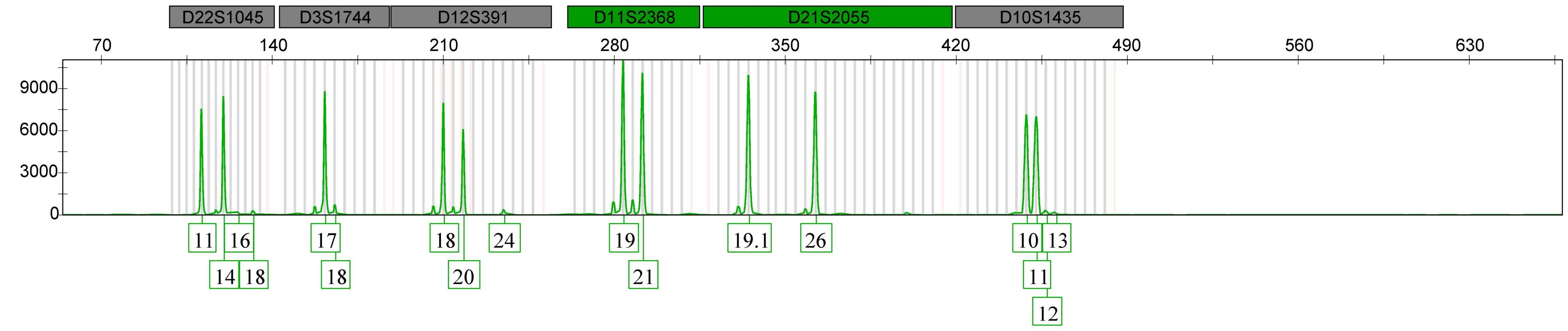

Project: 20210927-27COMP

GeneMapper® ID-X 1.5

Sample File	Sample Name	SQO	SOS	SQ	SSPK	MIX	OMR	CGQ
87-(1-19)-2_20210927-27COMP_C10.hid	87-(1-19)-2							

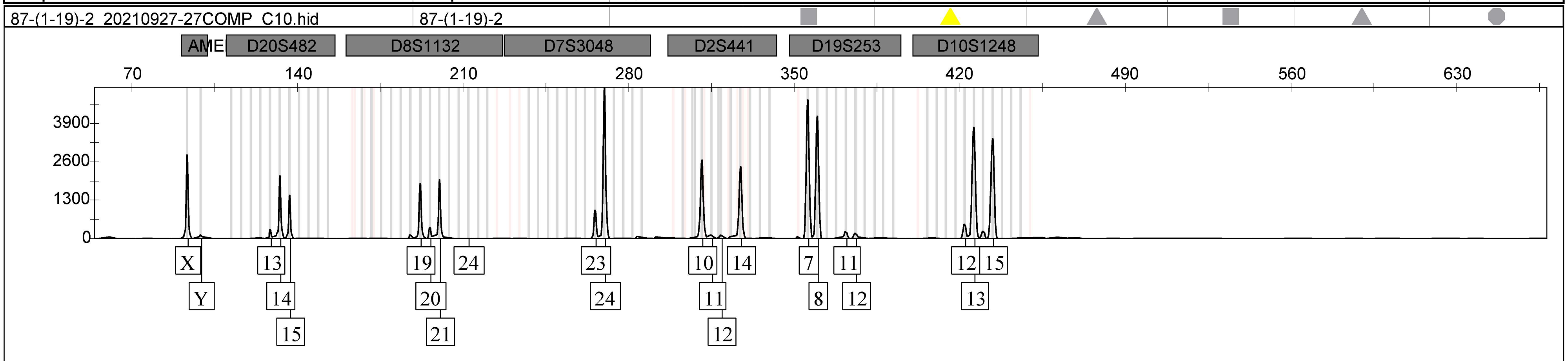

87-(1-19)-2_20210927-27COMP_C10.hid	87-(1-19)-2							

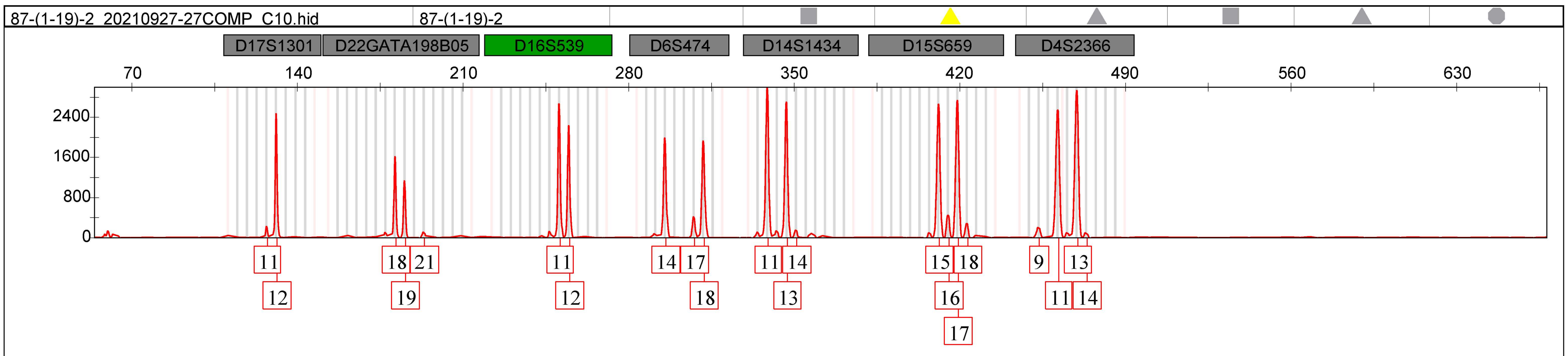

87-(1-19)-3_20210927-27COMP_D10.hid	87-(1-19)-3							

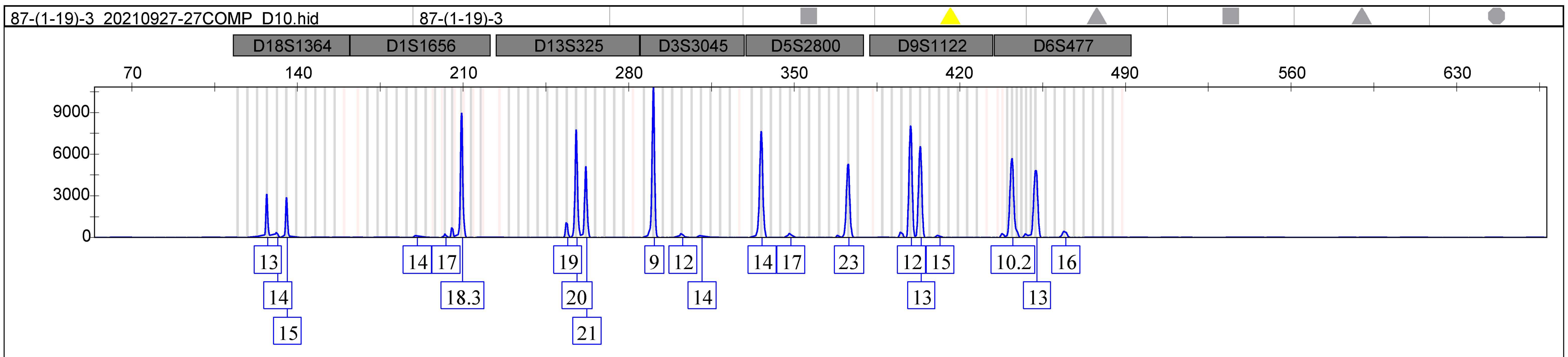

Project: 20210927-27COMP

GeneMapper® ID-X 1.5

Sample File	Sample Name	SQO	SOS	SQ	SSPK	MIX	OMR	CGQ
87-(1-19)-3_20210927-27COMP_D10.hid	87-(1-19)-3							

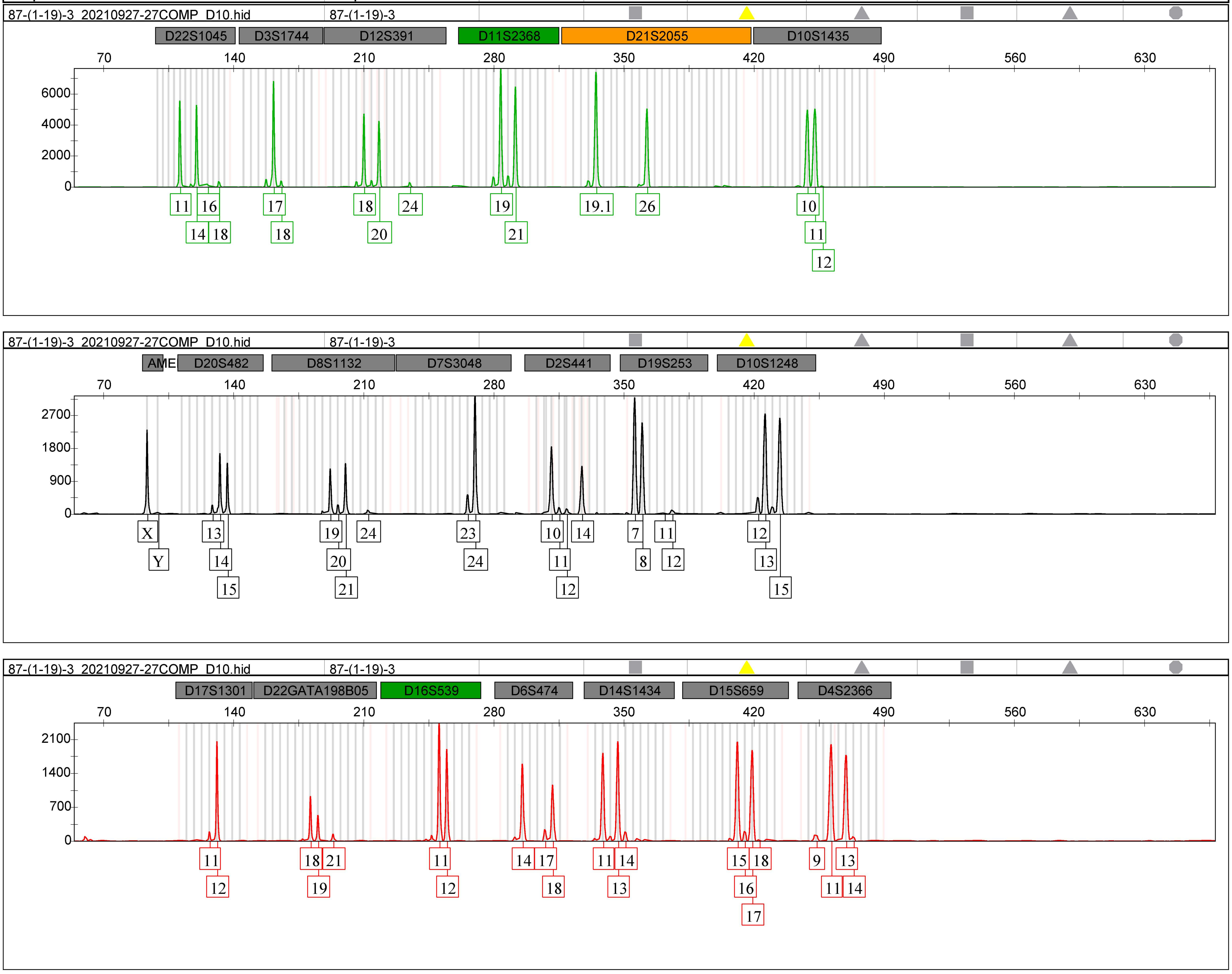

Sample File	Sample Name	SQO	SOS	SQ	SSPK	MIX	OMR	CGQ
87-(2-1)-1_20210927-27COMP_C08.hid	87-(2-1)-1							

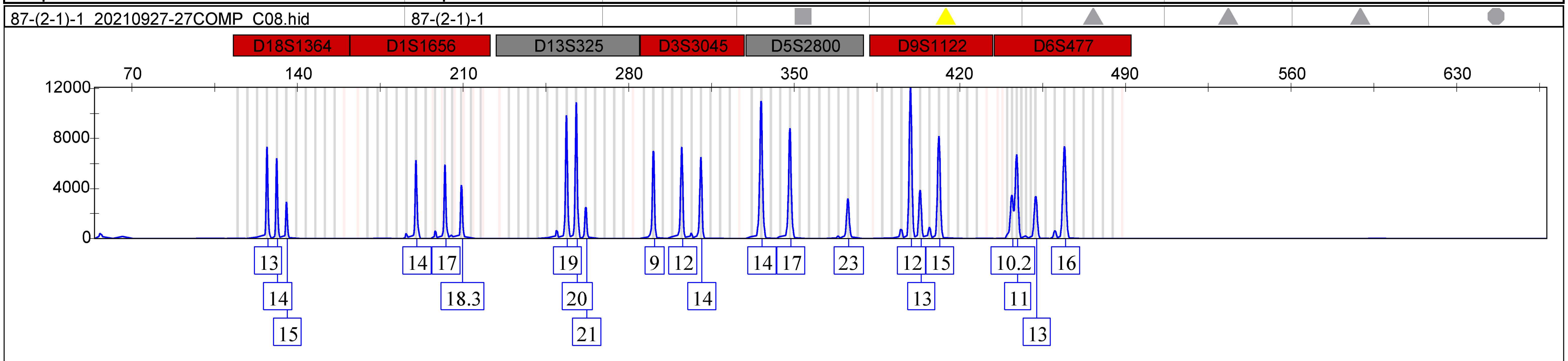

87-(2-1)-1_20210927-27COMP_C08.hid 87-(2-1)-1

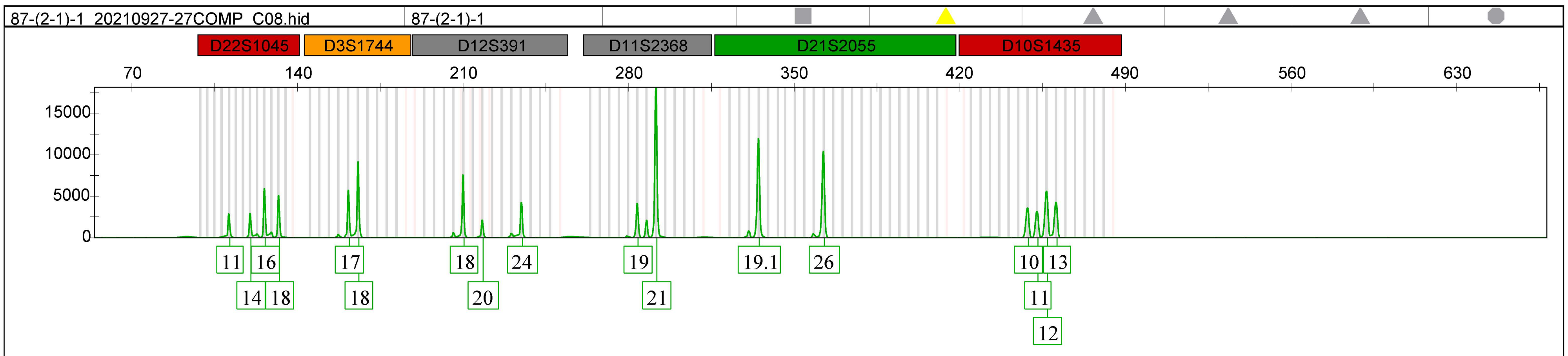

87-(2-1)-1_20210927-27COMP_C08.hid 87-(2-1)-1

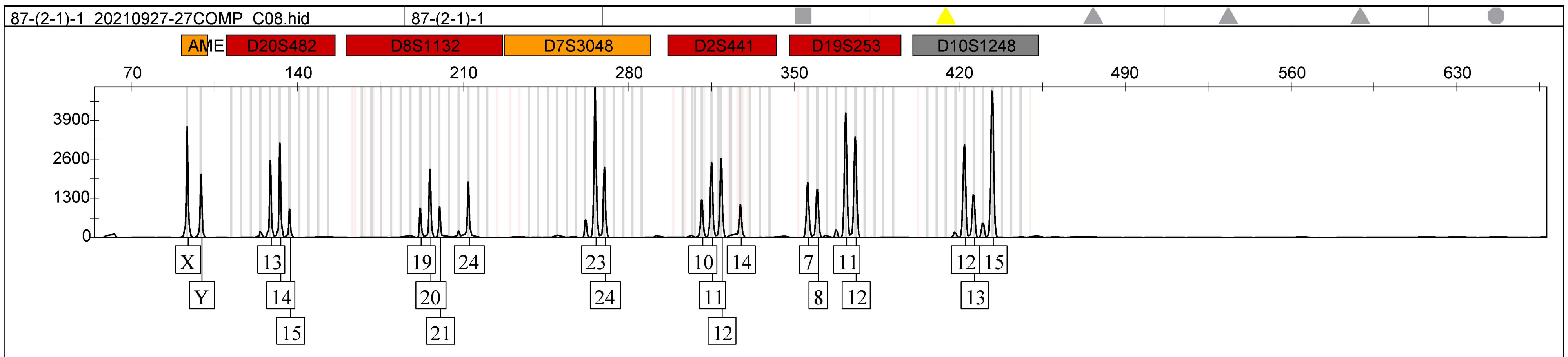

Project: 20210927-27COMP

GeneMapper® ID-X 1.5

Sample File	Sample Name	SQO	SOS	SQ	SSPK	MIX	OMR	CGQ
87-(2-1)-1_20210927-27COMP_C08.hid	87-(2-1)-1							

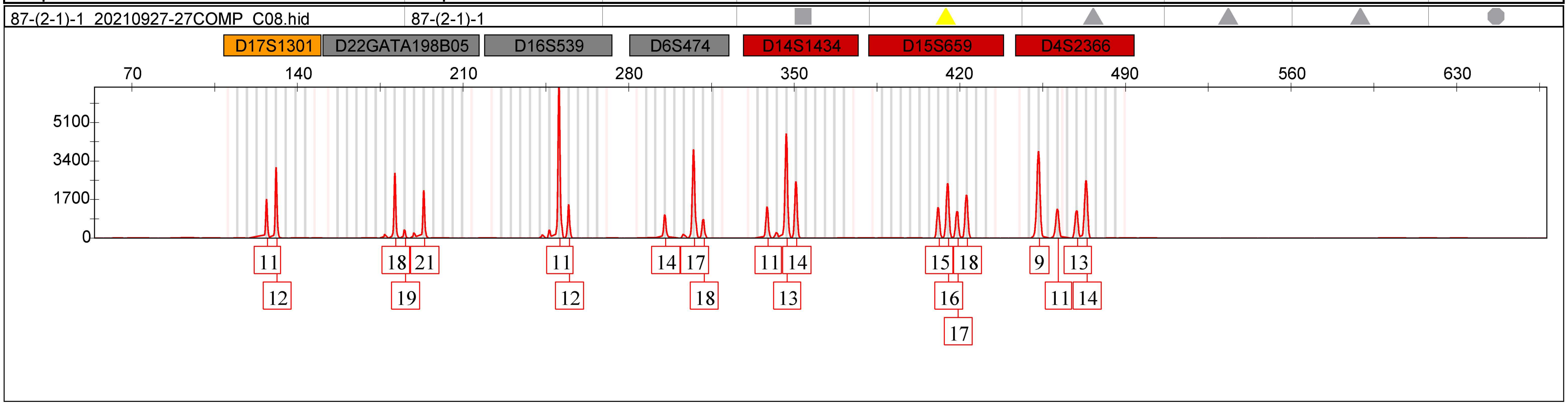

Sample File	Sample Name	SQO	SOS	SQ	SSPK	MIX	OMR	CGQ
87-(2-1)-2_20210927-27COMP_D08.hid	87-(2-1)-2							

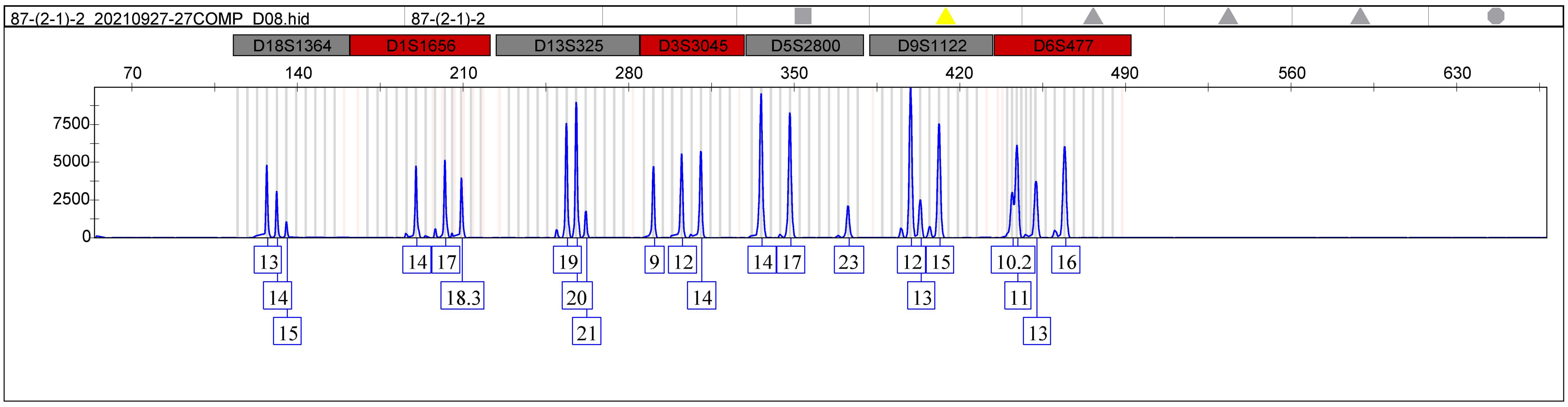

Sample File	Sample Name	SQO	SOS	SQ	SSPK	MIX	OMR	CGQ
87-(2-1)-2_20210927-27COMP_D08.hid	87-(2-1)-2							

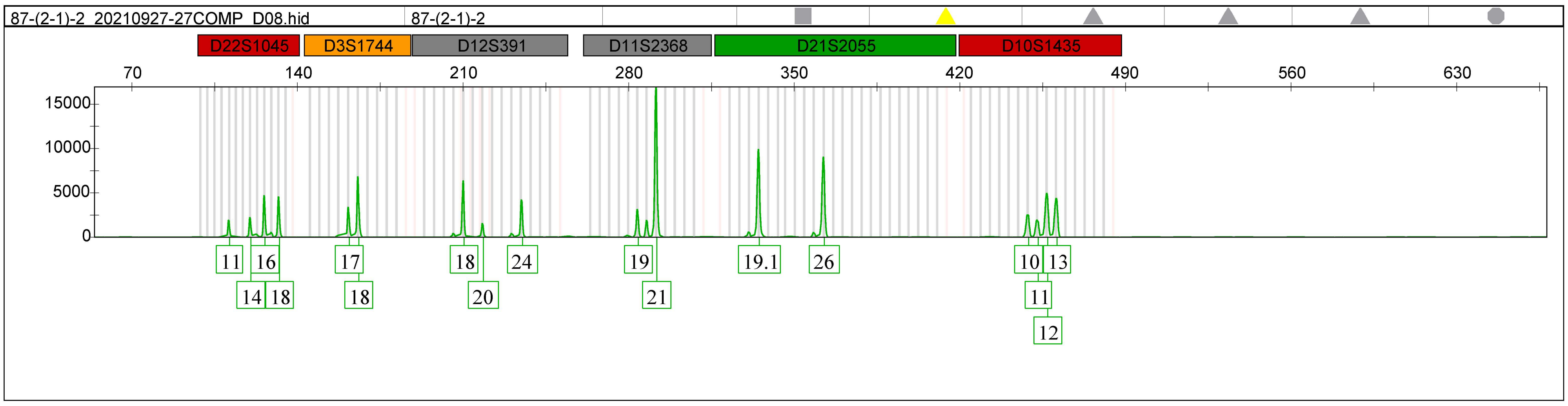

Project: 20210927-27COMP

GeneMapper® ID-X 1.5

Sample File	Sample Name	SQO	SOS	SQ	SSPK	MIX	OMR	CGQ
87-(2-1)-2_20210927-27COMP_D08.hid	87-(2-1)-2							

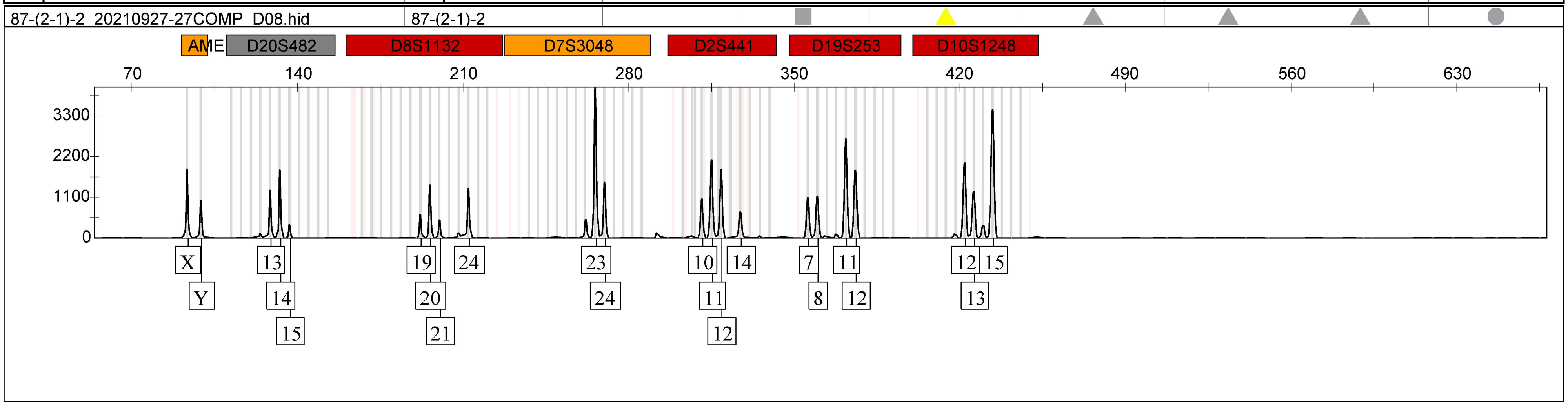

Sample File	Sample Name
87-(2-1)-2_20210927-27COMP_D08.hid	87-(2-1)-2

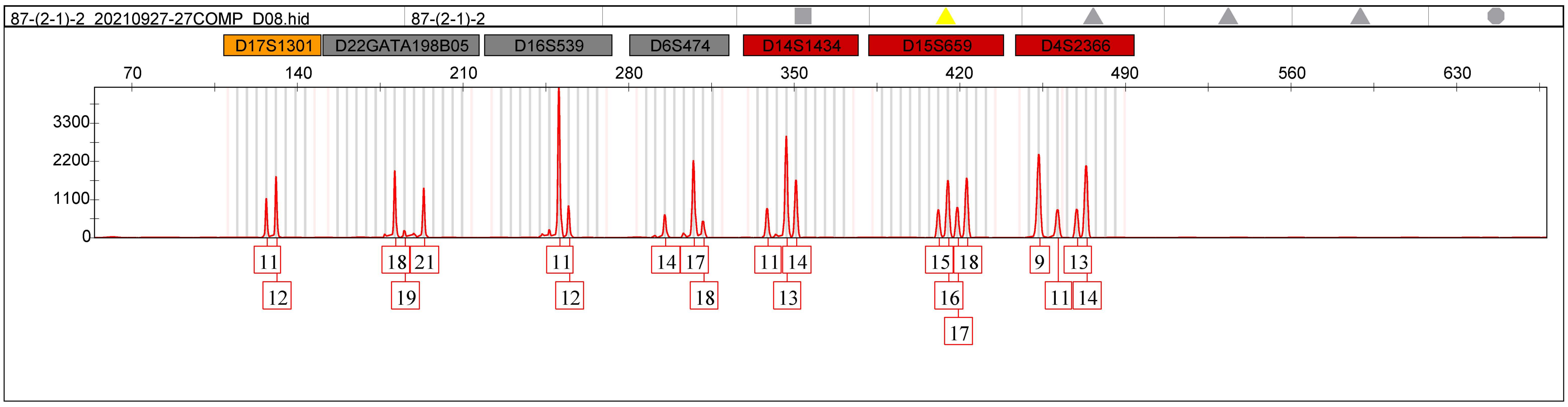

Sample File	Sample Name
87-(2-1)-3_20210927-27COMP_E08.hid	87-(2-1)-3

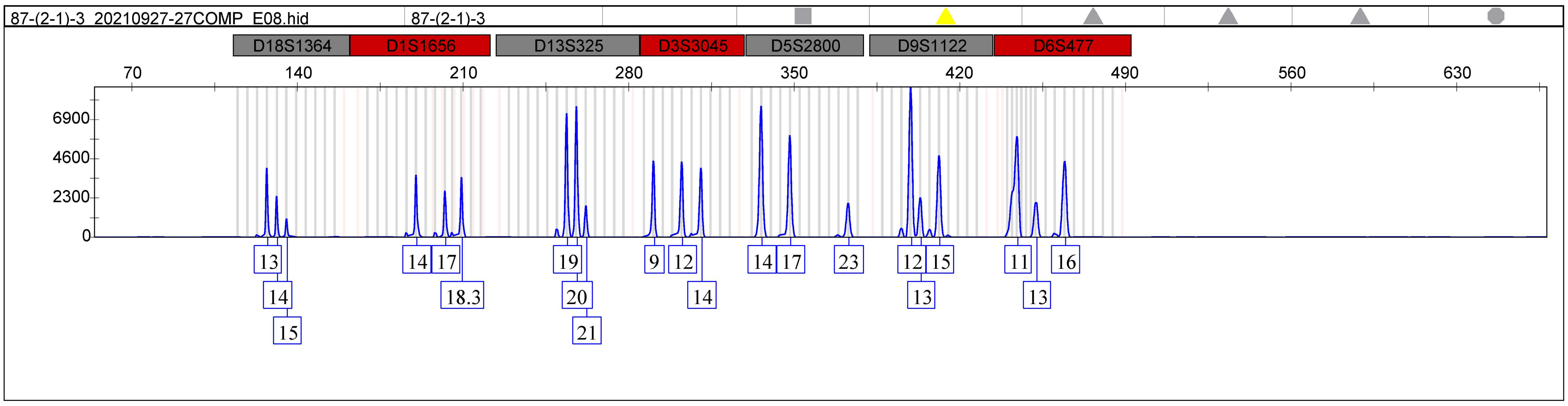

Project: 20210927-27COMP

GeneMapper® ID-X 1.5

Sample File	Sample Name	SQO	SOS	SQ	SSPK	MIX	OMR	CGQ
87-(2-1)-3_20210927-27COMP_E08.hid	87-(2-1)-3							

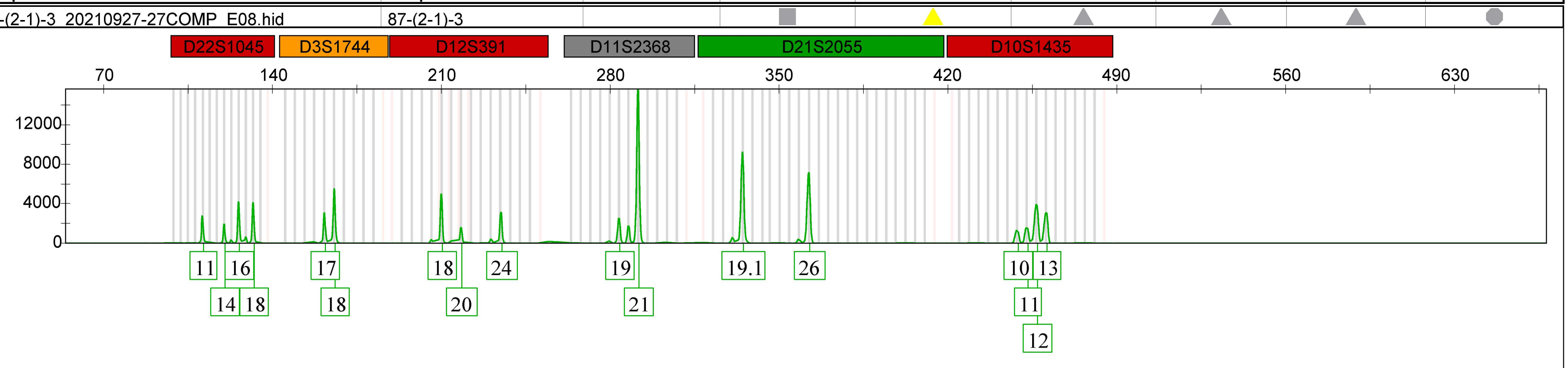

87-(2-1)-3_20210927-27COMP_E08.hid 87-(2-1)-3

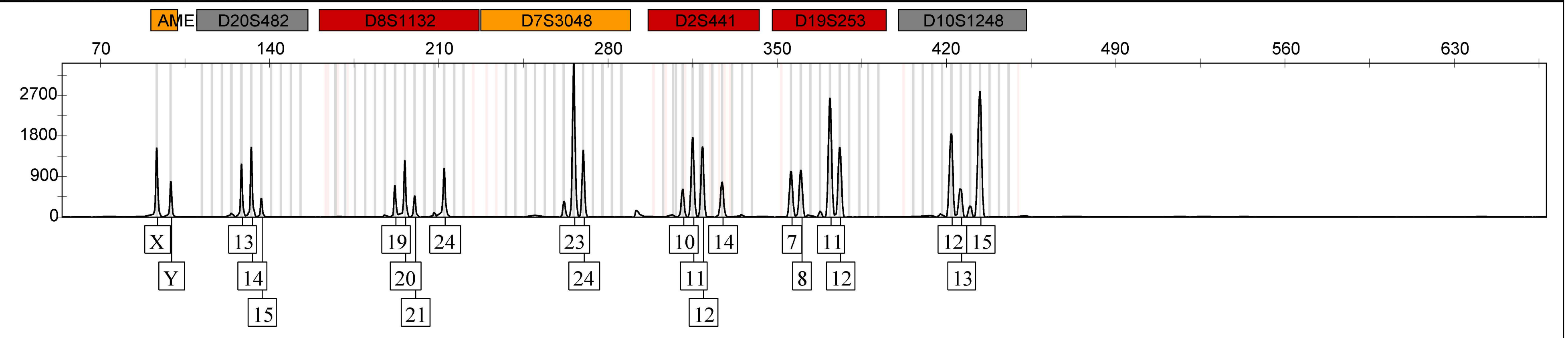

87-(2-1)-3_20210927-27COMP_E08.hid 87-(2-1)-3

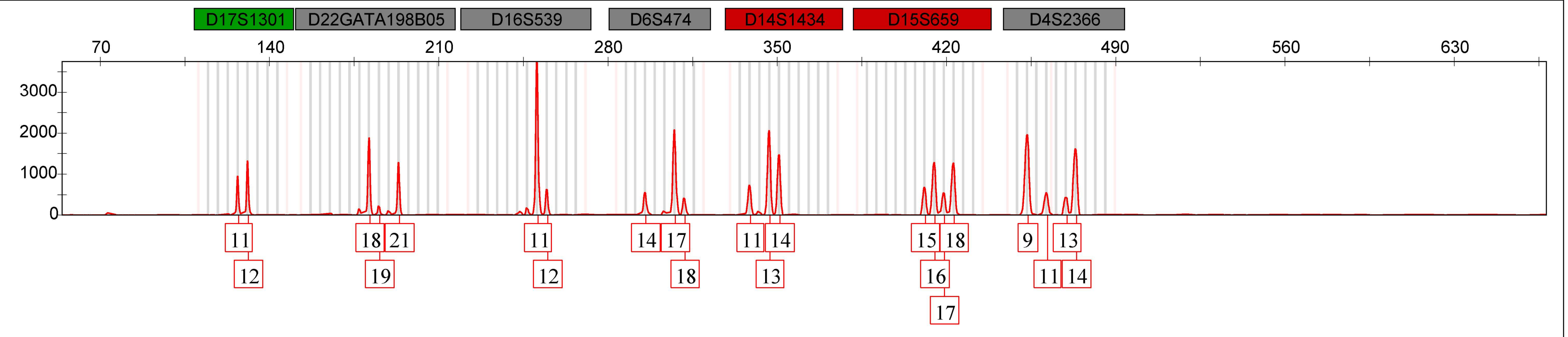

Project: 20210927-27COMP

GeneMapper® ID-X 1.5

Sample File	Sample Name	SQO	SOS	SQ	SSPK	MIX	OMR	CGQ
87-(4-1)-1_20210927-27COMP_H07.hid	87-(4-1)-1							

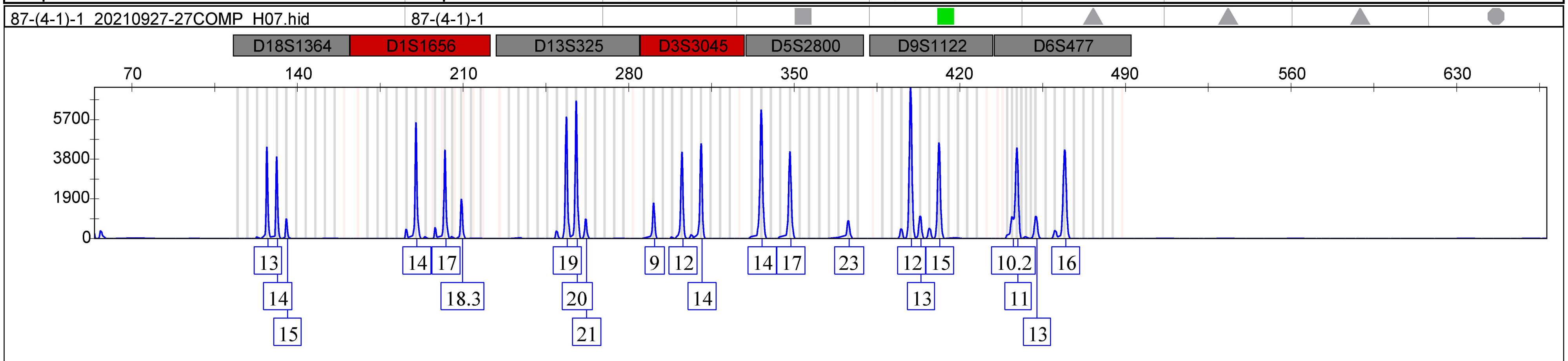

87-(4-1)-1_20210927-27COMP_H07.hid 87-(4-1)-1

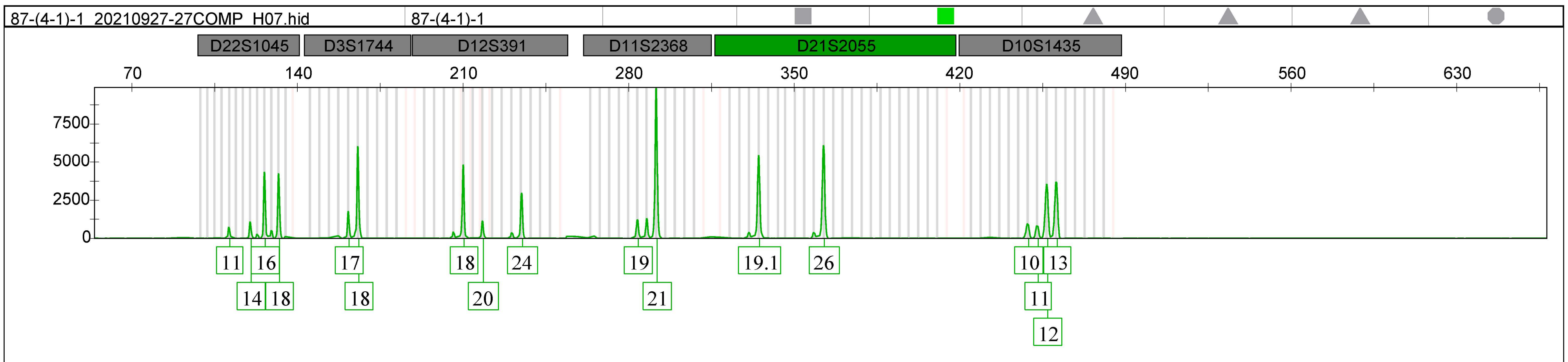

87-(4-1)-1_20210927-27COMP_H07.hid 87-(4-1)-1

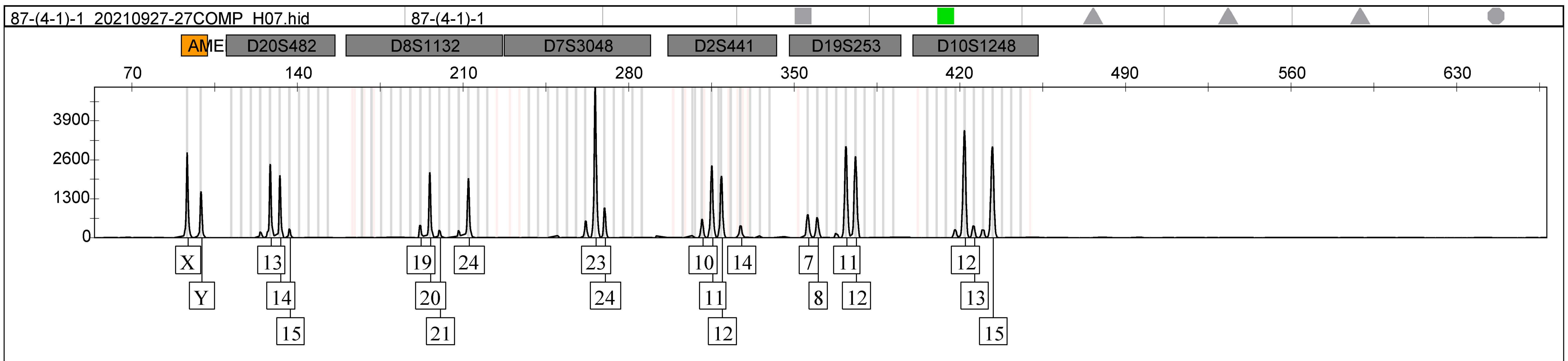

Project: 20210927-27COMP

GeneMapper® ID-X 1.5

Sample File	Sample Name	SQO	SOS	SQ	SSPK	MIX	OMR	CGQ
87-(4-1)-1_20210927-27COMP_H07.hid	87-(4-1)-1							

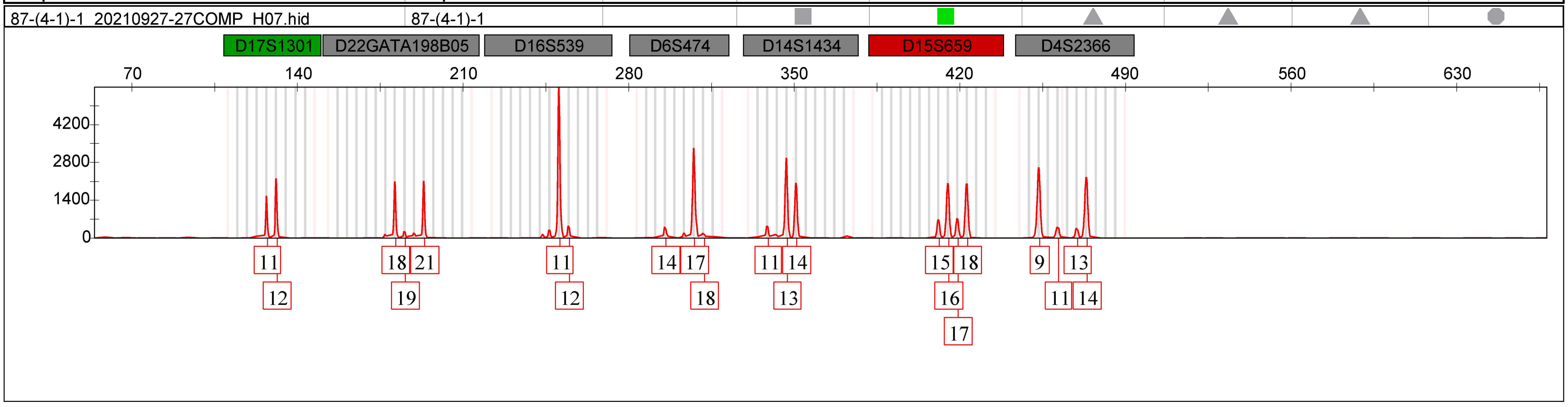

Sample File	Sample Name	SQO	SOS	SQ	SSPK	MIX	OMR	CGQ
87-(4-1)-2_20210927-27COMP_A08.hid	87-(4-1)-2							

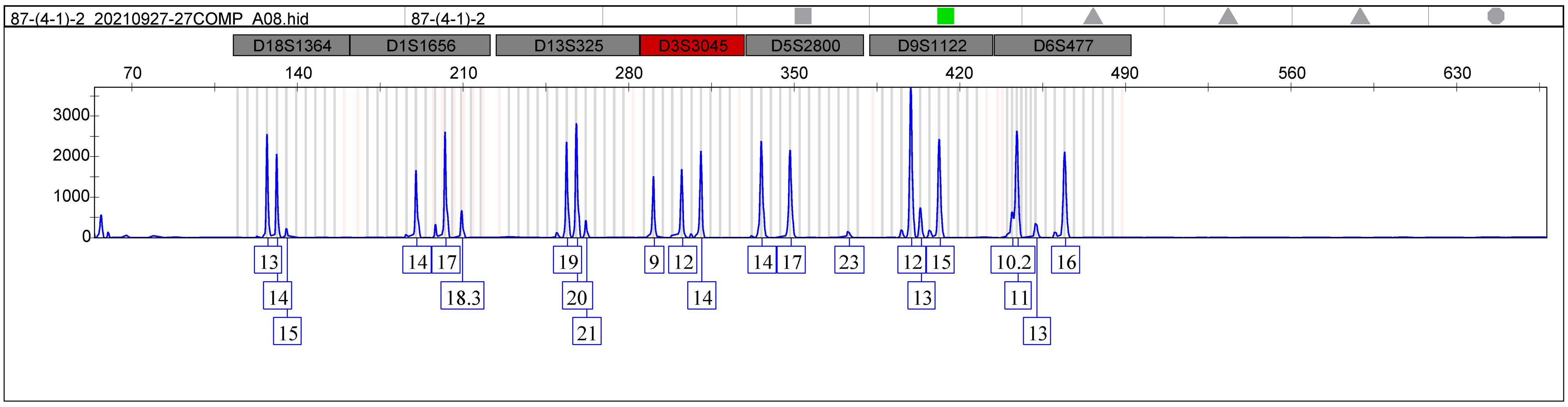

Sample File	Sample Name	SQO	SOS	SQ	SSPK	MIX	OMR	CGQ
87-(4-1)-2_20210927-27COMP_A08.hid	87-(4-1)-2							

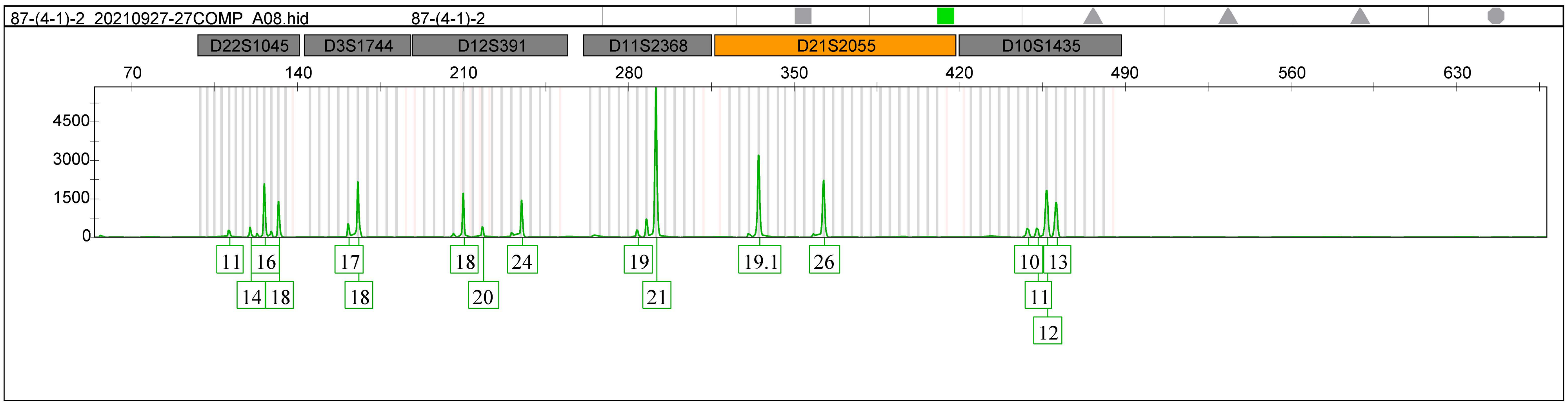

GeneMapper® ID-X 1.5

Sample File	Sample Name	SQO	SOS	SQ	SSPK	MIX	OMR	CGQ
87-(4-1)-2_20210927-27COMP_A08.hid	87-(4-1)-2							

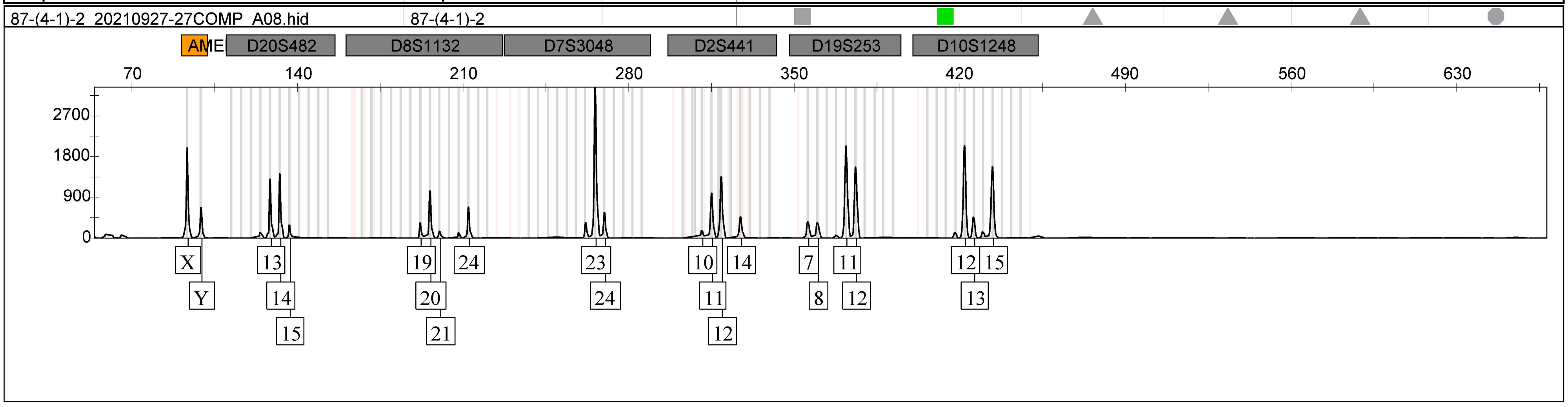

Sample File	Sample Name	SQO	SOS	SQ	SSPK	MIX	OMR	CGQ
87-(4-1)-2_20210927-27COMP_A08.hid	87-(4-1)-2							

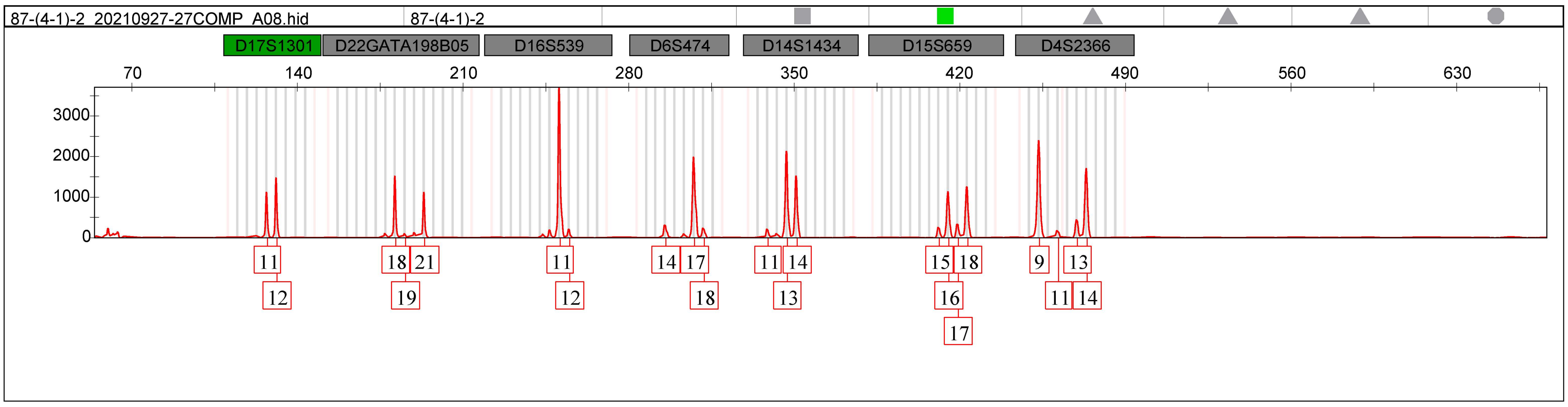

Sample File	Sample Name	SQO	SOS	SQ	SSPK	MIX	OMR	CGQ
87-(4-1)-3_20210927-27COMP_B08.hid	87-(4-1)-3							

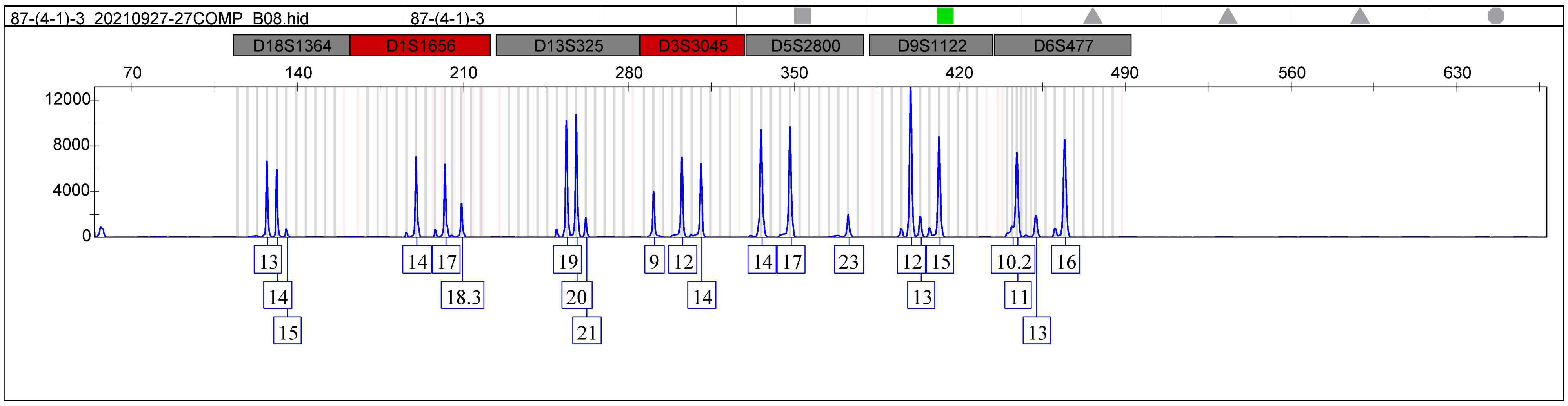

GeneMapper® ID-X 1.5

Sample File	Sample Name	SQO	SOS	SQ	SSPK	MIX	OMR	CGQ
87-(4-1)-3_20210927-27COMP_B08.hid	87-(4-1)-3							

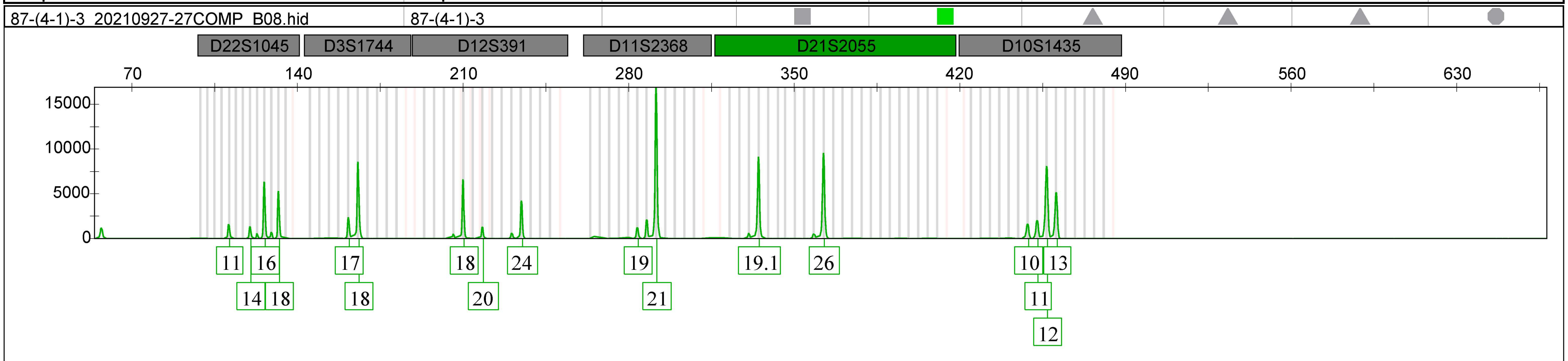

Sample File	Sample Name	SQO	SOS	SQ	SSPK	MIX	OMR	CGQ
87-(4-1)-3_20210927-27COMP_B08.hid	87-(4-1)-3							

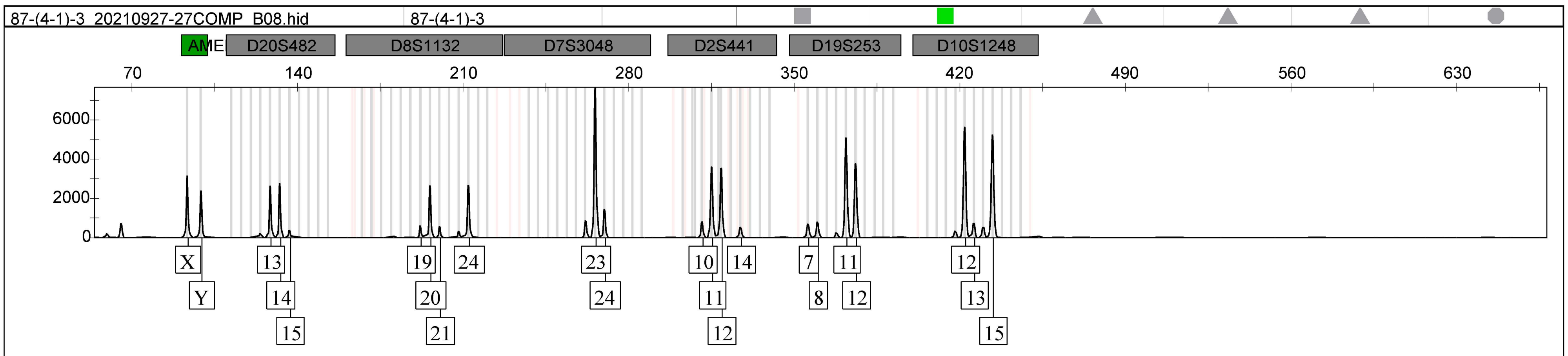

Sample File	Sample Name	SQO	SOS	SQ	SSPK	MIX	OMR	CGQ
87-(4-1)-3_20210927-27COMP_B08.hid	87-(4-1)-3							

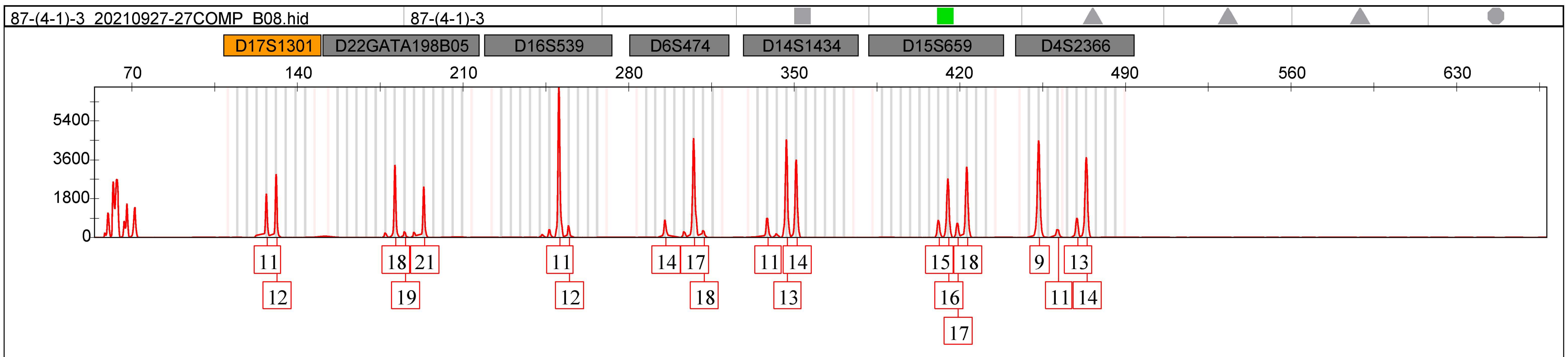

Sample File	Sample Name	SQO	SOS	SQ	SSPK	MIX	OMR	CGQ
87-(8-1)-1_20210927-27COMP_E07.hid	87-(8-1)-1							

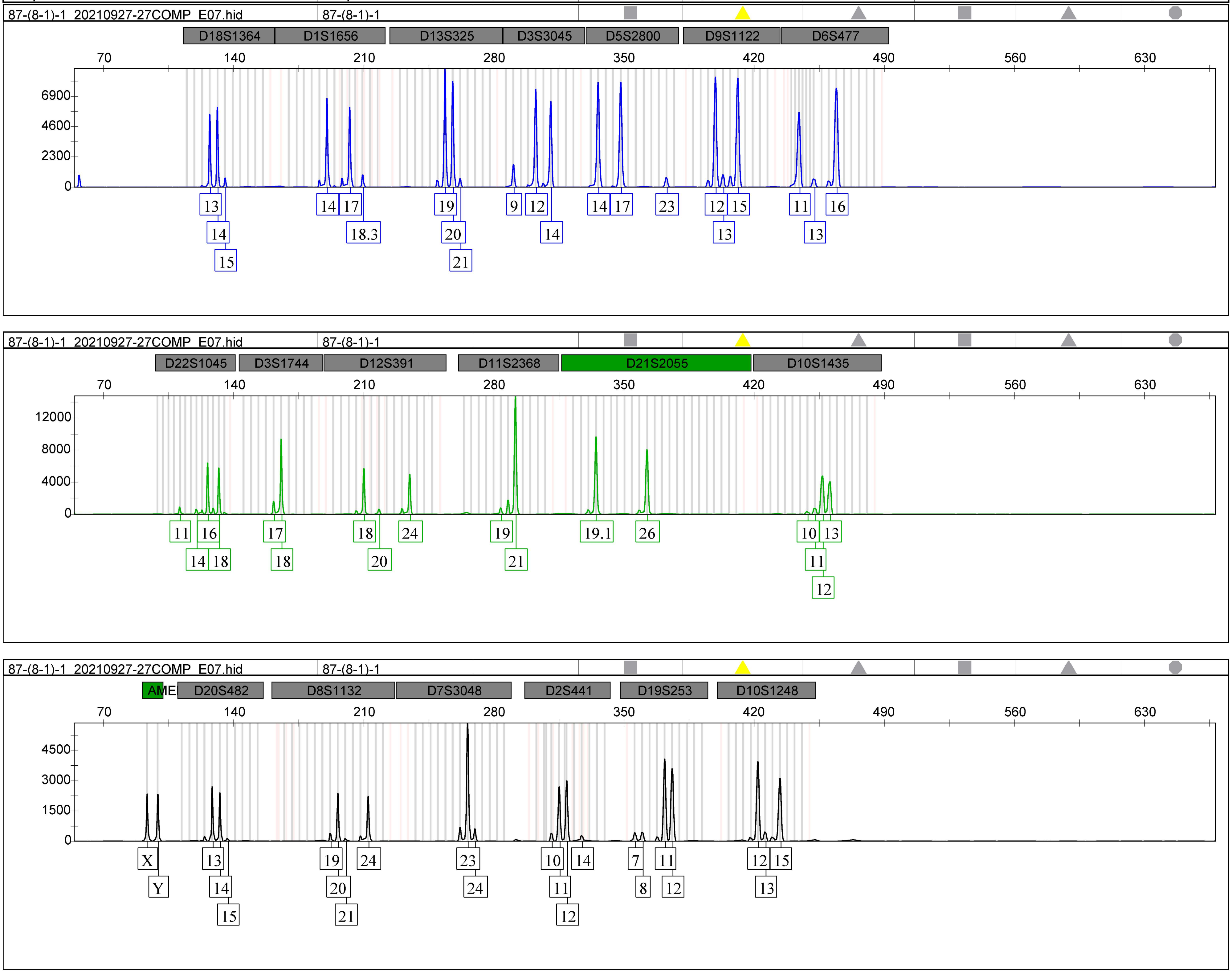

Project: 20210927-27COMP

GeneMapper® ID-X 1.5

Sample File	Sample Name	SQO	SOS	SQ	SSPK	MIX	OMR	CGQ
87-(8-1)-1_20210927-27COMP_E07.hid	87-(8-1)-1							

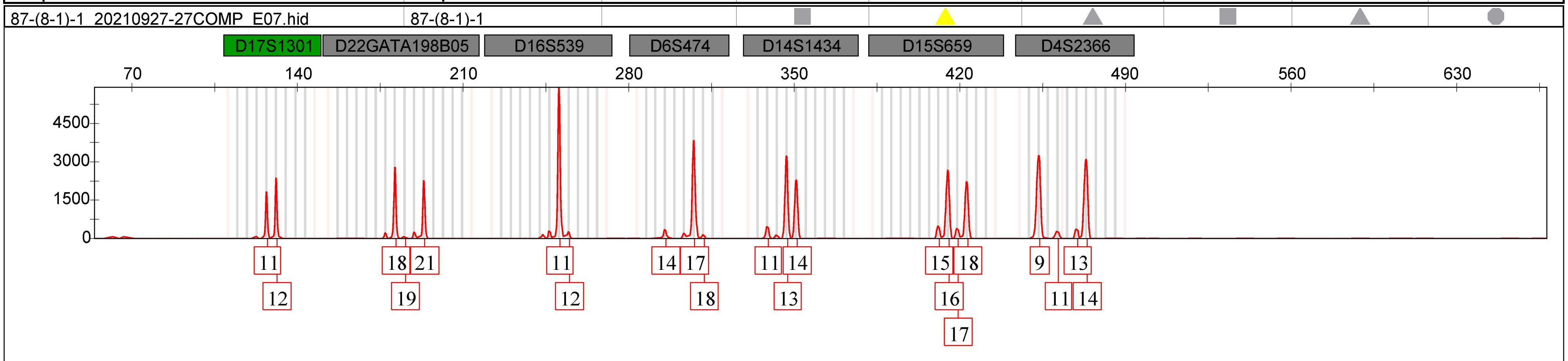

Sample File	Sample Name	SQO	SOS	SQ	SSPK	MIX	OMR	CGQ
87-(8-1)-2_20210927-27COMP_F07.hid	87-(8-1)-2							

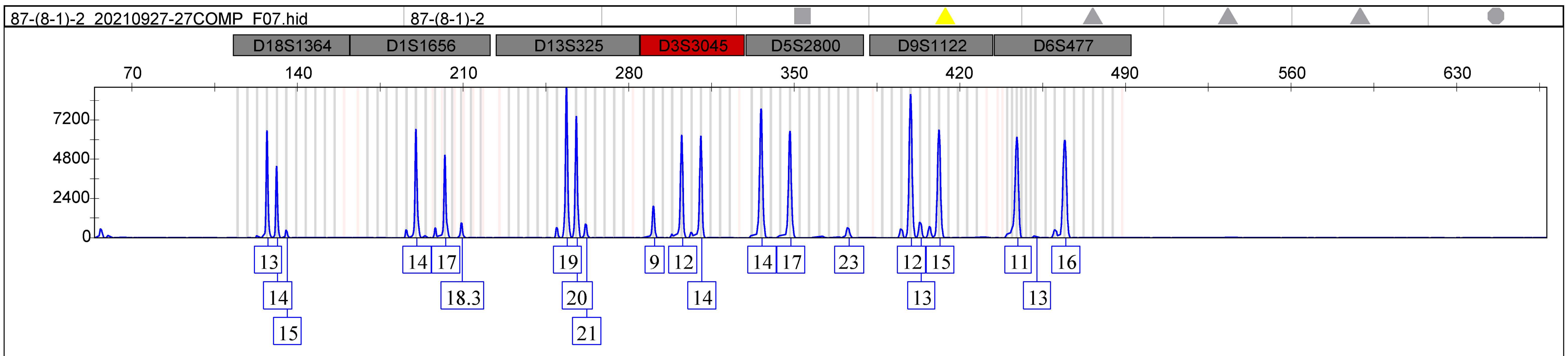

Sample File	Sample Name	SQO	SOS	SQ	SSPK	MIX	OMR	CGQ
87-(8-1)-2_20210927-27COMP_F07.hid	87-(8-1)-2							

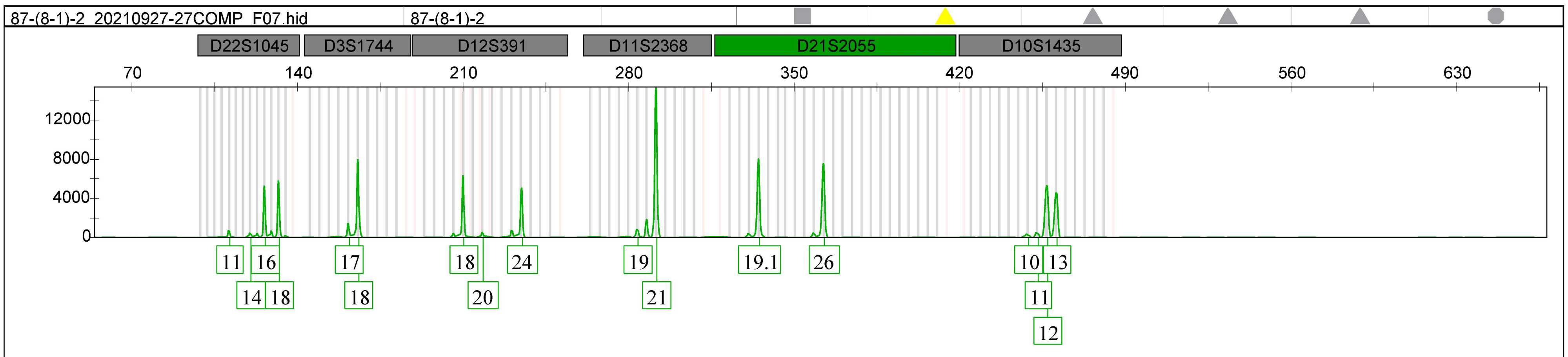

Project: 20210927-27COMP

GeneMapper® ID-X 1.5

Sample File	Sample Name	SQO	SOS	SQ	SSPK	MIX	OMR	CGQ
87-(8-1)-2 20210927-27COMP F07.hid	87-(8-1)-2							

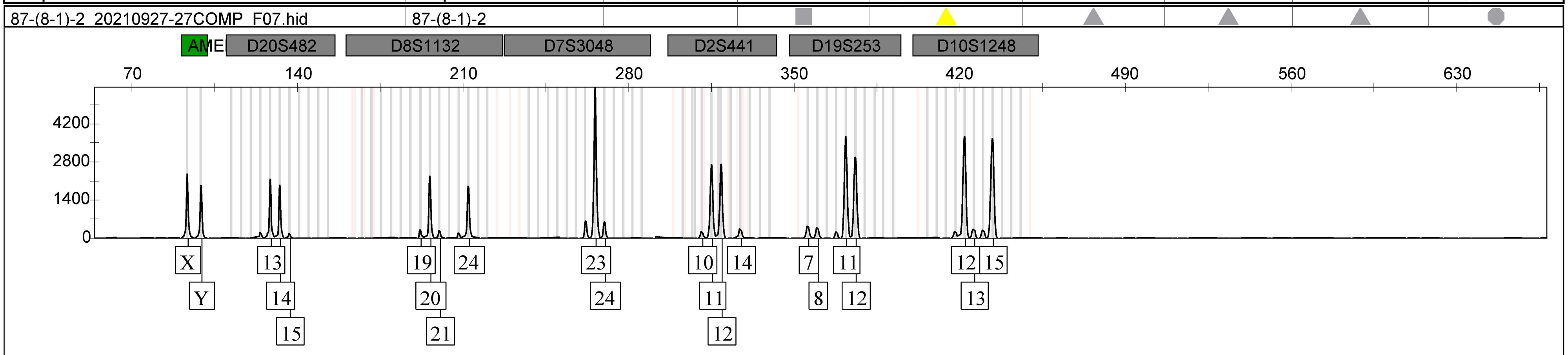

Sample File	Sample Name	SQO	SOS	SQ	SSPK	MIX	OMR	CGQ
87-(8-1)-2 20210927-27COMP F07.hid	87-(8-1)-2							

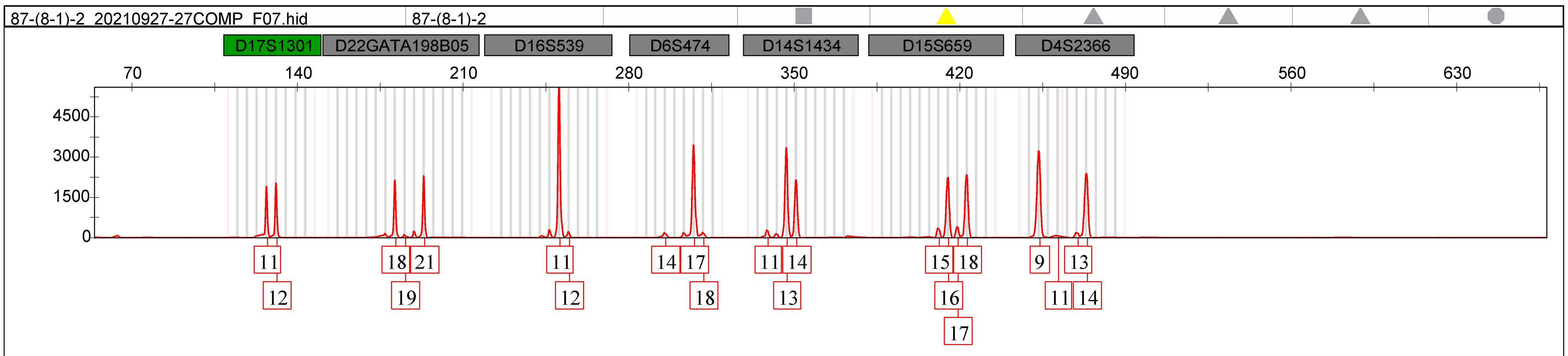

Sample File	Sample Name	SQO	SOS	SQ	SSPK	MIX	OMR	CGQ
87-(8-1)-3 20210927-27COMP G07.hid	87-(8-1)-3							

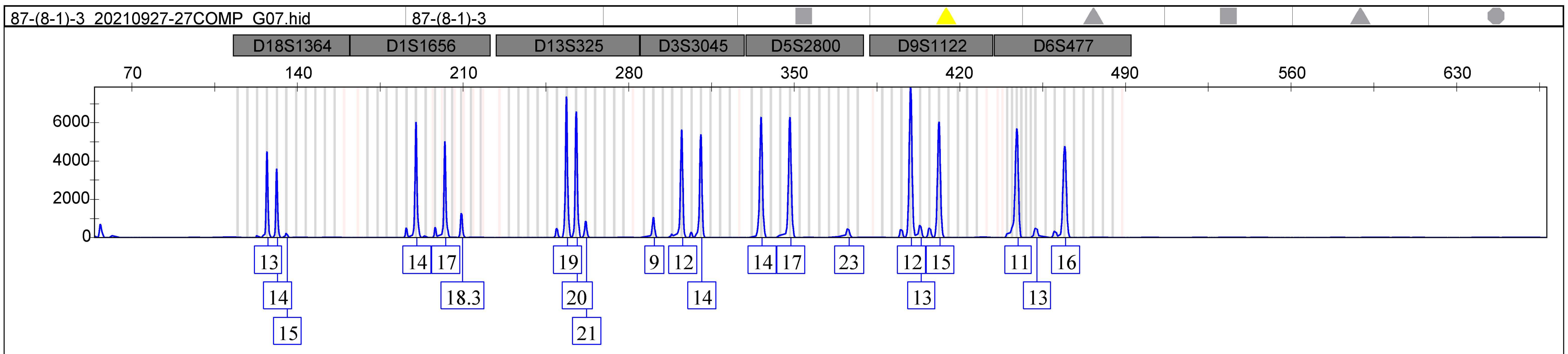

Project: 20210927-27COMP

GeneMapper® ID-X 1.5

Sample File	Sample Name	SQO	SOS	SQ	SSPK	MIX	OMR	CGQ
87-(8-1)-3_20210927-27COMP_G07.hid	87-(8-1)-3							

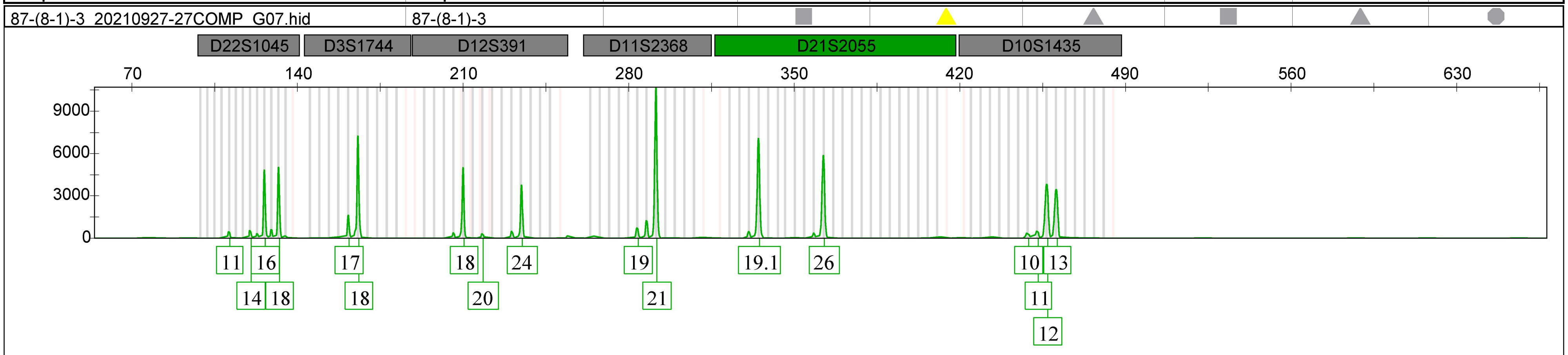

87-(8-1)-3_20210927-27COMP_G07.hid | 87-(8-1)-3

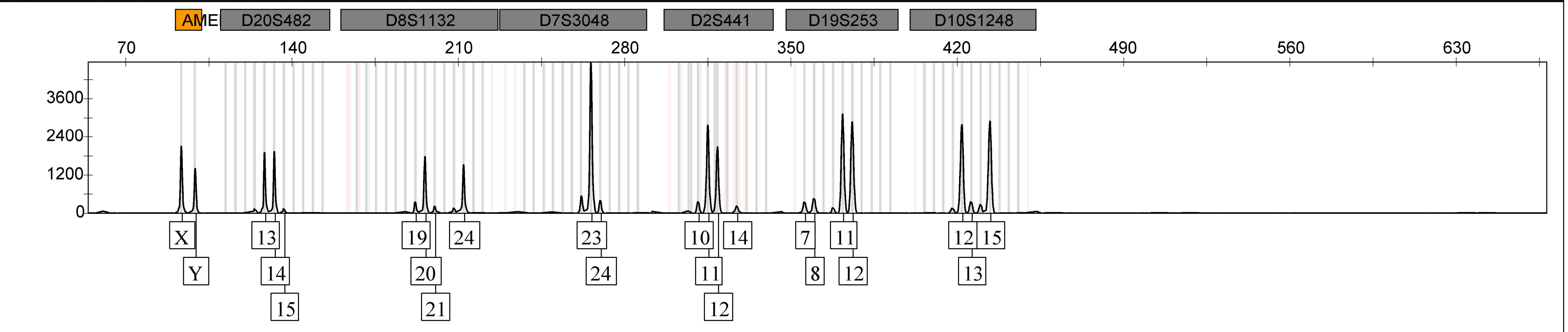

87-(8-1)-3_20210927-27COMP_G07.hid | 87-(8-1)-3

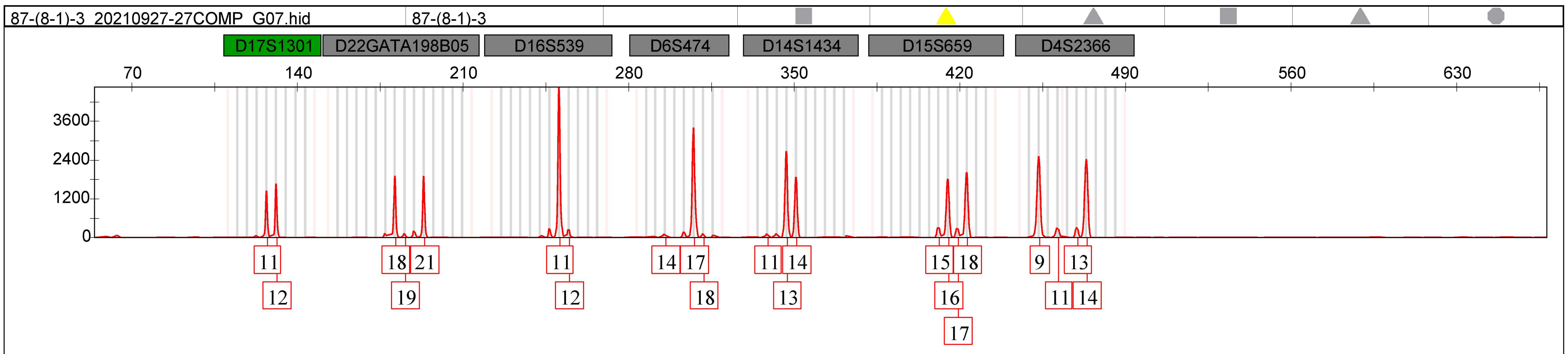

Project: 20210927-27COMP

GeneMapper® ID-X 1.5

Sample File	Sample Name	SQO	SOS	SQ	SSPK	MIX	OMR	CGQ
87-(19-1)-1_20210927-27COMP_B07.hid	87-(19-1)-1							

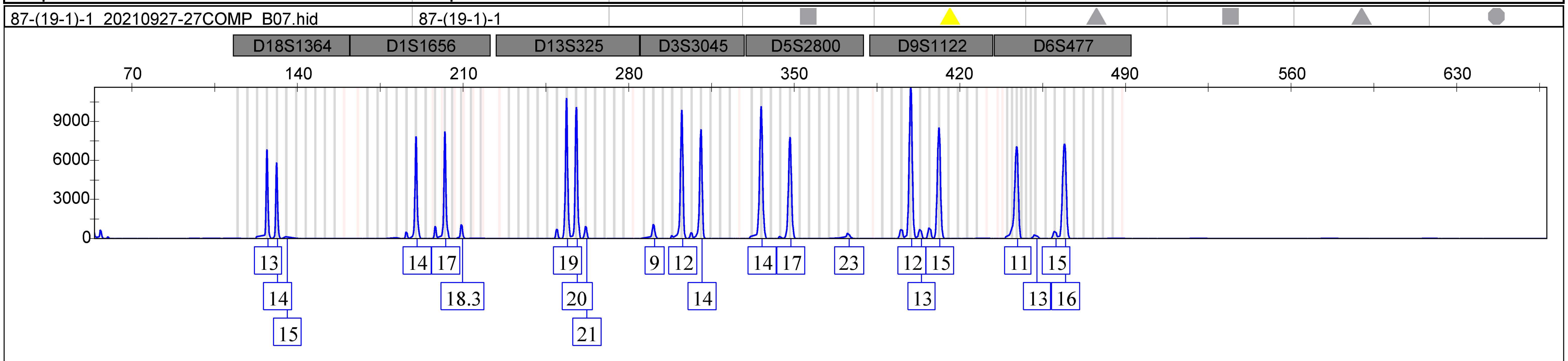

87-(19-1)-1_20210927-27COMP_B07.hid 87-(19-1)-1

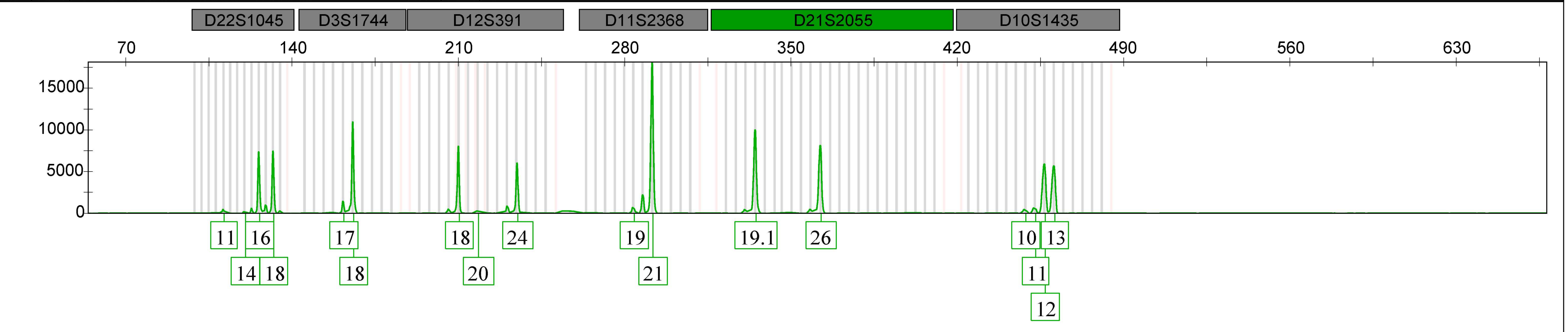

87-(19-1)-1_20210927-27COMP_B07.hid 87-(19-1)-1

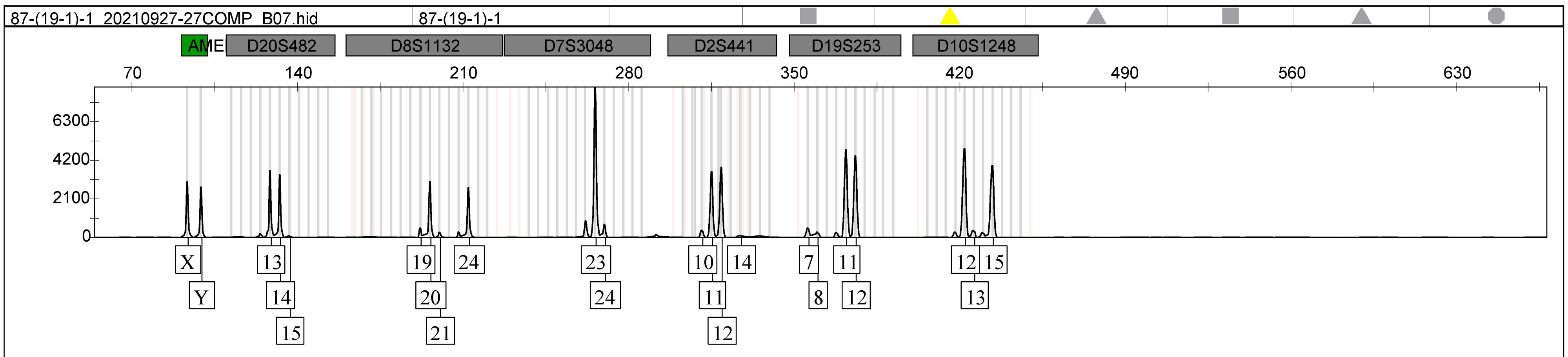

Project: 20210927-27COMP

GeneMapper® ID-X 1.5

Sample File	Sample Name	SQO	SOS	SQ	SSPK	MIX	OMR	CGQ
87-(19-1)-1_20210927-27COMP_B07.hid	87-(19-1)-1							

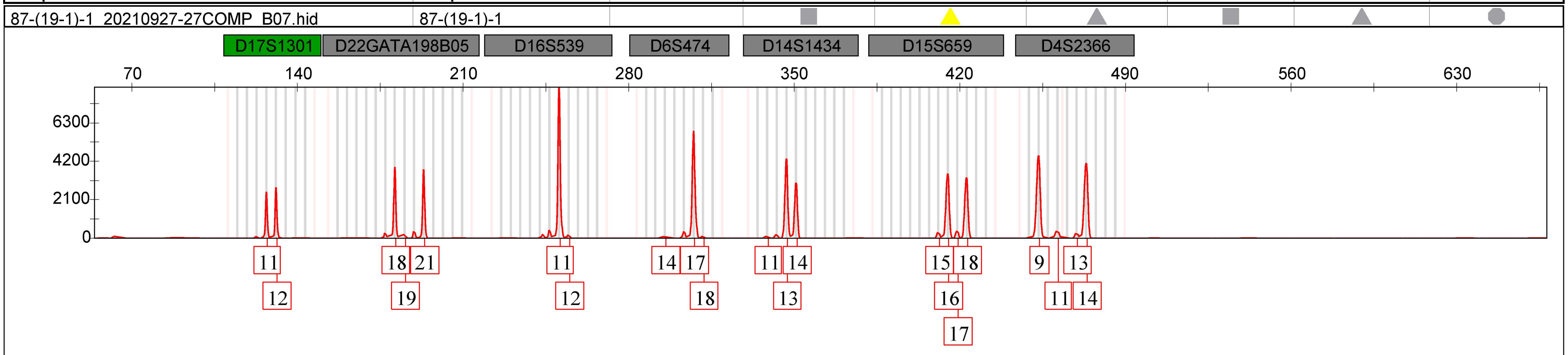

87-(19-1)-2_20210927-27COMP_C07.hid	87-(19-1)-2							

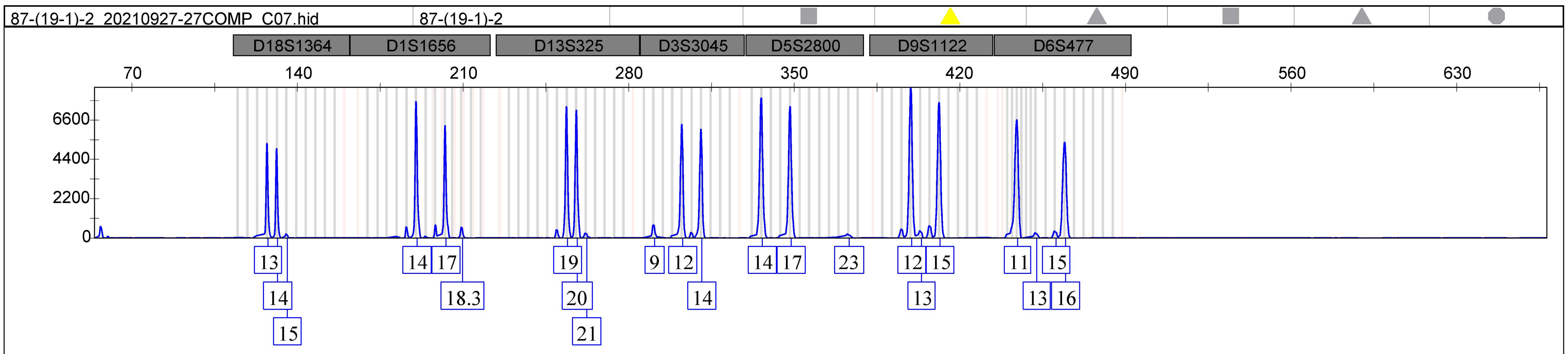

87-(19-1)-2_20210927-27COMP_C07.hid	87-(19-1)-2							

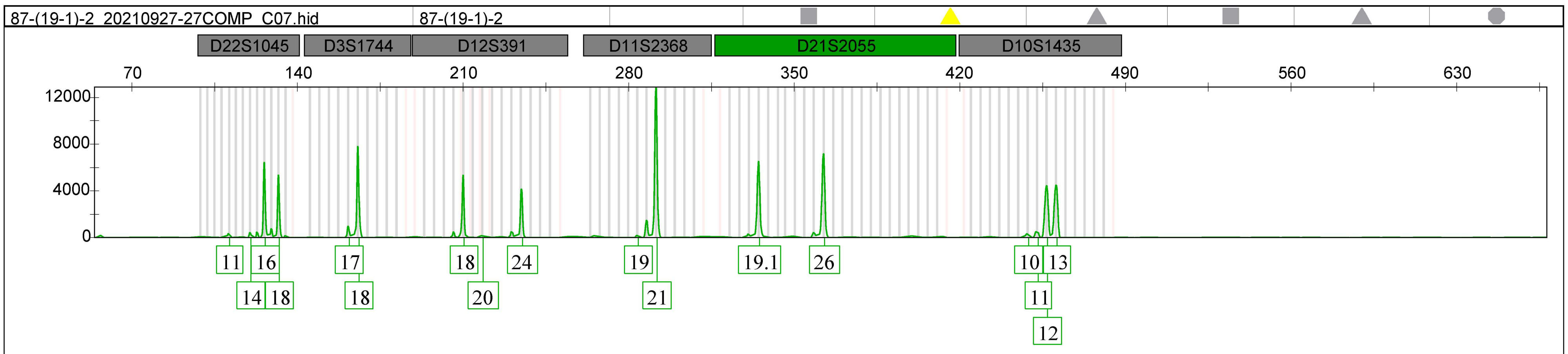

Project: 20210927-27COMP

GeneMapper® ID-X 1.5

Sample File	Sample Name	SQO	SOS	SQ	SSPK	MIX	OMR	CGQ
87-(19-1)-2_20210927-27COMP_C07.hid	87-(19-1)-2							

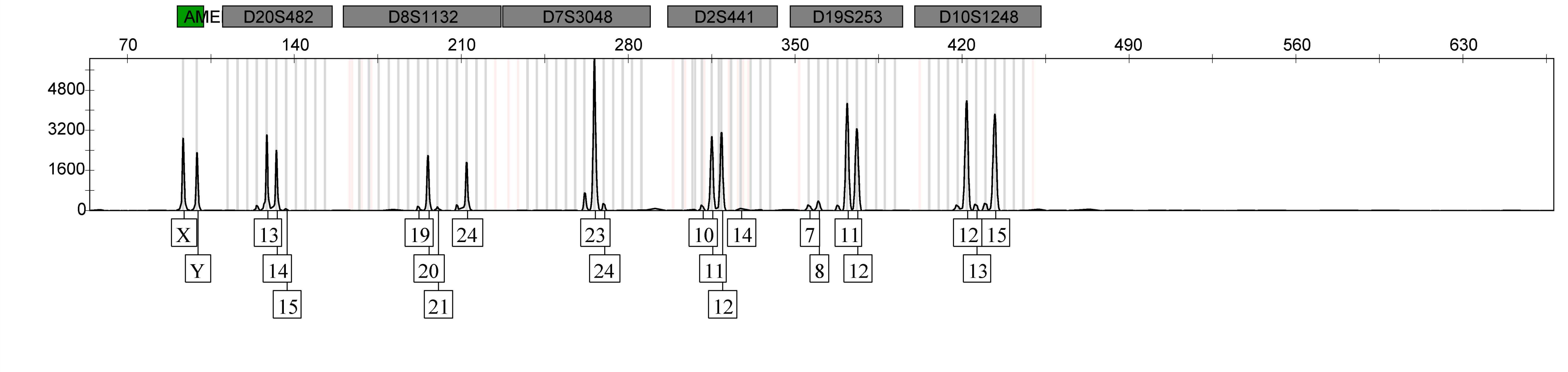

Sample File	Sample Name	SQO	SOS	SQ	SSPK	MIX	OMR	CGQ
87-(19-1)-2_20210927-27COMP_C07.hid	87-(19-1)-2							

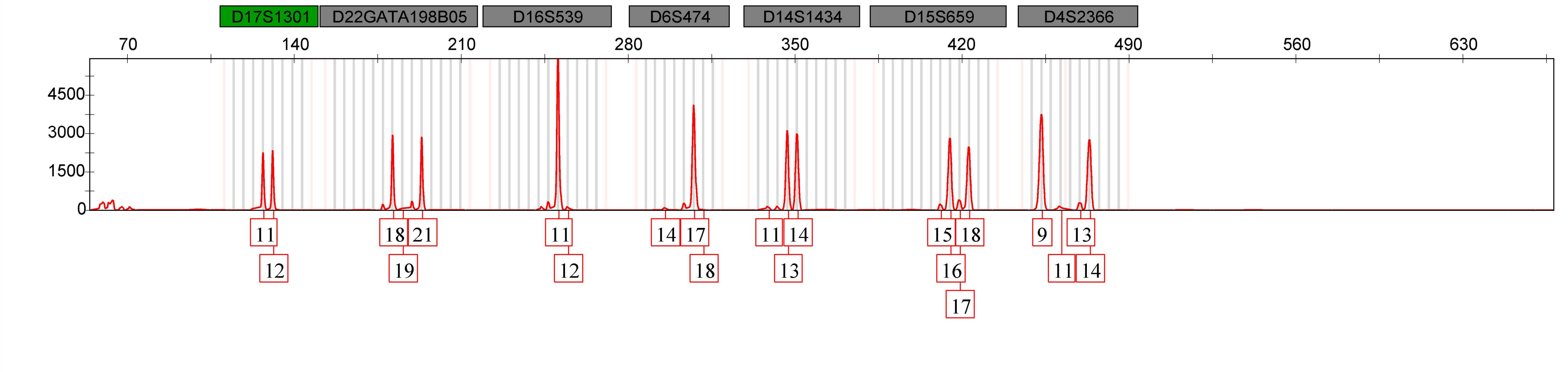

Sample File	Sample Name	SQO	SOS	SQ	SSPK	MIX	OMR	CGQ
87-(19-1)-3_20210927-27COMP_D07.hid	87-(19-1)-3							

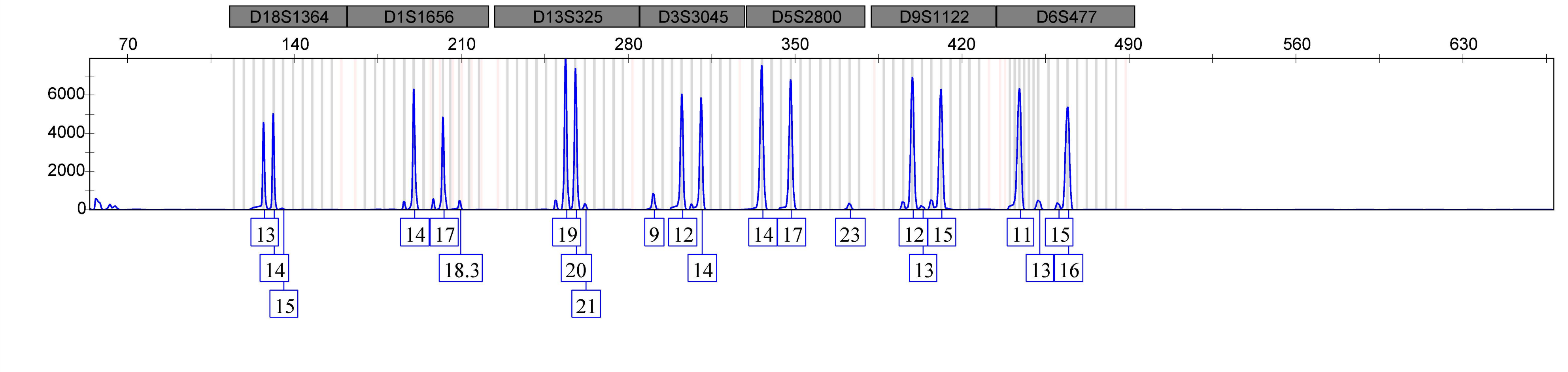

GeneMapper® ID-X 1.5

Sample File	Sample Name	SQO	SOS	SQ	SSPK	MIX	OMR	CGQ
87-(19-1)-3_20210927-27COMP_D07.hid	87-(19-1)-3							

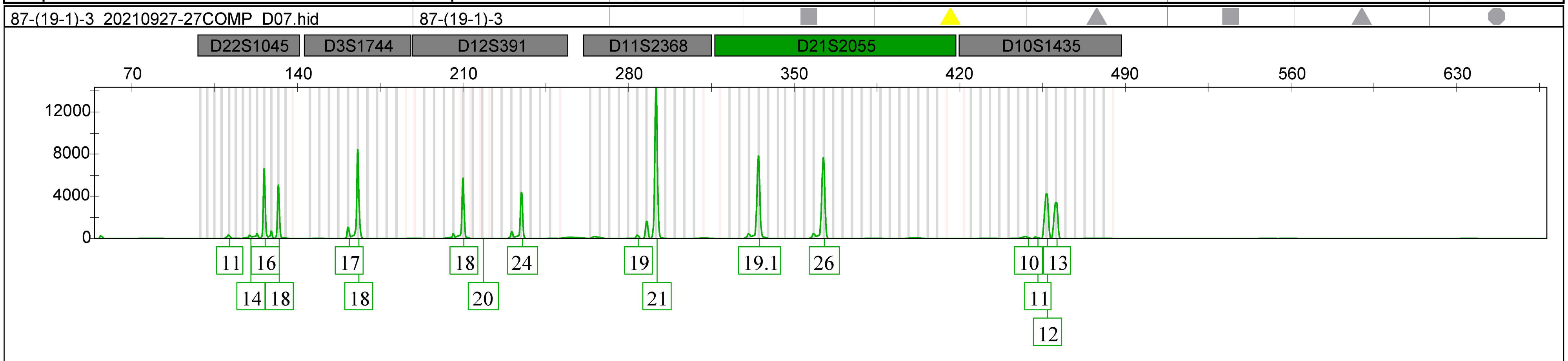

87-(19-1)-3_20210927-27COMP_D07.hid 87-(19-1)-3

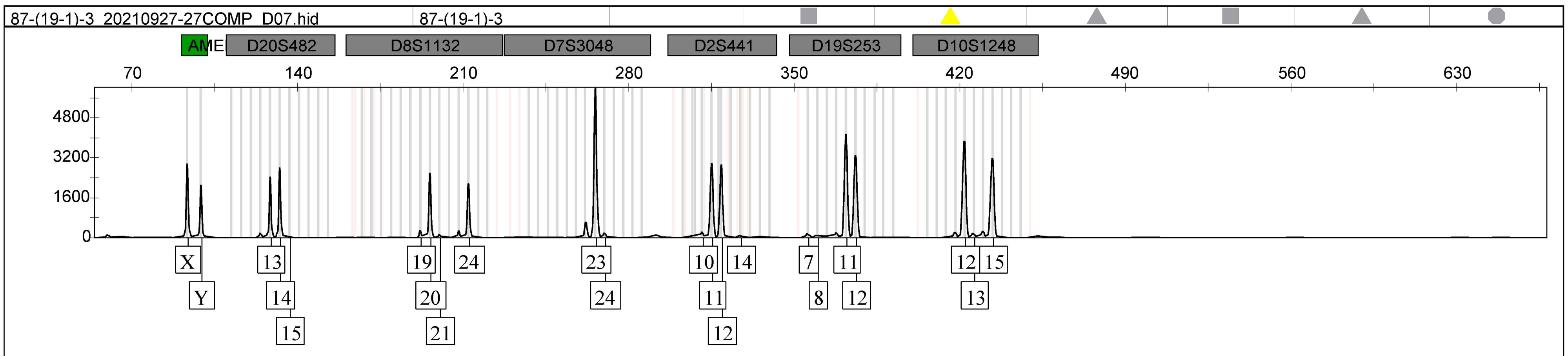

87-(19-1)-3_20210927-27COMP_D07.hid 87-(19-1)-3

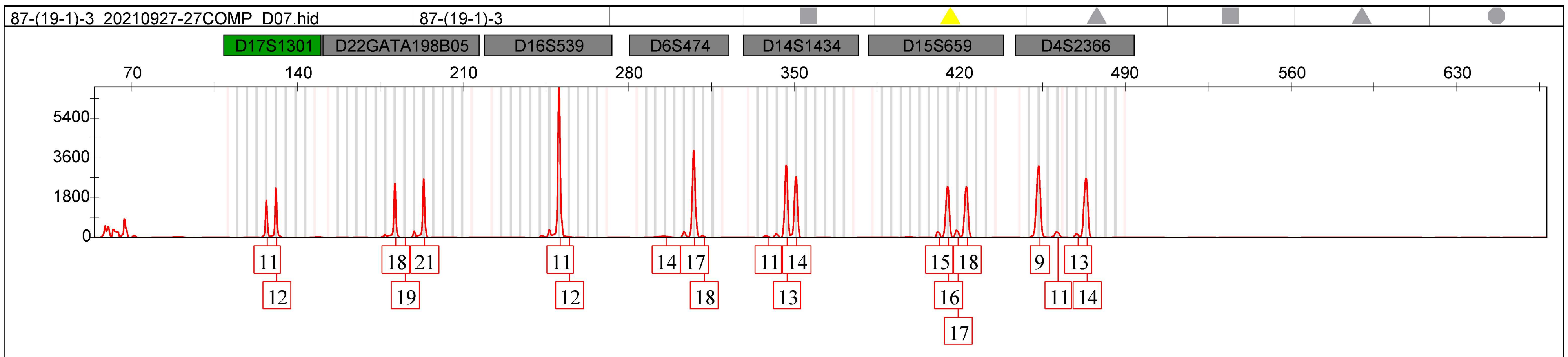

Supplement: Supplementary file 1 [file DataSheet1.ZIP › SM/Supplementary Figure 14.pdf]
