# Supplementary figures and images for: Developmental Validation of the Novel Five-Dye-Labeled Multiplex Autosomal STR Panel and Its Forensic Efficiency Evaluation
Source: Front Genet. 2022 May 31;13:897650. doi: 10.3389/fgene.2022.897650 (PMC9194853; doi:10.3389/fgene.2022.897650)

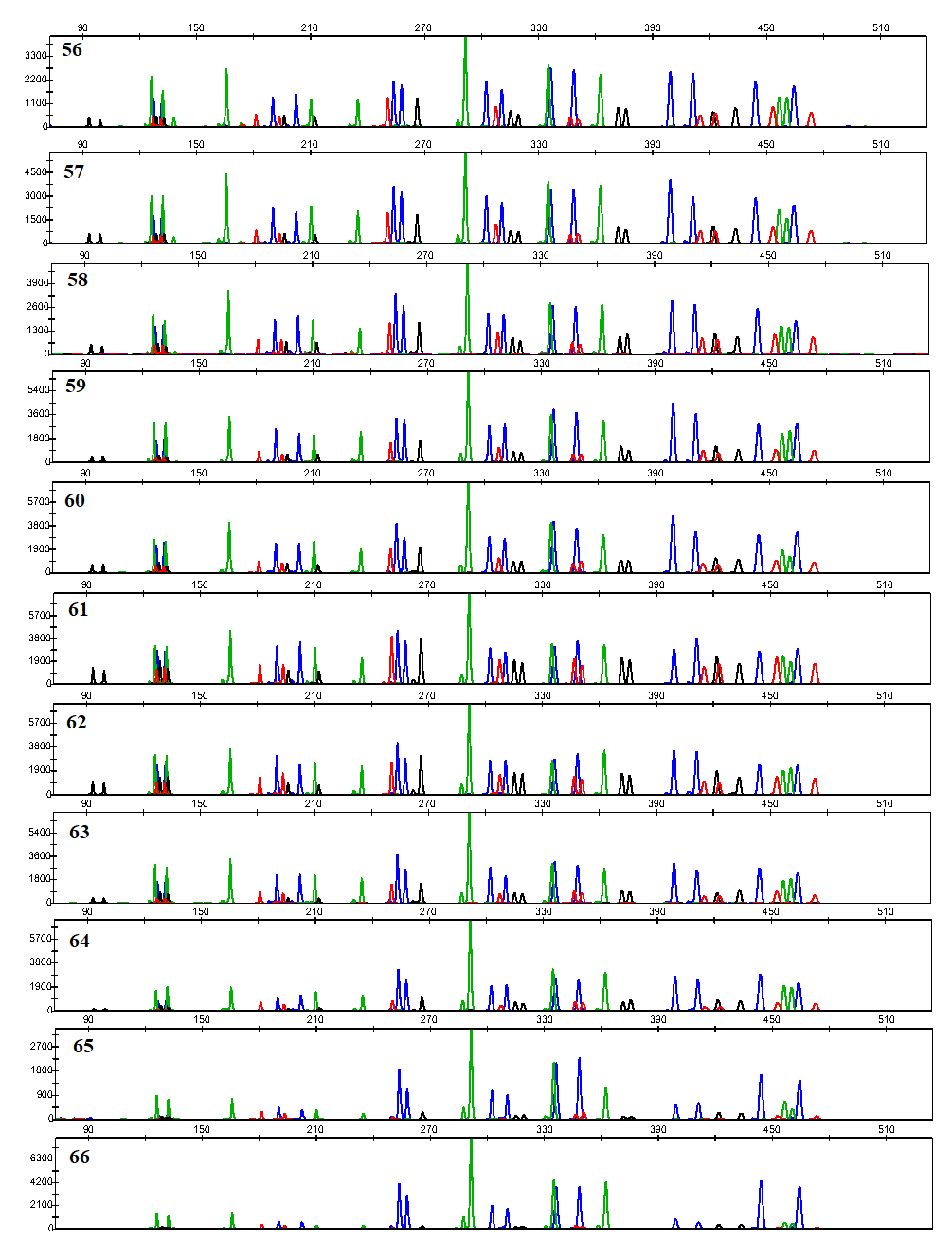

Supplement: Supplementary file 1 [file DataSheet1.ZIP › SM/Supplementary Figure 1.tif]

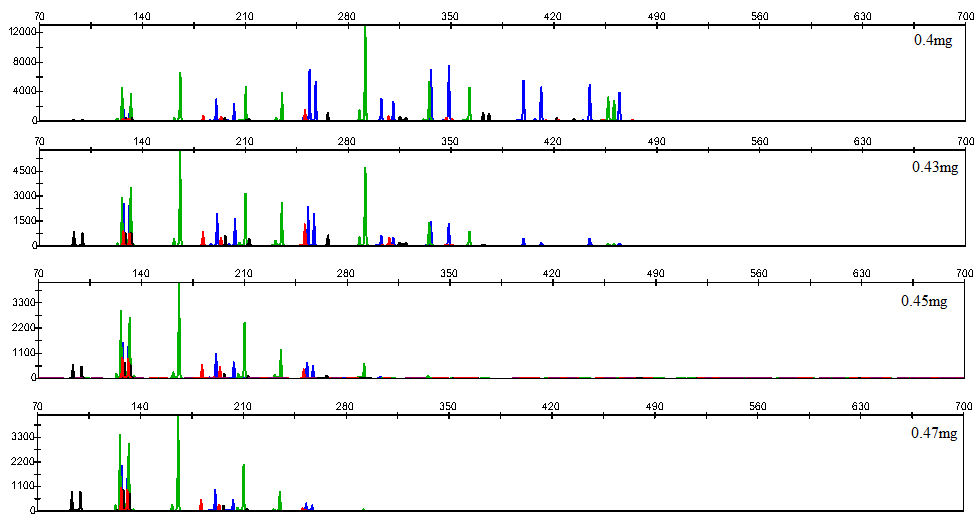

Supplement: Supplementary file 1 [file DataSheet1.ZIP › SM/Supplementary Figure 10.tif]

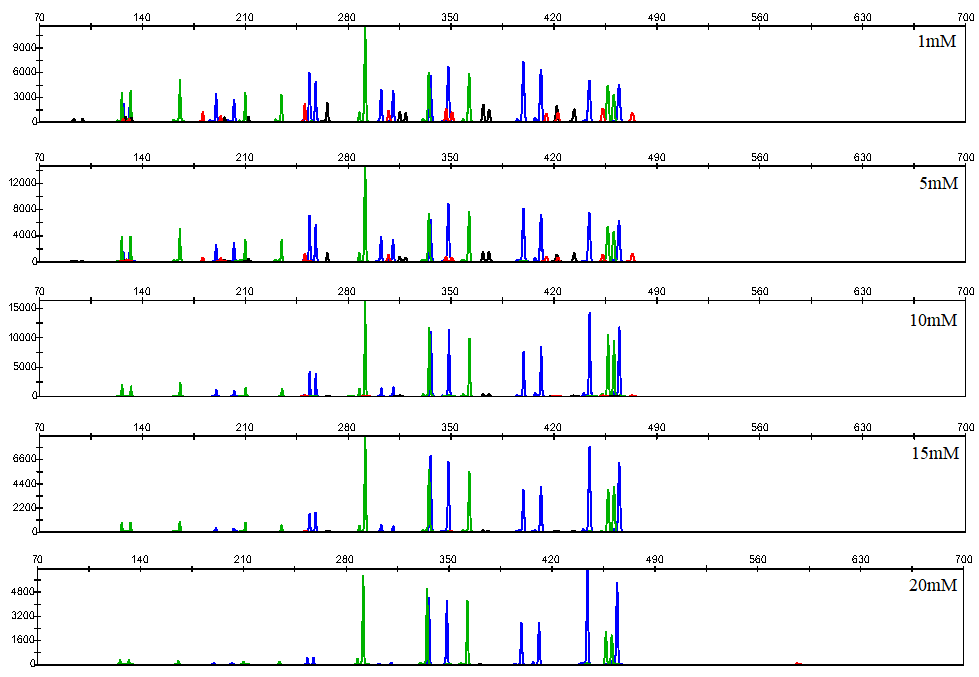

Supplement: Supplementary file 1 [file DataSheet1.ZIP › SM/Supplementary Figure 11.tif]

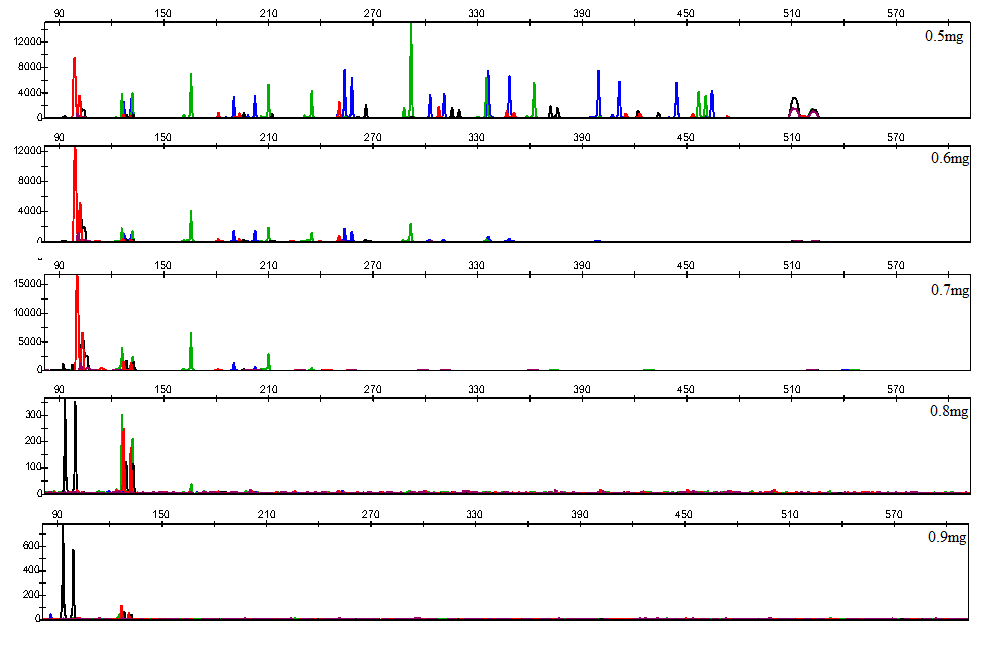

Supplement: Supplementary file 1 [file DataSheet1.ZIP › SM/Supplementary Figure 12.tif]

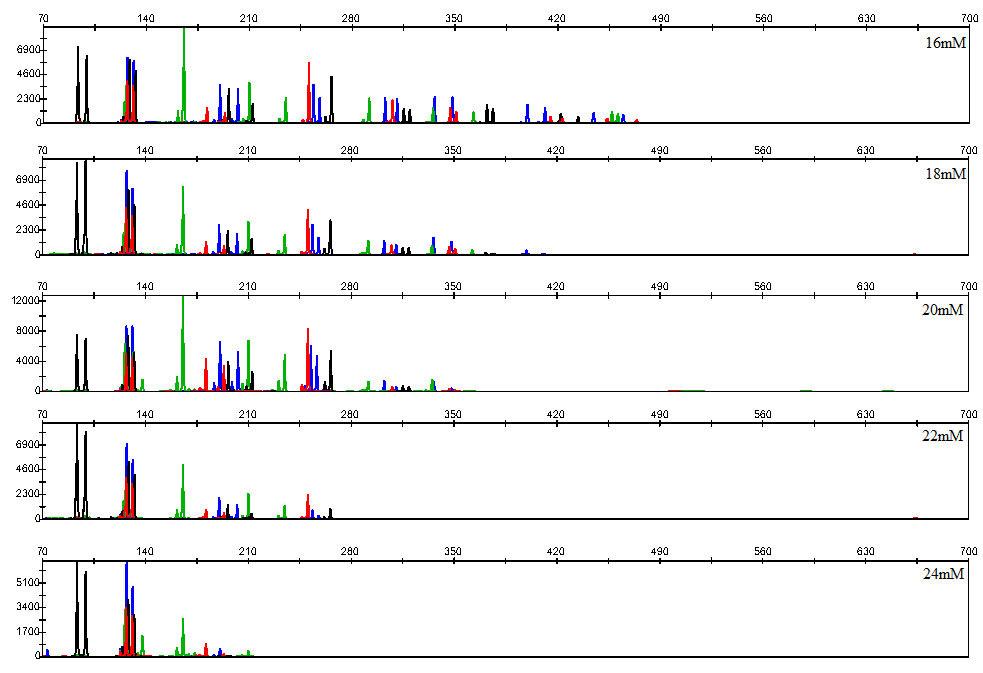

Supplement: Supplementary file 1 [file DataSheet1.ZIP › SM/Supplementary Figure 13.tif]

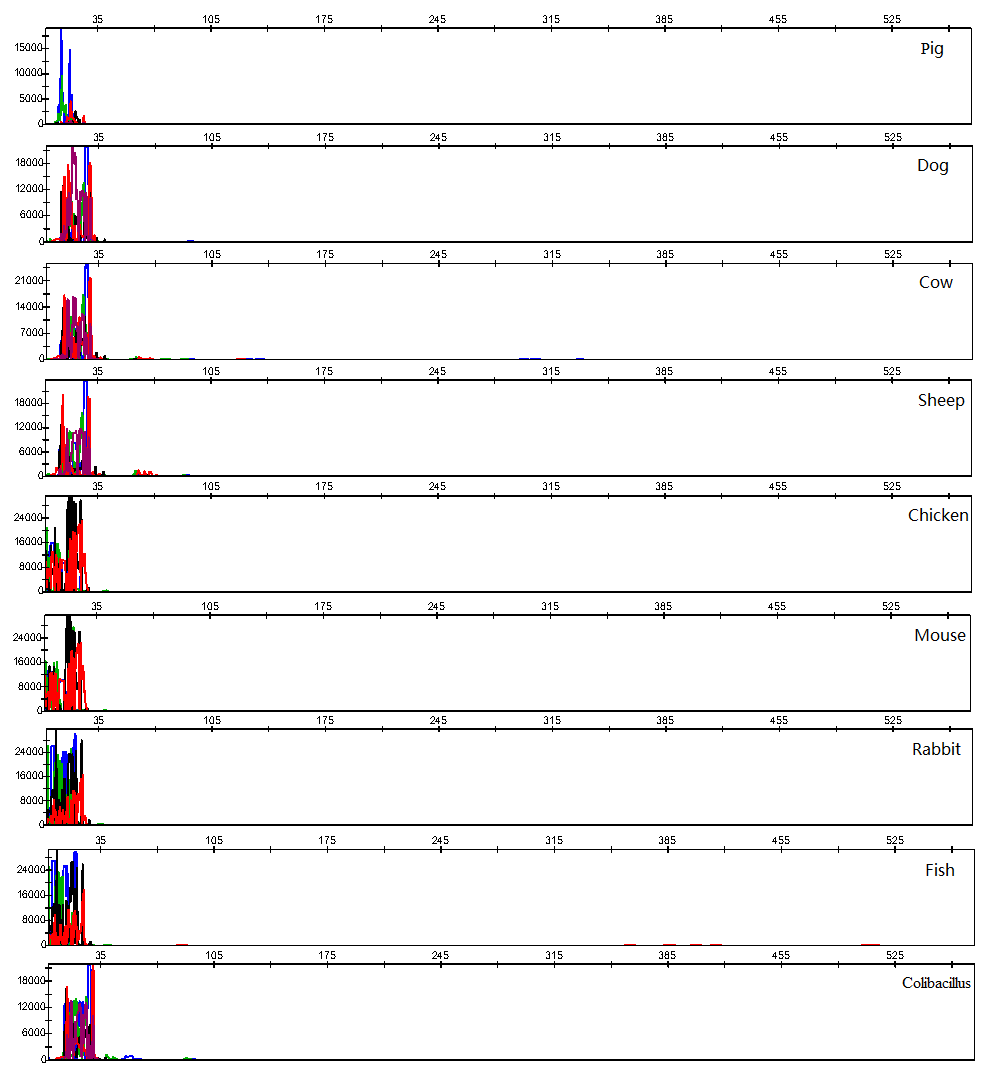

Supplement: Supplementary file 1 [file DataSheet1.ZIP › SM/Supplementary Figure 15.tif]

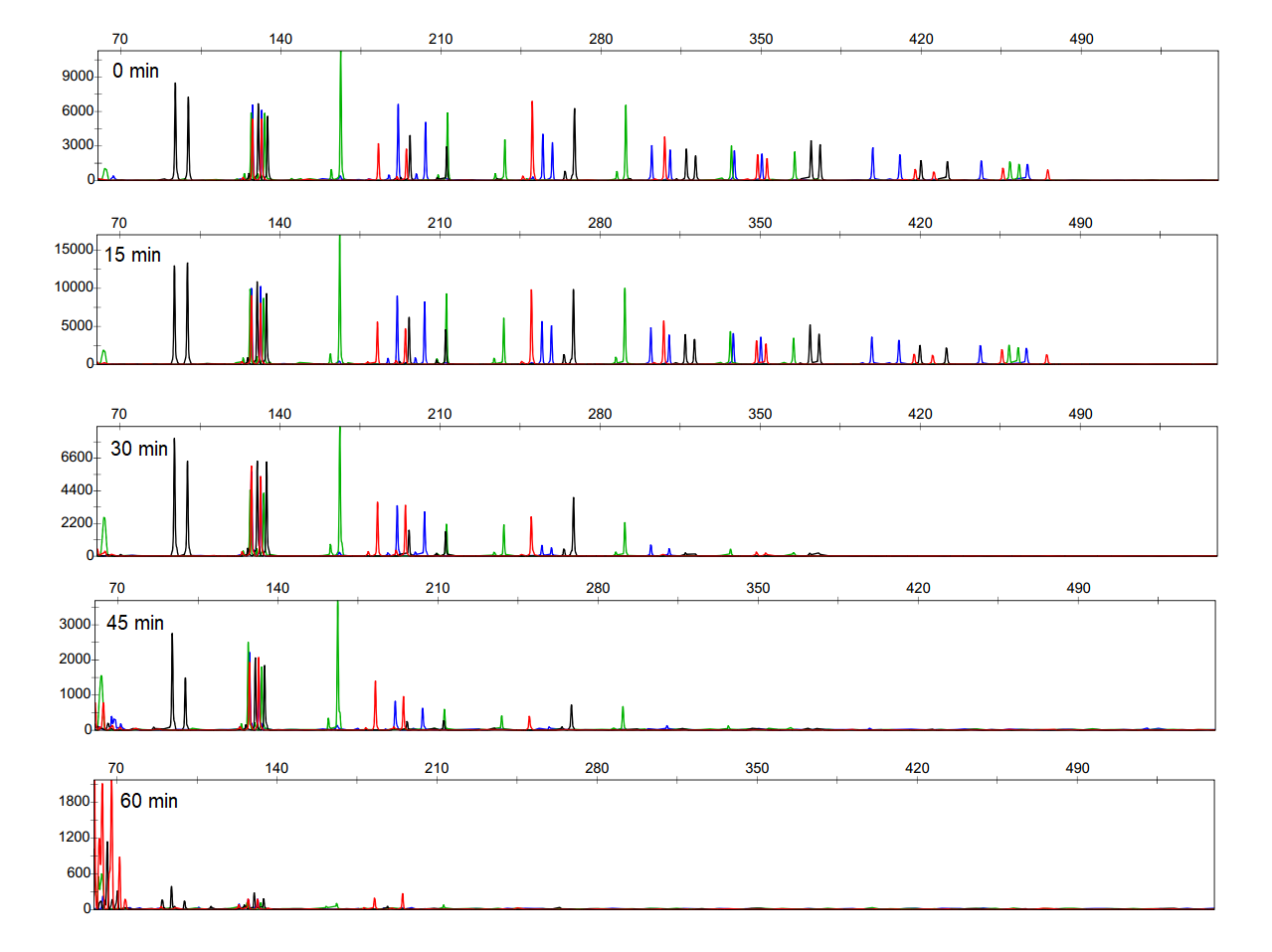

Supplement: Supplementary file 1 [file DataSheet1.ZIP › SM/Supplementary Figure 16.tif]

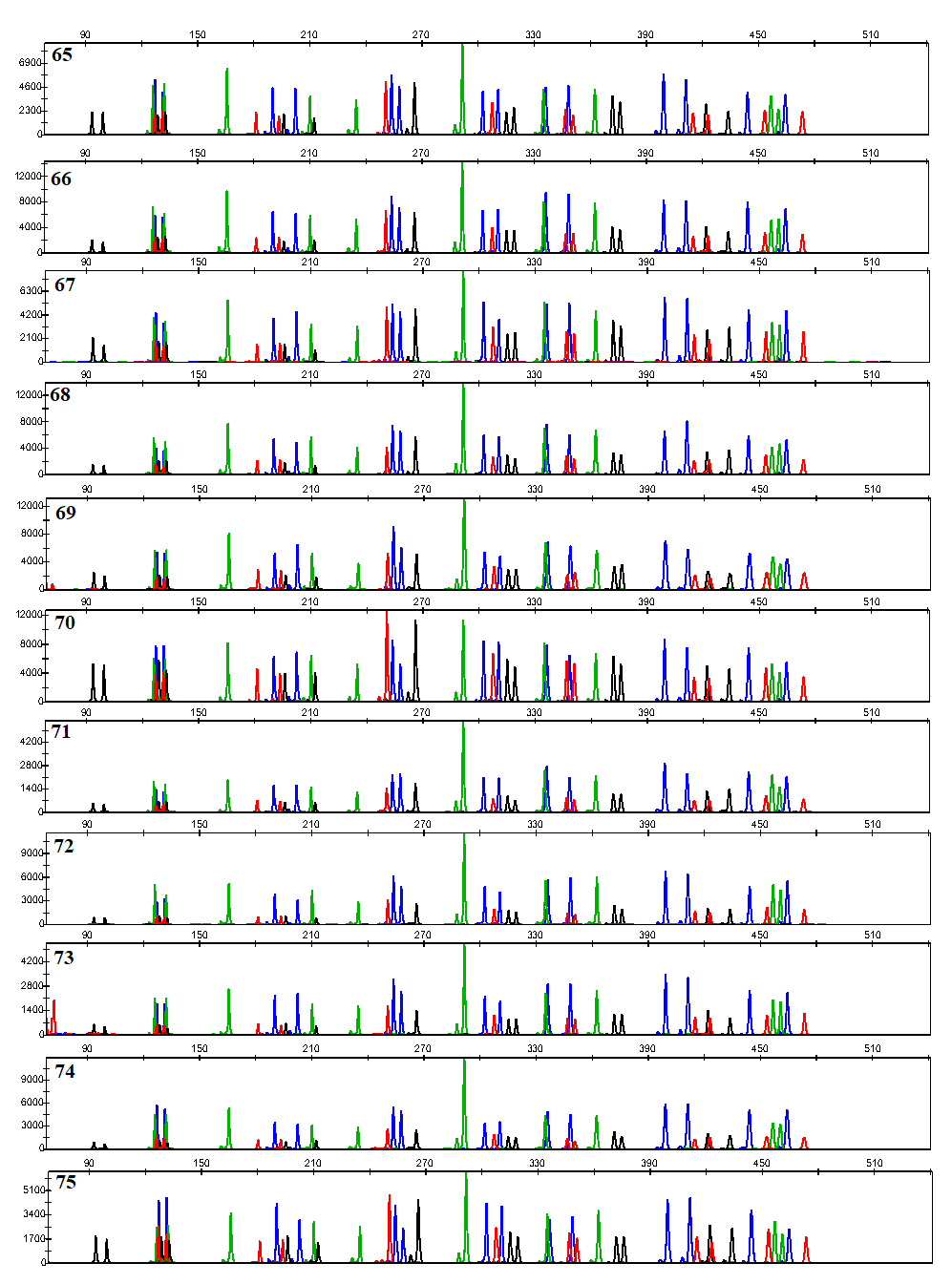

Supplement: Supplementary file 1 [file DataSheet1.ZIP › SM/Supplementary Figure 2.tif]

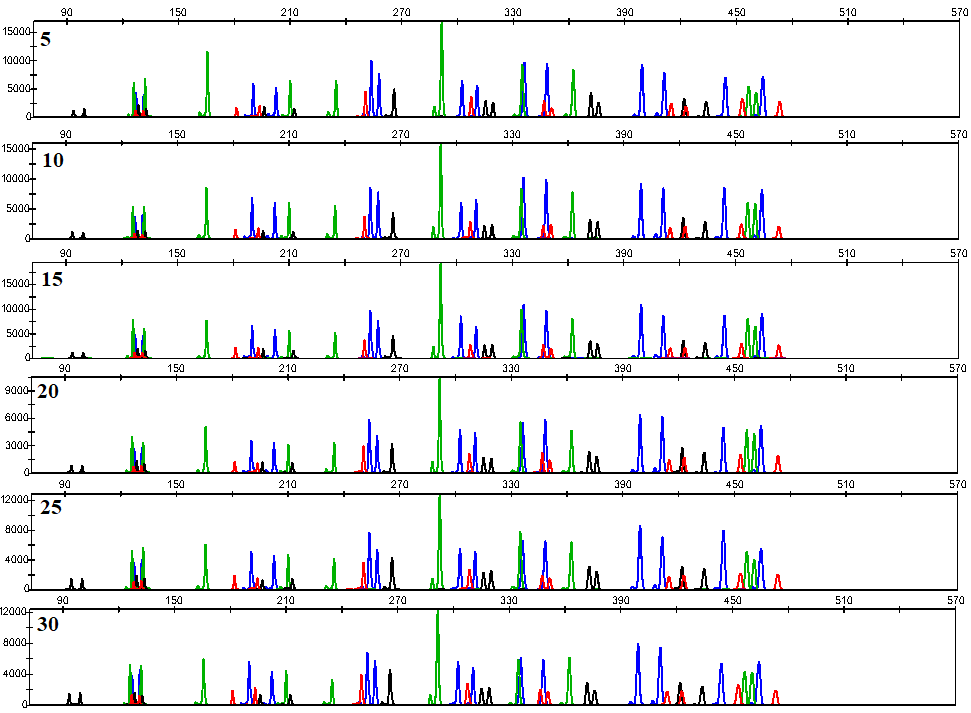

Supplement: Supplementary file 1 [file DataSheet1.ZIP › SM/Supplementary Figure 3.tif]

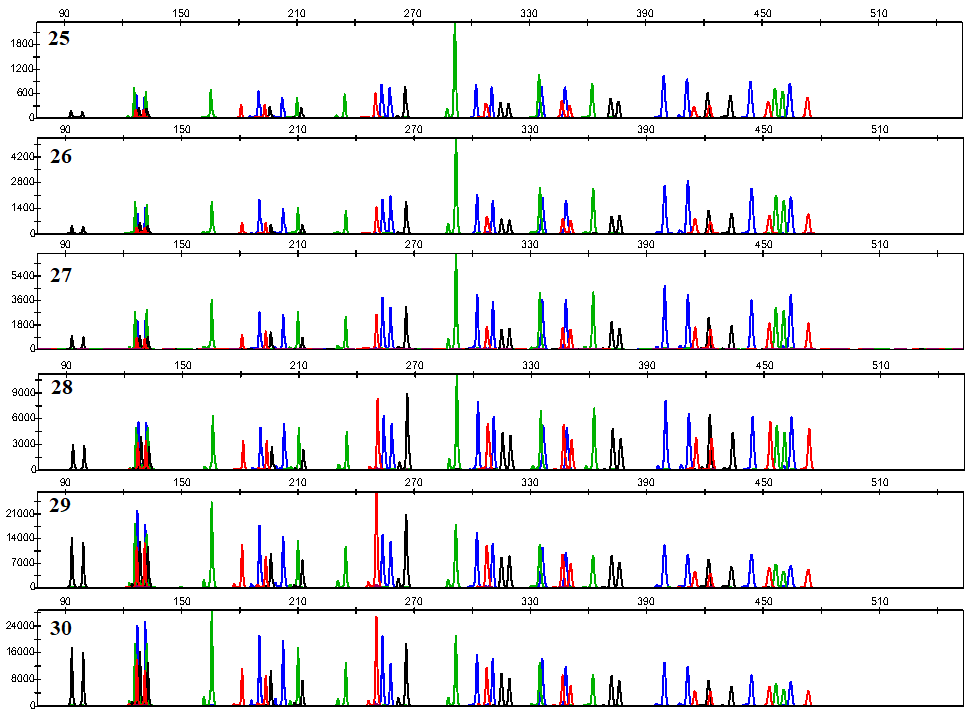

Supplement: Supplementary file 1 [file DataSheet1.ZIP › SM/Supplementary Figure 4.tif]

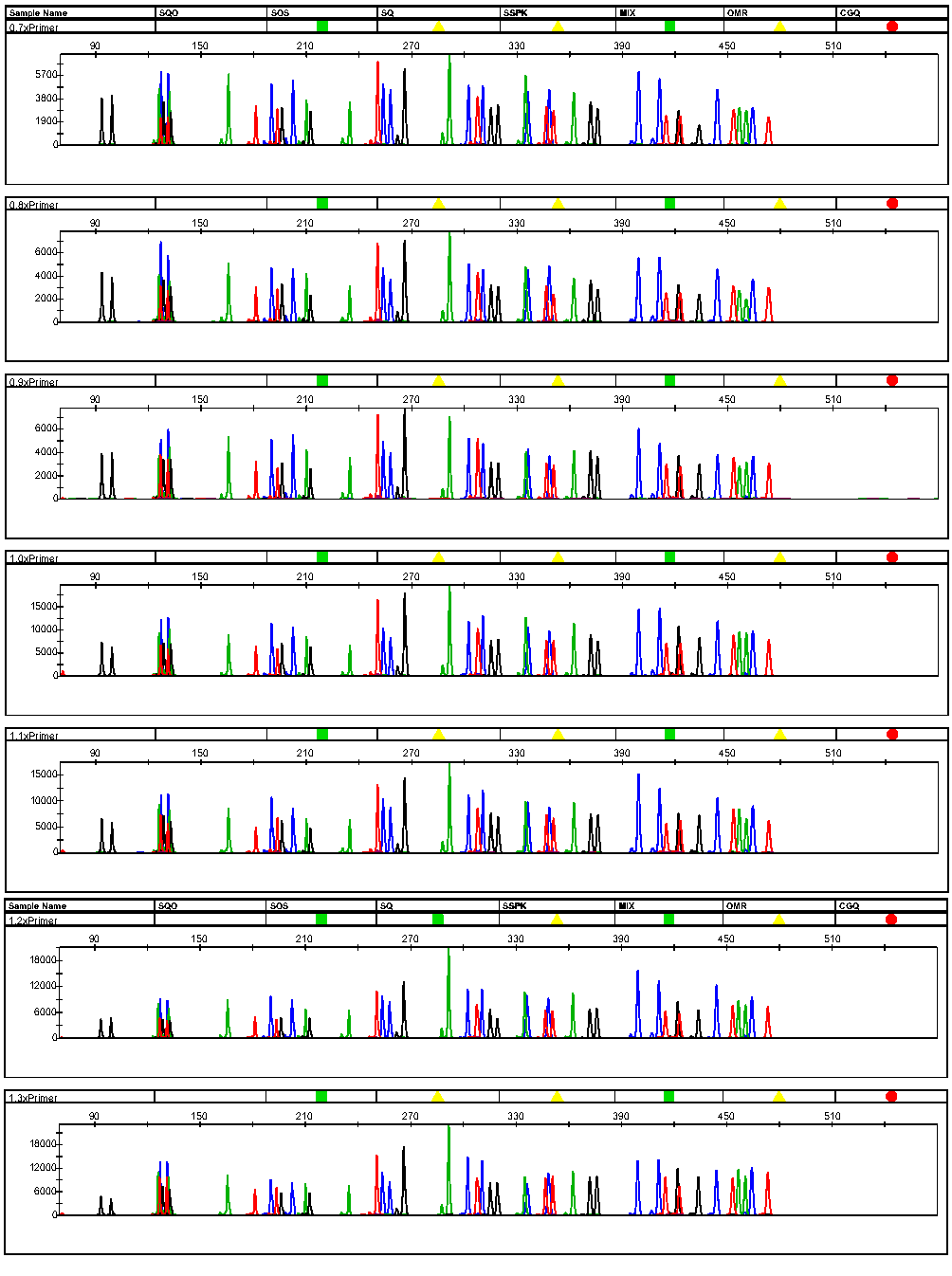

Supplement: Supplementary file 1 [file DataSheet1.ZIP › SM/Supplementary Figure 5.tiff]

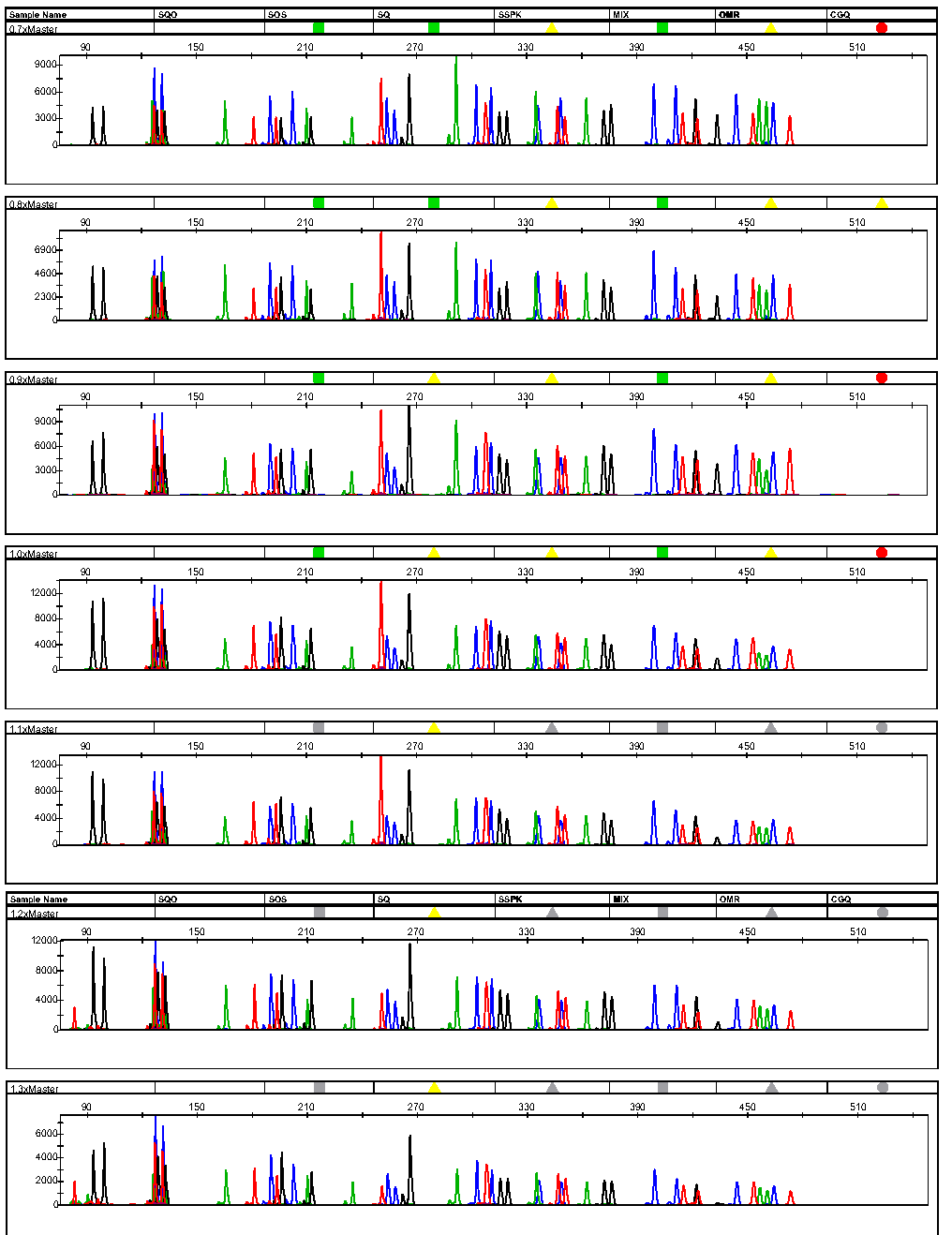

Supplement: Supplementary file 1 [file DataSheet1.ZIP › SM/Supplementary Figure 6.tiff]

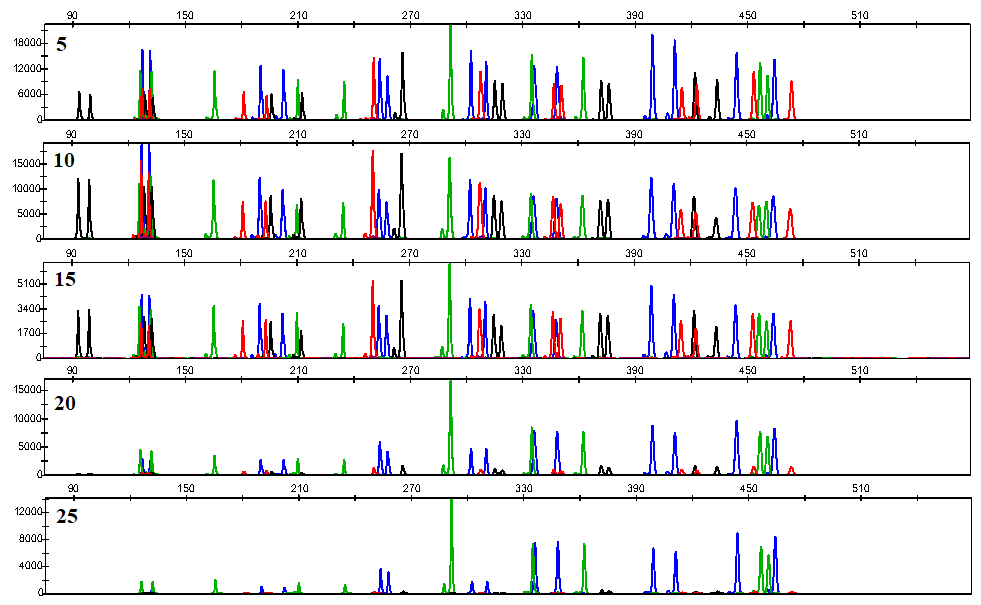

Supplement: Supplementary file 1 [file DataSheet1.ZIP › SM/Supplementary Figure 7.tif]

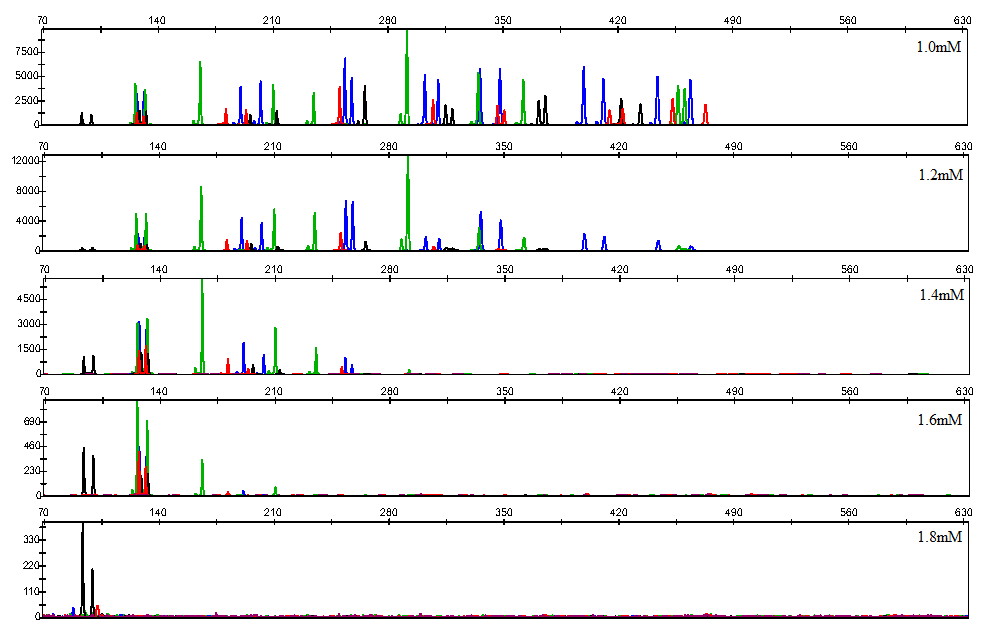

Supplement: Supplementary file 1 [file DataSheet1.ZIP › SM/Supplementary Figure 8.tif]

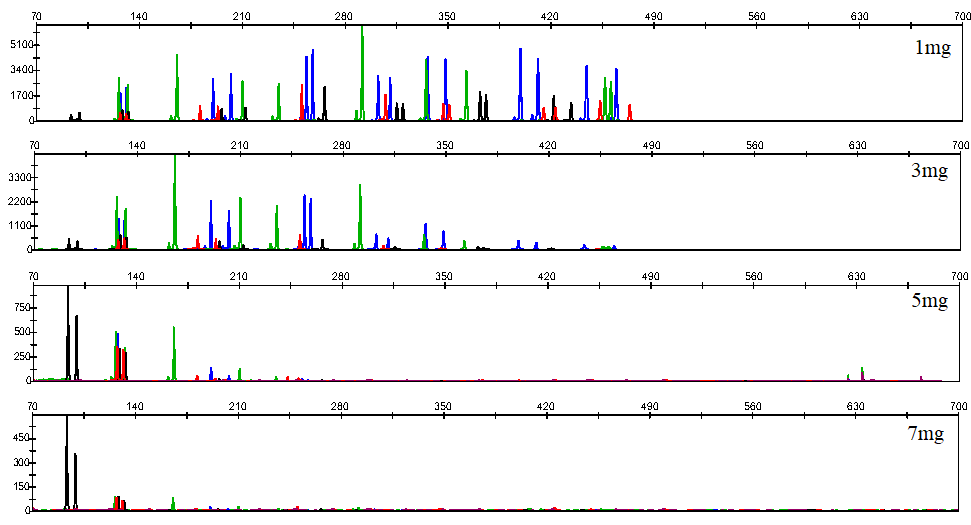

Supplement: Supplementary file 1 [file DataSheet1.ZIP › SM/Supplementary Figure 9.tif]
